# Utilizing *Caenorhabditis Elegans* as a Rapid and Precise Model for Assessing Amphetamine‐Type Stimulants: A Novel Approach to Evaluating New Psychoactive Substances Activity and Mechanisms

**DOI:** 10.1002/advs.202500808

**Published:** 2025-03-11

**Authors:** Yuanpeng Li, Hongyuan Li, Hongshuang Wang, Xiaohui Wang

**Affiliations:** ^1^ Laboratory of Chemical Biology Changchun Institute of Applied Chemistry Chinese Academy of Sciences Changchun Jilin 130022 China; ^2^ School of Applied Chemistry and Engineering University of Science and Technology of China Hefei Anhui 230026 China; ^3^ State Key Laboratory of Brain Machine Intelligence Zhejiang University Hangzhou 310027 China

**Keywords:** amphetamine‐type stimulants, dopaminergic and serotonergic pathways, new psychoactive substances, structure‐activity relationships, swimming‐induced paralysis

## Abstract

The surge of new psychoactive substances (NPS) poses significant public health challenges due to their unregulated status and diverse effects. However, existing in vivo models for evaluating their activities are limited. To address this gap, this study utilizes the model organism *Caenorhabditis elegans* (*C. elegans*) to evaluate the activity of amphetamine‐type stimulants (ATS) and their analogs. The swimming‐induced paralysis (SWIP) assay is employed to measure the acute responses of *C. elegans* to various ATS, including amphetamine (AMPH), methamphetamine (METH), 3,4‐methylenedioxymethamphetamine (MDMA) and their enantiomers. The findings reveal distinct responses in wild‐type and mutant *C. elegans*, highlighting the roles of dopaminergic and serotonergic pathways, particularly DOP‐3 and SER‐4 receptors. The assay also revealed that *C. elegans* can distinguish between the chiral forms of ATS. Additionally, structural activity relationships (SAR) are observed, with *meta*‐R amphetamines showing more pronounced effects than *ortho*‐R and *para‐*R analogs. This study demonstrates the utility of *C. elegans* in rapidly assessing ATS activity and toxicity, providing a cost‐effective and precise method for high‐throughput testing of NPS. These results contribute to a better understanding of ATS pharmacology and offer a valuable framework for future research and potential regulatory applications.

## Graphical abstract

This study uses *Caenorhabditis elegans* to assess the activity of amphetamine‐type stimulants (ATS) and their analogs. The research highlights the roles of dopamine and serotonin pathways, distinguishes chiral forms of ATS, and identifies structure‐activity relationships. These findings provide a rapid and cost‐effective model for evaluating the effects of new psychoactive substances.

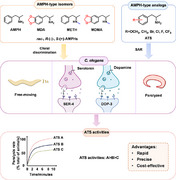

## 1 Introduction

The availability of a wide variety of new psychoactive substances (NPS) has surged.^[^ ^1^ ^]^ The United Nations Office on Drugs and Crime (UNODC) defines NPS as substances of abuse that are unregulated by the 1961 or 1971 conventions but pose public health risks. The term “new” refers to their recent market availability rather than recent invention. To date, 1245 NPS have been reported from 142 countries and territories, with stimulants being one of the most abused illicit drugs globally.^[^ ^2^ ^]^ The classes of NPS often reflect those most frequently reported in consumer group surveys, such as amphetamine‐type stimulants (ATS).^[^ ^3^ ^]^

ATS, a group of synthetic sympathomimetic amines, typically produces significant stimulant effects on the central nervous system. This group includes substances like amphetamine (AMPH), methamphetamine (METH, “ice”), and compounds from the “ecstasy” group such as 3,4‐methylenedioxymethamphetamine (MDMA, “ecstasy”) and its analogs. ATS are the second most popular group of drugs worldwide after cannabis,^[^ ^2^ ^]^ and a significant proportion of NPS with stimulant effects are ATS. In 2022, it was estimated that over 30 million people used amphetamines, and ≈20 million people used “ecstasy” globally.^[^ ^4^ ^]^

The rise of NPS has presented significant challenges, including the vast number of substances classified under this category, their rapid turnover in drug markets, and widespread misinformation and lack of awareness about their contents. Additionally, the diverse and often unknown potency, effects, and risk profiles of these substances complicate the issue further.^[^ ^5^ ^]^ Acute or long‐term use of NPS can result in severe health problems, such as neuroinflammation,^[^ ^6^, ^7^ ^]^ sleep disturbances,^[^ ^8^ ^]^ dangerously high body temperature,^[^ ^9^ ^]^ psychosis,^[^ ^10^ ^]^ delusions,^[^ ^11^ ^]^ addiction,^[^ ^12^ ^]^ and cardiac disease.^[^ ^13^ ^]^ Therefore, a rapid and precise model for assessing ATS is crucial for public health.

The primary effects of a stimulant are believed to be dictated by its predominant action on serotonin versus dopamine transporters.^[^ ^14^ ^]^ Previous studies have utilized cell lines expressing the human isoforms of serotonin transporter (SERT), dopamine transporter (DAT), and norepinephrine transporter (NET) to test neurotransmitter uptake and release following treatment with ATS.^[^ ^15^ ^]^ However, due to the complexity o*f* in vivo systems, there are inherent limitations in extrapolating in vitro data to in vivo activity studies.^[^ ^16^, ^17^ ^]^

*Caenorhabditis elegans* (*C. elegans*), a classic animal model, is commonly used due to its convenience in genetic study and cultivation.^[^ ^18^ ^]^ With only 302 neurons, *C. elegans* can perform a variety of behaviors, making it suitable for rapid testing of ATS activity at the individual level. A robust behavior called swimming‐induced paralysis (SWIP) has been observed when *C. elegans* is treated with amphetamine (AMPH).^[^ ^19^, ^20^ ^]^ AMPH‐induced SWIP occurs due to an excess of dopamine in the synapses, which overstimulates the D2 post‐synaptic receptors (DOP‐3), leading to reversible paralysis in the *C. elegans*.^[^ ^21^, ^22^ ^]^ Furthermore, repeated exposure to AMPH reduces SWIP behaviors, indicating that *C. elegans* can be used to study AMPH tolerance.^[^ ^20^ ^]^

In this study, the behaviors of *C. elegans* were measured under various ATS treatments, including enantiomers of classical ATS and NPS. It was found that not only AMPH but also other ATS rapidly induced abnormal locomotion in wild‐type animals. These treatments resulted in different behavioral changes in dopaminergic and serotonergic pathway loss‐of‐function mutants in a time‐dose‐dependent manner. Additionally, the findings highlight the roles of the 5‐HT receptor SER‐4 and the dopamine receptor DOP‐3 in mediating ATS activities. Moreover, structure‐activity relationships (SAR) were observed, with *meta*‐R amphetamines demonstrating more pronounced effects compared to *ortho*‐R and *para‐*R analogs. In summary, this study identifies a robust system for rapidly evaluating the activity of ATS at the individual level.

## 2 Results

### 2.1 ATS Rapidly Induce Abnormal Mobility in *C. Elegans*

**Figure 1:**
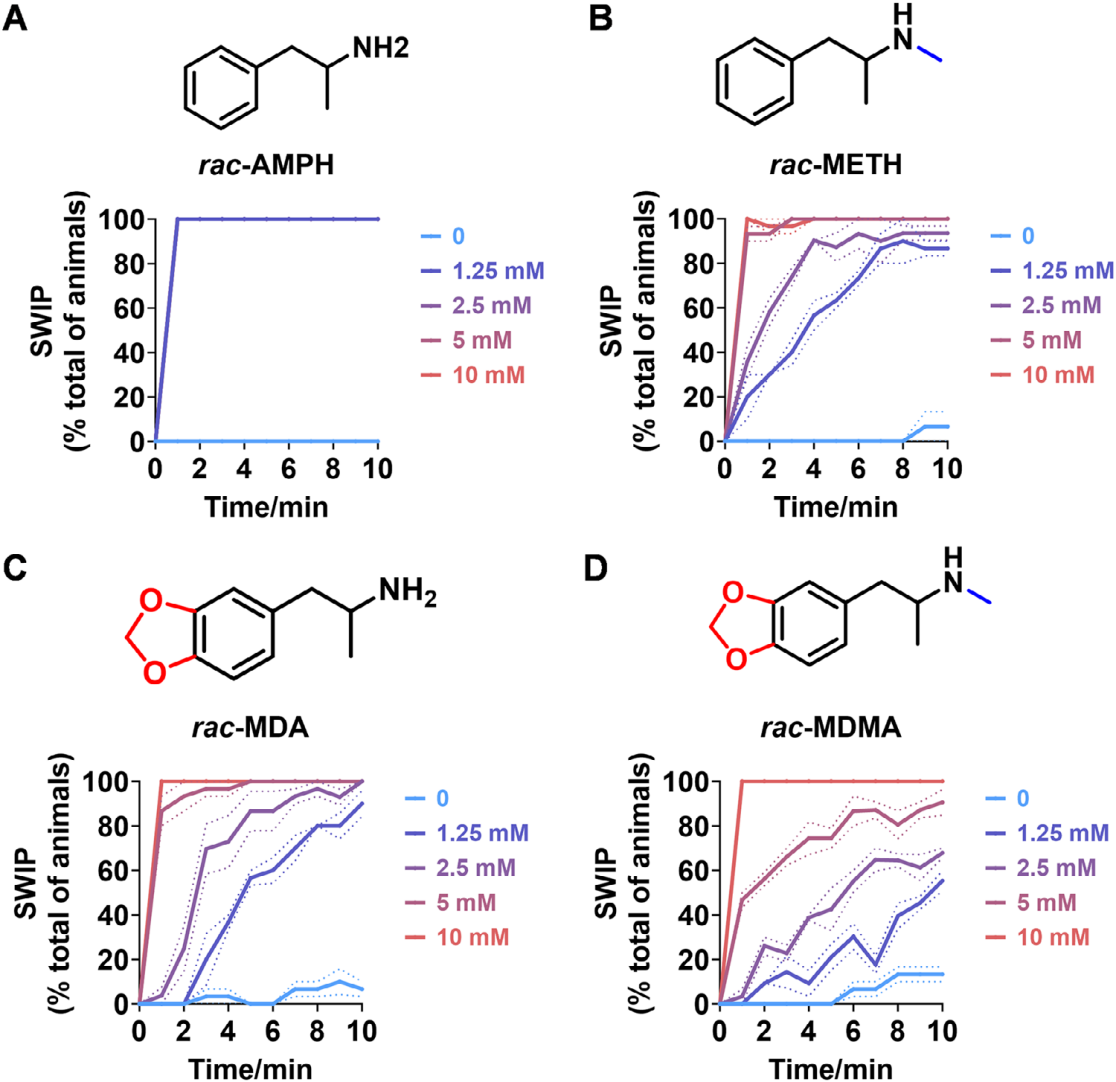
SWIP behavior induced by classic AMPHs. A–D) The SWIP behavior was observed in N2 when exposed to varying concentrations (0, 1.25, 2.5, 5, and 10 mm) of *rac*‐AMPH (A), *rac*‐METH (B), *rac*‐MDA (C), and *rac*‐MDMA (D). The behaviors were assessed in a time‐dose‐dependent manner over 10 min. Three independent experiments were conducted.

Previous research has demonstrated that the stimulant AMPH has a strong inhibitory effect on the swimming ability of *C. elegans*, a phenomenon known as SWIP.^[^ ^21^ ^]^ To assess the impact of ATS, the SWIP assay was used to examine their effects on *C. elegans*. As shown in **Figure** **1**A,B, varying inhibitory effects on the swimming ability of *C. elegans* were observed when exposed to different concentrations of AMPH (Video S1, Supporting Information) and METH (Video S2, Supporting Information), a methylated derivative of AMPH. Specifically, at concentrations of 1.25, 2.5, and 5 mm, *C. elegans* exhibited total paralysis in the AMPH‐treated group after 1 min (Figure 1A), whereas only partial paralysis was seen in the METH‐treated group (Figure 1B). At a concentration of 10 mm, total paralysis occurred in both the AMPH and METH‐treated groups after 1 min (Figure 1A,B). At lower concentrations of 0.01, 0.1, and 1 mm, *C. elegans* showed partial paralysis in the AMPH‐treated group after 1 min (Figure S1A, Supporting Information). These findings suggest that *C. elegans* have a more pronounced response to AMPH‐induced SWIP compared to METH.

Interestingly, similar inhibitory effects on the swimming ability of *C. elegans* were observed when exposed to 3,4‐methylenedioxyamphetamine (MDA) (Video S3, Supporting Information) and MDMA (Video S4, Supporting Information), with a more significant response to MDA‐induced SWIP compared to MDMA. As shown in Figure 1C,D, at concentrations of 5 and 10 mm, *C. elegans* experienced total paralysis in the MDA‐treated group after 6 min, while partial paralysis was seen in the MDMA‐treated group at concentrations of 5 mm. Additionally, at a concentration of 1.25 mm, the proportion of paralyzed *C. elegans* was higher in the MDA‐treated group compared to the MDMA group throughout the measured time (Figure 1C,D). A previous study showed that MDMA could cause pronounced paralysis characterized by the head being locked in a straight position and continuous crawling toward the ventral direction.^[^ ^23^ ^]^ To measure this response, *C. elegans* were placed on agar plates containing varying concentrations of METH or MDMA. Intriguingly, both MDMA‐ and METH‐induced abnormal crawling behavior, characterized by a “neck‐locked” posture and ventral‐dorsal movement, in a dose‐dependent manner (Videos S5 and S6 and Figure S2, Supporting Information). However, other ATS (including AMPH and MDA) did not exhibit this abnormal crawling behavior (Videos S7 and S8, Supporting Information).

In conclusion, *C. elegans* exhibit a more significant response to AMPH over METH and to MDA over MDMA in terms of SWIP, indicating a compound‐specific susceptibility and highlighting their suitability as a model for evaluating the activity of classic ATS.

### 2.2 METH and MDMA Cause Abnormal Mobility by Acting on Dopaminergic and Serotonergic Pathway

**Figure 2:**
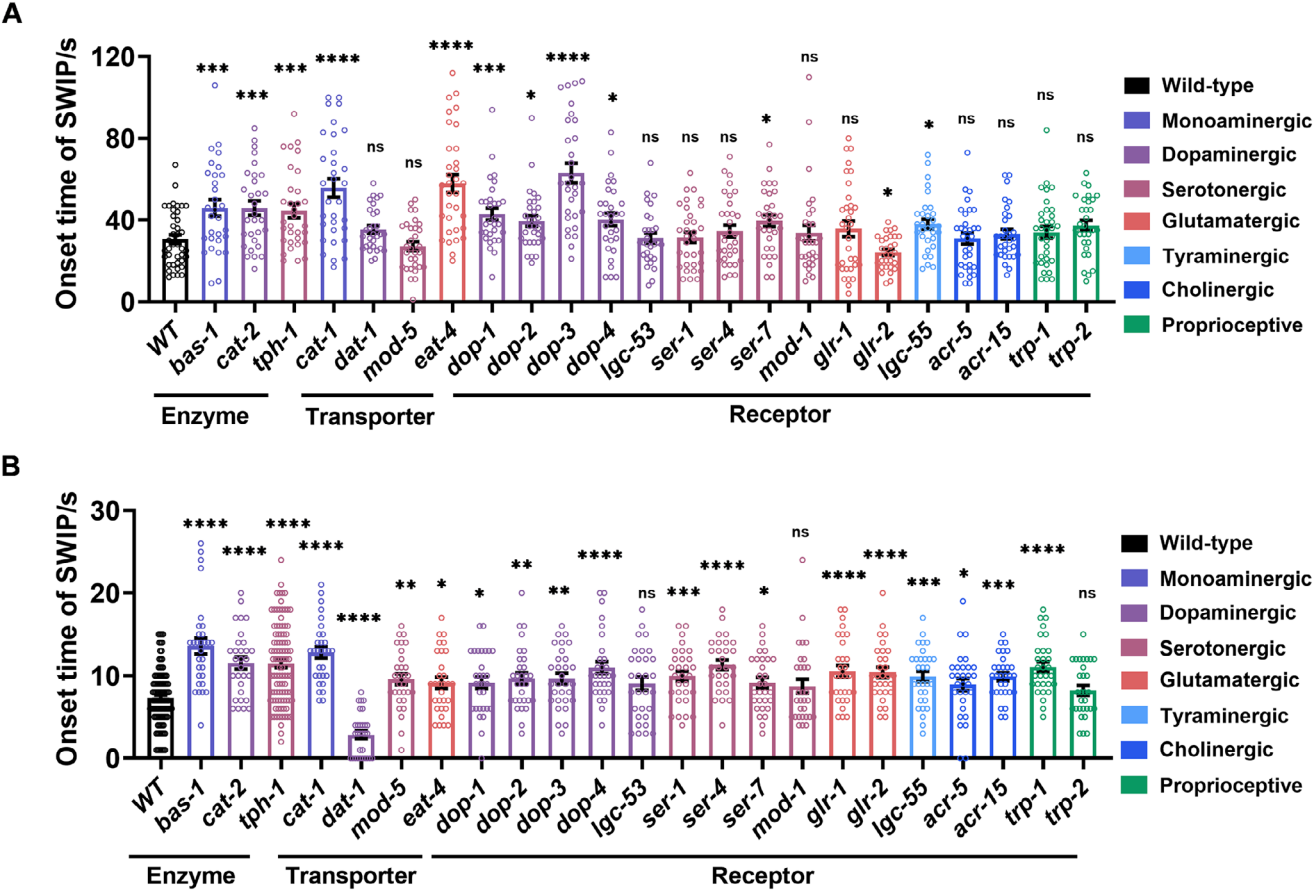
SWIP behavior induced by classical ATS in neurotransmitter‐related gene mutants. A,B) The SWIP behavior was assessed in neurotransmitter‐related gene mutants when exposed to 15 mm *S*‐(+)‐METH (A) and 10 mm *rac*‐MDMA (B). Mutants involved in dopamine and serotonin production, monoamine transport, neurotransmitter receptor activity, and transient receptor potential channel function were studied. Three independent experiments were conducted. N2 were used as the relevant control group. Statistical significance is indicated as follows: ns, *p *> 0.05; ^*^ *p *< 0.05; ^**^ *p *< 0.01; ^***^ *p *< 0.001; ^****^ *p *< 0.0001.

The signaling of neurotransmitters is essential in the behaviors induced by psychostimulants. Previous studies have shown that AMPH‐induced SWIP requires DAT‐1, LGC‐53, and LGC‐55,^[^ ^19^, ^21^ ^]^ while β‐phenylethylamine (β‐PEA)‐induced SWIP involves CAT‐2, DAT‐1, DOP‐3, LGC‐53, and LGC‐55.^[^ ^24^ ^]^ To elucidate the primary mechanisms behind ATS‐induced SWIP, gene mutant *C. elegans* were used in these behavioral assays. Day‐1 adult *C. elegans* were exposed to METH or MDMA, and the onset of the first SWIP behavior was recorded. As shown in **Figure** **2**A,B, loss of function in the *bas‐1*, *cat‐2*, and *tph‐1* genes, which are involved in dopamine and serotonin production, significantly prolonged paralysis time induced by METH or MDMA. Conversely, different effects were observed in gene mutants involved in the transport of monoamines (dopamine, serotonin, tyramine, octopamine). Loss of function in *cat‐1* and *eat‐4* markedly extended the paralysis time induced by METH (Figure 2A) or MDMA (Figure 2B), whereas loss of function in *dat‐1* and *mod‐5* had minimal impact on METH‐induced paralysis (Figure 2A). Additionally, *mod‐5* loss of function significantly prolonged MDMA‐induced paralysis (Figure 2B), while *dat‐1* loss of function had the opposite effect on MDMA‐induced paralysis (Figure 2B). These results suggest that the monoamine transport process has differential effects on METH and MDMA‐induced *C. elegans* paralysis.

Receptors play a crucial role in regulating signal transduction from neurotransmitters.^[^ ^25^ ^]^ As shown in Figure 2A,B, loss of function in dopamine receptors (*dop‐1*, *dop‐2*, *dop‐3*, *dop‐4*) significantly prolonged paralysis time induced by METH (Figure 2A) or MDMA (Figure 2B). In addition, loss of function in serotonin receptors (*ser‐1*, *ser‐4*,) had little effect on METH‐induced paralysis (Figure 2A) while it prolonged MDMA‐induced paralysis (Figure 2B). Similarly, loss of function in other receptor types, including glutamate receptors (*glr‐1*), ligand‐gated ion channels (*acr‐5*, *acr‐15*, *lgc‐53*, *mod‐1*, *lgc‐55*), and transient receptor potential calcium channels (*trp‐1*, *trp‐2*), had minimal effect on METH‐induced paralysis (Figure 2A). However, most of these receptor losses prolonged MDMA‐induced paralysis (Figure 2B), except for *lgc‐53*, *mod‐1*, and *trp‐2*, which had little effect on MDMA‐induced paralysis (Figure 2B).

In conclusion, these findings suggest that the dopamine system specifically mediates METH‐induced paralysis, whereas a broader range of systems, including dopamine, serotonin, glutamate, and acetylcholine systems, mediate MDMA‐induced paralysis. Notably, two neurotransmitter receptors, DOP‐3 and SER‐4, play crucial roles in SWIP, with DOP‐3 primarily influencing METH‐induced behaviors and SER‐4 predominantly associated with MDMA‐induced effects.

### 2.3 Chiral Discrimination of ATS Using the SWIP Assay in *C. Elegans*

**Figure 3:**
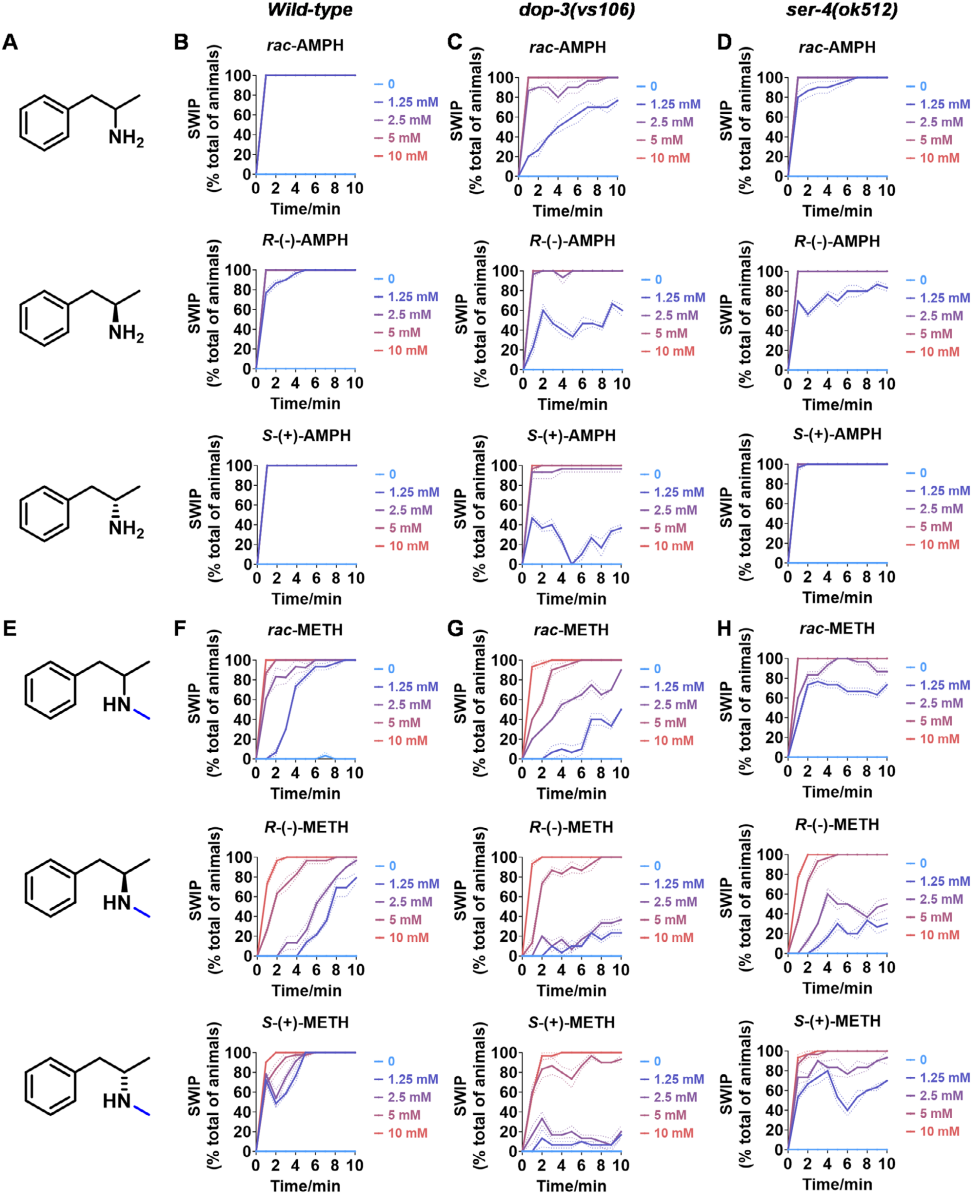
Chiral activity of AMPH‐ and METH‐induced SWIP behavior in *C. elegans*. A) Chemical structures of *rac*‐AMPH, *R*‐(‐)‐AMPH, and *S*‐(+)‐AMPH. B–D) SWIP behavior induced by various concentrations (0, 1.25, 2.5, 5, and 10 mm) of *rac*‐AMPH, *R*‐(‐)‐AMPH, and *S*‐(+)‐AMPH in: (B) N2, (C) *dop‐3* mutants, (D) *ser‐4* mutants. Three independent experiments were performed. E) Chemical structures of *rac*‐METH, *R*‐(‐)‐METH, and *S*‐(+)‐METH. (F‐H) SWIP behavior induced by various concentrations (0, 1.25, 2.5, 5, and 10 mm) of *rac*‐METH, *R*‐(‐)‐METH, and *S*‐(+)‐METH in: (H) N2, (G) *dop‐3* mutants, (H) *ser‐4* mutants. Three independent experiments were performed.

ATS possess a chiral center, resulting in the existence of two enantiomers and a racemic mixture.^[^ ^26^ ^]^ These forms can exhibit different pharmacological properties. Therefore, it is crucial to determine if there is a distinct response in *C. elegans* exposed to the different chiral forms of ATS. As shown in **Figure** **3**A,B when *C. elegans* were treated with varying concentrations of *R*‐(‐)‐AMPH, *S*‐(+)‐AMPH, and *rac*‐AMPH (1 and 1.25 mm), there was a noticeable difference in the response. *C. elegans* exposed to *R*‐(‐)‐AMPH at these concentrations showed a lower rate of paralysis compared to those treated with *S*‐(+)‐AMPH and *rac*‐AMPH. Notably, this difference was absent in *dop‐3* mutants (Figure 3C; Figure S1B, Supporting Information), while it persisted in *ser‐4* mutants (Figure 3D; Figure S1C, Supporting Information). These findings suggest that AMPH exhibits different chiral activity in *C. elegans*, particularly affecting the SWIP phenotype, and that *dop‐3* is critical in recognizing this activity.

Similarly, *C. elegans* treated with *R*‐(‐)‐METH and *S*‐(+)‐METH at specific concentrations (1.25 , 2.5 , 5 , and 10 mm) also exhibited different responses. *R*‐(‐)‐METH led to less paralysis than *S*‐(+)‐METH and *rac*‐METH (Figure 3E,F). Interestingly, this difference was eliminated in *dop‐3* mutants (Figure 3G) but not in *ser‐4* mutants (Figure 3H), indicating that *dop‐3*, but not *ser‐4*, primarily recognizes the chiral activity of METH in the SWIP phenotype, similar to its role in recognizing AMPH's chiral activity.

In addition, *C. elegans* treated with *R*‐(‐)‐MDA and *S*‐(+)‐MDA displayed different responses, with *R*‐(‐)‐MDA causing less paralysis than *S*‐(+)‐MDA and *rac*‐MDA (Figure S3A,B, Supporting Information). Notably, both *dop‐3* and *ser‐4* were involved in recognizing the chiral activity of MDA in the SWIP phenotype, as the difference in response to *R*‐(‐)‐MDA and *S*‐(+)‐MDA was abolished in *dop‐3* (Figure S3C, Supporting Information) and *ser‐4* (Figure S3D, Supporting Information) mutants. Conversely, there was little variation in response between *R*‐(‐)‐MDMA and *S*‐(+)‐MDMA‐treated *C. elegans* at specified concentrations (1.25 , 2.5 , 5 , and 10 mm) (Figure S4A,B, Supporting Information). Both forms of MDMA‐induced similar responses compared to *rac*‐MDMA in the SWIP assay. Additionally, loss of *dop‐3* function had minimal impact on the time to paralysis induced by *rac*‐MDMA, *R*‐(‐)‐MDMA, and *S*‐(+)‐MDMA at the indicated concentrations (1.25, 2.5, 5, 10 mm) (Figure S4C, Supporting Information). However, a loss in *ser‐4* downregulated the rate of paralysis in *C. elegans* treated with *rac*‐MDMA, *R*‐(‐)‐MDMA, and *S*‐(+)‐MDMA at lower concentrations (1.25 , 2.5 mm), with limited effect at higher concentrations (5, 10 mM) (Figure S4D, Supporting Information).

In summary, the SWIP assay in *C. elegans* effectively recognizes the chiral activity of ATS, with the involvement of *dop‐3* or *ser‐4* gene functions in distinguishing these chiral activities.

### 2.4 Evaluating the Activity of AMPH‐Type Analogs Using *C. Elegans* SWIP Behavior Assay

**Figure 4:**
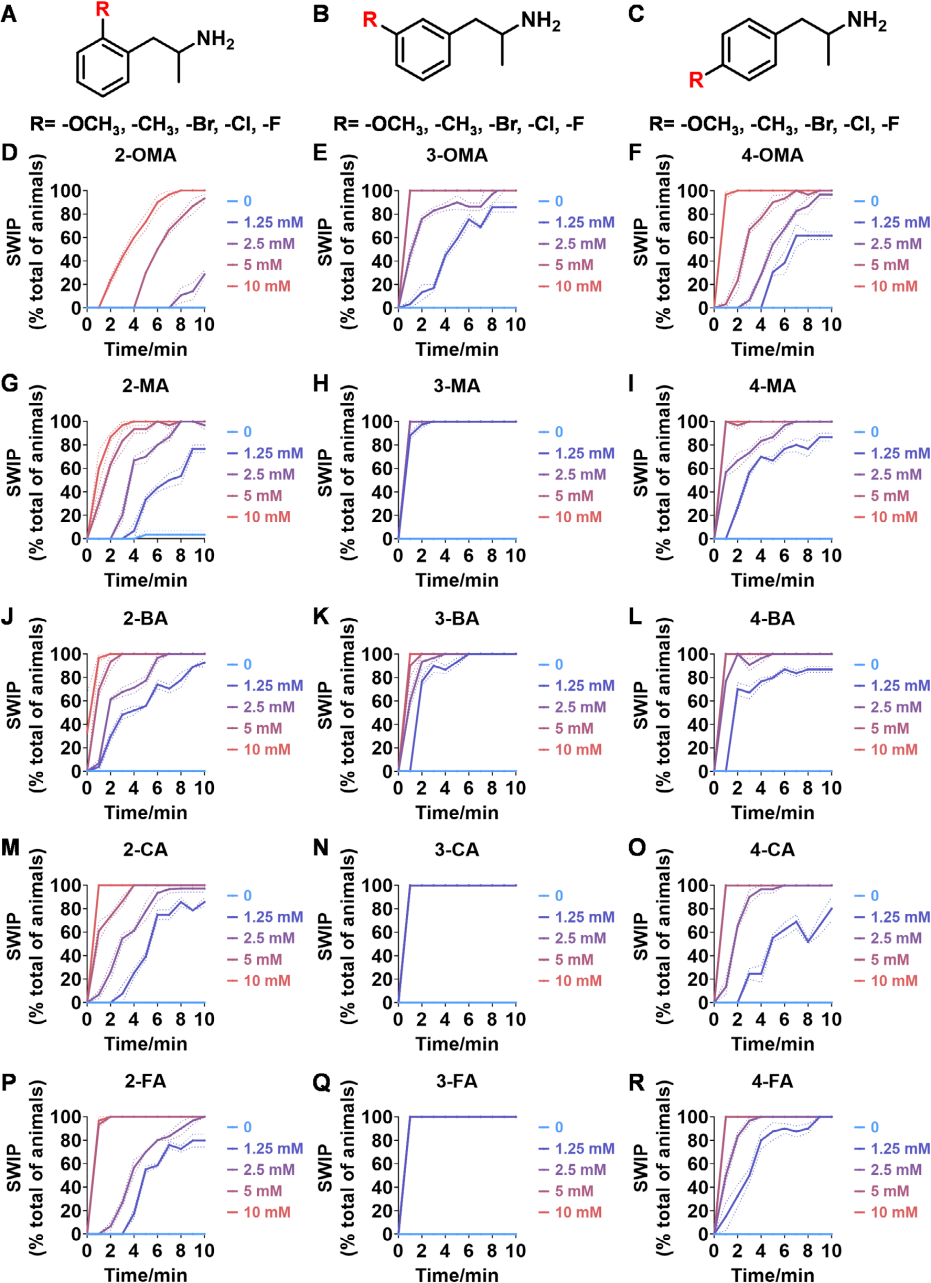
Structural and functional comparison of ATS‐induced SWIP behavior in *C. elegans*. A–C) Chemical structures of OCH_3_, CH_3_, Br, Cl, F substituents at the *ortho*‐, *meta‐*, and *para‐*position in AMPH. D–F) OCH_3_ substituents *ortho*‐ (**2‐OMA**, D), *meta‐* (**3‐OMA**, E), and *para‐*position (**4‐OMA**, F) in AMPH and their related SWIP behavior at concentrations of 0, 1.25, 2.5, 5, and 10 mm in N2. G–I) CH_3_ substituents *ortho*‐ (**2‐MA**, G), *meta‐* (3‐MA, H), and *para‐*position (**4‐MA**, I) in AMPH and their related SWIP behavior at concentrations of 0, 1.25, 2.5, 5, and 10 mm in N2. Three independent experiments were conducted. J–L) Br substituents *ortho*‐ (**2‐BA**, J), *meta‐* (**3‐BA**, K), and *para‐*position (**4‐BA**, L) in AMPH and their related SWIP behavior at concentrations of 0, 1.25, 2.5, 5, and 10 mm in N2. M–O) Cl substituents *ortho*‐ (2‐CA, M), *meta‐* (3‐CA, N), and *para‐*position (4‐CA, O) in AMPH and their related SWIP behavior at concentrations of 0, 1.25, 2.5, 5, and 10 mm in N2. P–R) F substituents *ortho*‐ (**2‐FA**, P), *meta‐* (**3‐FA**, Q), and *para‐*position (**4‐FA**, R) in AMPH and their related SWIP behavior at concentrations of 0, 1.25, 2.5, 5, and 10 mm in N2. Three independent experiments were conducted.

AMPH‐type analogs encompass a wide range of substances, including stimulants, psychedelic compounds, and other related compounds. Given the significant presence of ATS in NPS,^[^ ^2^ ^]^ it is essential to have an effective method for rapidly evaluating AMPH‐like derivatives. As demonstrated in **Figures** **4** and Figure S5 (Supporting Information), the SWIP behavior varied when *C. elegans* were treated with derivatives having substituent groups at the *ortho*, *meta*, and *para* positions in AMPH (Figure 4A–C).

A negative correlation between the activity of AMPH derivatives and the electron density of the benzene ring was observed. Specifically, as the electron‐donating capacity decreases among the substituents (OCH_3_ > CH_3_), AMPH derivatives with methoxy substitutions (Figure 4D–F) consistently exhibited lower activity in inducing paralysis compared to those with methyl substitutions (Figure 4G–I). To further validate this observation, AMPH derivatives with dimethoxy substitutions were measured (Figure S6A–C, Supporting Information) and found an even further reduction in SWIP activity compared to their monomethoxy counterparts (Figure 4D–F). This suggests that among the electron‐donating groups, AMPH derivatives with methyl substitutions, which impart a weaker electron density to the benzene ring, are more active than those with methoxy or dimethoxy groups.

This trend was also observed for electron‐withdrawing groups. As the electron‐withdrawing capacity increased among the substituents (Br < Cl < F), the activity of AMPH derivatives in inducing paralysis and affecting the percentage of *C. elegans* was decreased in the derivatives substituted with bromine (Figure 4J–L) or chlorine (Figure 4M–O; Figure S5B, Supporting Information) compared to those with fluorine (Figure 4P–R; Figure S5C, Supporting Information) at the same position. This finding indicates that, among electron‐withdrawing groups, AMPH derivatives with fluorine substitution exhibit higher activity than those with chlorine or bromine, consistent with fluorine's stronger electron‐withdrawing capacity. Therefore, strong electron‐withdrawing trifluoromethyl (CF_3_) substituted AMPH derivatives were synthesized and measured. However, despite the CF_3_ group having a stronger electron‐withdrawing effect than fluorine, AMPH derivatives with trifluoromethyl substitution (Figure S6D–F, Supporting Information) did not exhibit the highest activity. This discrepancy may be attributed to the steric hindrance introduced by the bulkier trifluoromethyl group, which likely reduces the derivative's overall activity.

Additionally, a consistent pattern regarding the influence of substitution positions on AMPH derivative activity was identified. Specifically, for single‐substituted compounds, the SWIP behavior in *C. elegans* consistently followed the trend: *meta*‐substituted > *para*‐substituted > *ortho*‐substituted. For instance, the activity of methoxy‐substituted AMPH derivatives in inducing paralysis and affecting the percentage of *C. elegans* followed the order: **3‐OMA** (Figure 4E) > **4‐OMA** (Figure 4F) > **2‐OMA** (Figure 4D). This pattern was observed across various substituents, including methyl (Figure 4G–I), bromine (Figure 4J–L), chlorine (Figure 4M–O), fluorine (Figure 4P–R), and trifluoromethyl groups (Figure S6D–F, Supporting Information). *Meta*‐substituted derivatives consistently showed the highest activity, followed by *para*‐ and then *ortho*‐substituted derivatives, suggesting a potential structural preference for enhanced biological effects in *C. elegans*.

**Figure 5:**
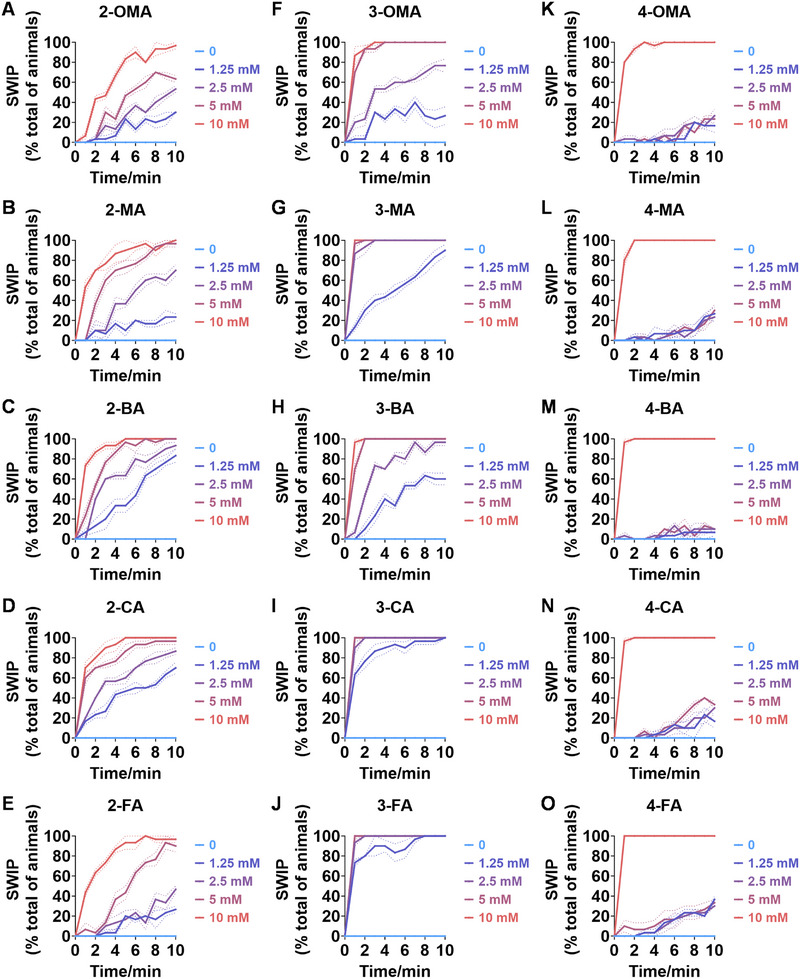
Structural and functional comparison of ATS‐induced SWIP behavior in *dop‐3* mutants. A–E) SWIP behaviors of OCH_3_ (**2‐OMA**, A), CH_3_ (**2‐MA**, B), Br (**2‐BA**, C), Cl (**2‐CA**, D), and F (**2‐FA**, E) substituents at the *ortho*‐position in AMPH at concentrations of 0, 1.25, 2.5, 5, and 10 mm in *dop‐3* mutants. F–J) SWIP behaviors of SWIP behaviors of OCH_3_ (**3‐OMA**, F), CH_3_ (**3‐MA**, G), Br (**3‐BA**, H), Cl (3‐CA, I), and F (**3‐FA**, J) substituents at the *meta*‐position in AMPH at concentrations of 0, 1.25, 2.5, 5, and 10 mm in *dop‐3* mutants. K–O) SWIP behaviors of SWIP behaviors of OCH_3_ (**4‐OMA**, K), CH_3_ (**4‐MA**, L), Br (**4‐BA**, M), Cl (**4‐CA**, N), and F (**4‐FA**, O) substituents at the *para‐*position in AMPH at concentrations of 0, 1.25, 2.5, 5, and 10 mm in *dop‐3* mutants. Three independent experiments were conducted.

**Figure 6:**
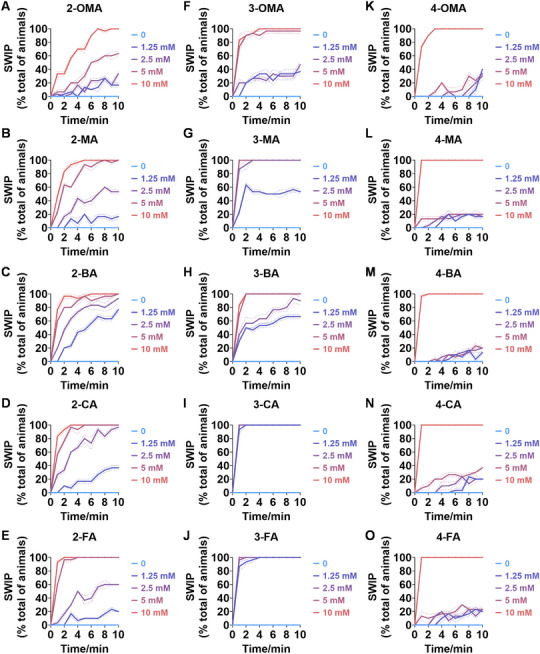
Structural and functional comparison of ATS‐induced SWIP behavior in *ser‐4* mutants. A–E) SWIP behaviors of OCH_3_ (**2‐OMA**, A), CH_3_ (**2‐MA**, B), Br (**2‐BA**, C), Cl (**2‐CA**, D), and F (**2‐FA**, E) substituents at the *ortho*‐position in AMPH at concentrations of 0, 1.25, 2.5, 5, and 10 mm in *ser‐4* mutants. F–J) SWIP behaviors of OCH_3_ (**3‐OMA**, F), CH_3_ (**3‐MA**, G), Br (**3‐BA**, H), Cl (**3‐CA**, I), and F (**3‐FA**, J) substituents at the *meta*‐position in AMPH at concentrations of 0, 1.25, 2.5, 5, and 10 mm in *ser‐4* mutants. K–O) SWIP behaviors of (**4‐OMA**, K), CH_3_ (**4‐MA**, L), Br (**4‐BA**, M), Cl (**4‐CA**, N), and F (**4‐FA**, O) substituents at the *para‐*position in AMPH at concentrations of 0, 1.25, 2.5, 5, and 10 mm in *ser‐4* mutants. Three independent experiments were conducted.

Additionally, to investigate the role of specific receptors in SWIP behavior induced by AMPH derivatives, the activities of these derivatives were measured in *C. elegans* mutants lacking either the DOP‐3 receptor (**Figure** **5**; Figures S5D–F and S6, Supporting Information) or the SER‐4 receptor (**Figure** **6**; Figures S5G–H and S7, Supporting Information), in comparison to N2 (Figure 4). Among the *ortho‐*position compounds, with the exception of **2‐OMA** (Figure 5A), other AMPH derivates such as **2‐MA** (Figure 5B), **2‐BA** (Figure 5C), **2‐CA** (Figure 5D), **2‐FA** (Figure 5E) and **2‐TFA** (Figure S7D, Supporting Information) showed varying degrees of reduced SWIP activity in *dop‐3* mutants, with **2‐FA** (Figure 5E) exhibiting the most significant decrease. A similar trend was observed in *ser‐4* mutants, where **2‐OMA** (Figure 6A) showed a slight increase in SWIP activity, while AMPH derivatives substituted with CH_3_, Br, Cl, F, CF_3_ (Figure 6B–E; Figure S8A, Supporting Information) indicated lower SWIP responses, with **2‐TFA** showing the largest reduction (Figure S8A, Supporting Information). These changes in mutants partially diminished the activity differences caused by the benzene ring's electron density.

For the *meta*‐substituted AMPH derivatives, SWIP activity was consistently downregulated in both *dop‐3* and *ser‐4* mutants across all compounds compared to N2 (Figure 4; Figure S5, Supporting Information). Specifically, 3‐OMA (Figure 5F), **3‐MA** (Figure 5G; Figure S5D, Supporting Information), **3‐BA** (Figure 5H), **3‐CA** (Figure 5I; Figure S5E, Supporting Information), **3‐FA** (Figure 5J; Figure S5F, Supporting Information) and **4‐TFA** (Figure S7B, Supporting Information) showed decreased SWIP activity in *dop‐3* mutants while exhibited similar reductions in *ser‐4* mutants (Figure 6F–J; Figures S5G,H and S8B, Supporting Information). However, the activity differences due to electron density on the benzene ring remained evident. Among these, **3‐BA** (Figures 5H and 6H) and **3‐TFA** (Figures S7B and S8B, Supporting Information) showed the most pronounced reduction in SWIP activity.

In contrast, for *para*‐substituted AMPH derivatives, all compounds demonstrated significant changes in SWIP activity compared to N2 (Figure 4), effectively eliminating the activity differences attributed to benzene ring electron density. In *dop‐3* mutants, the paralysis rate within 10 min for all ATS compounds (**4‐OMA** (Figure 5K), **4‐MA** (Figure 5L), **4‐BA** (Figure 5M), **4‐CA** (Figure 5N), **4‐FA** (Figure 5O), **4‐TFA** (Figure S7C, Supporting Information) at concentrations of 1.25, 2.5, and 5 mm was less than 40%. Similarly, in *ser‐4* mutants, the same conclusion was observed for **4‐OMA** (Figure 6K), **4‐MA** (Figure 6L), **4‐BA** (Figure 6M), **4‐CA** (Figure 6N) and **4‐FA** (Figure 6O) except **4‐TFA** (Figure S8C, Supporting Information).

For the dimethoxy‐substituted compounds, the activities of **3,4‐di‐OMA** and **2,5‐di‐OMA** were significantly reduced in both *dop‐3* (Figure S7D,E, Supporting Information) and *ser‐4* (Figure S8D,E, Supporting Information) mutants compared to wild‐type (Figure S6A,B, Supporting Information). However, **2,5‐di‐OMA** exhibited no significant change in SWIP activity in *dop‐3* (Figure S7F, Supporting Information) mutants, while showing a slight increase at 1.25  and 2.5 mm concentrations in *ser‐4* mutant (Figure S8F, Supporting Information) compared to wild‐type (Figure S6C, Supporting Information). Notably, the variation of **2,5‐di‐OMA** in *ser‐4* mutant (Figure S8F, Supporting Information) is similar to **2‐OMA** (Figure 6A). This suggests that the substitution of methoxy groups at the *ortho*‐position may interact with SER‐4 in a manner distinct from other substituents.

In summary, DOP‐3 and SER‐4 play a crucial role in mediating the activities of AMPH derivatives, with a particularly notable impact on *para*‐substituted AMPH derivatives.

## 3 Discussion

ATS represents a significant class of psychoactive substances with diverse pharmacological effects. Understanding the acute responses and underlying mechanisms of ATS‐induced behaviors is crucial for addressing their potential for abuse. The nematode model organism, *C. elegans*, offers a valuable system for studying these effects due to its genetic tractability and well‐characterized nervous system. This study elucidated the acute response of *C. elegans* to ATS, emphasizing the roles of dopaminergic and serotonergic pathways. It also demonstrated the utility of the SWIP assay in evaluating ATS‐induced behaviors, chiral discrimination, and SAR. These findings provide a rapid, precise, and cost‐effective method for measuring ATS activity, offering a valuable framework for future investigations and potential applications in high‐throughput evaluation of NPS.

In this study, ATS exposure rapidly induced abnormal locomotion in *C. elegans*, initially decreasing their thrashing frequency and eventually causing paralysis. Previous research suggests that amphetamines primarily target neurotransmitter sodium symporters (NSS), which transport monoamines such as dopamine (DAT/SLC6A3), serotonin (SERT/SLC6A4),^[^ ^27^ ^]^ and noradrenaline/norepinephrine (NET/SLC6A2).^[^ ^27^ ^]^ In *C. elegans*, AMPH‐induced paralysis is triggered by promoting dopamine efflux.^[^ ^21^ ^]^ Our investigation further revealed that ATS‐induced behaviors are primarily mediated by the dopaminergic and serotonergic systems, with DOP‐3 and SER‐4 receptors playing key roles. The mechanistic roles of DOP‐3 and SER‐4 in mediating drug‐induced behaviors in *C. elegans* are underscored by their human orthologs, dopamine receptors D2/D3 and serotonin 1A receptor (5‐HT1A), respectively. Loss of DOP‐3 function attenuates METH‐induced SWIP, mirroring findings in D2R knockout mice, where protection against METH neurotoxicity involves vesicular dopamine release blockade and body temperature modulation.^[^ ^28^ ^]^ Moreover, D3R deficiency inhibits GSK3β activity and reduces miR‐29c expression, diminishing METH's reward effects.^[^ ^29^ ^]^ Clinically, reduced striatal D2/D3 receptor availability in METH use disorder further supports their mechanistic relevance.^[^ ^30^ ^]^ For SER‐4, its loss‐of‐function attenuates MDMA‐induced SWIP, aligning with 5‐HT1A receptor as one of the molecular targets in MDMA's neuropharmacology.^[^ ^31^ ^]^ MDMA increases extracellular serotonin by inhibiting serotonin transporters, which activate the inhibitory 5‐HT1A receptor on serotonin neurons, suppressing the activity of dorsal raphe nucleus (DRN) serotonin neurons.^[^ ^32^ ^]^ Similarly, in *C. elegans*, SER‐4 activation likely triggers inhibitory neural pathways, contributing to SWIP. Indeed, excessive serotonin can induce SWIP.^[^ ^33^ ^]^ This suggests that SER‐4 activation in *C. elegans* may disrupt sensory neurons, indirectly affecting motor control circuits. Together, these insights highlight the conserved mechanistic pathways through which DOP‐3 and SER‐4 mediate ATS‐induced behavioral and physiological responses.

Additionally, our study identified specific roles for other genes, such as *eat‐4* and *trp‐1*, in mediating ATS‐induced behaviors. The vesicular glutamate transporter encoded by *eat‐4*, an ortholog of mammalian SLC17A7 and SLC17A8, has been implicated in glutamate dysregulation associated with drug dependence.^[^ ^34^ ^]^ Research in mammals has shown that METH administration increases the expression of VGLUT1 in the cortex and VGLUT2 in the striatum,^[^ ^35^ ^]^ suggesting a potential role for glutamate signaling in vulnerability to drug dependence. In *C. elegans*, METH‐induced dysregulation of glutamate transmission via *eat‐4* may involve increased glutamate release or altered vesicular glutamate packaging. Additionally, the interaction between glutamate and dopamine systems may converge on common downstream circuits or molecular targets to produce the overall behavioral response to METH. These findings underscore the importance of *eat‐4* in modulating METH‐induced behaviors. Similarly, *trp‐1*, a transient receptor potential (TRP) channel in *C. elegans*, appears to play a specific role in MDMA‐induced effects. TRP‐1 is a cation channel involved in regulating neuronal excitability and calcium signaling, which are fundamental to neuronal communication and behavior.^[^ ^36^ ^]^ It is expressed in motor neurons, interneurons, sensory neurons, and muscles,^[^ ^37^, ^38^ ^]^ and its interaction with *trp‐2* is critical for head steering during forward locomotion.^[^ ^39^ ^]^ Mutants lacking both *trp‐1* and *trp‐2* exhibit defects in steering, resulting in ventral circling, a behavior reminiscent of the “neck locked” posture and ventral‐dorsal movement in *C. elegans*.^[^ ^39^ ^]^ This suggests that *trp‐1* may be directly or indirectly modulated by MDMA, altering its channel properties or triggering compensatory neurotransmitter release that converges on *trp‐1*‐dependent pathways. These mechanisms highlight the unique role of *trp‐1* in mediating MDMA‐induced behaviors (Figure 2).

Moreover, our findings indicate that these abnormal behaviors in *C. elegans* are reversible. Specifically, nematodes exposed to 10 mM ATS for 10 min and subsequently transferred to a drug‐free vehicle solution, exhibited a gradual return to baseline behavior within 10 min (Figure S9, Supporting Information). This reversibility aligns with existing literature and provides further evidence of the involvement of these pathways in ATS toxicity, suggesting that the behavioral changes induced by ATS are not permanent but depend on continuous drug exposure. Interestingly, our observations suggest a notable trend: ATS that induce a faster SWIP phenotype appear to be associated with a slower recovery time upon drug removal (Figure S9, Supporting Information). This implies that the rapidity of SWIP induction might be indicative of a more profound or sustained disruption of the neural circuits governing locomotion.

The SWIP assay has been suggested as a valuable tool for ATS chiral discrimination. Previous studies have demonstrated that *S*‐(+)‐enantiomers in AMPH and METH exhibit stronger stimulant effects compared to their *R*‐(‐)‐enantiomers.^[^ ^40^ ^]^ Our findings corroborate these conclusions, as we observed that *S*‐(+)‐enantiomers in AMPH‐ and METH‐ induced more potent acute responses in *C. elegans*. The stronger effects of *S*‐(+)‐AMPH and *S*‐(+)‐METH can be attributed to a combination of factors, including enhanced monoamine release and uptake inhibition, and superior receptor binding affinity.^[^ ^40^, ^41^, ^42^, ^43^ ^]^ In rodents, *S*‐(+)‐AMPH is more potent than *R*‐(‐)‐AMPH in stimulating dopamine release,^[^ ^44^, ^45^ ^]^ while in vitro studies show that *S*‐(+)‐METH is more potent than *R*‐(‐)‐METH in dopamine, norepinephrine, and 5‐HT release and uptake inhibition.^[^ ^45^ ^]^ Furthermore, *S*‐(+)‐enantiomers demonstrate greater affinity and selectivity for key drug targets, including DAT, SERT, and trace amine‐associated receptor 1 (TAAR1),^[^ ^46^, ^47^, ^48^ ^]^ resulting in more effective inhibition of neurotransmitter reuptake and potent modulation of dopaminergic neurotransmission. These factors collectively contribute to the enhanced physiological and behavioral effects observed with *S*‐(+)‐enantiomers.

It should be noted that our SAR analysis in *C. elegans* revealed trends that are consistent with existing data from mammalian models. Previous studies demonstrated that **3‐MA** is slightly more potent than **2‐MA** and **4‐MA** as a sympathomimetic agent,^[^ ^49^ ^]^ and both **2‐MA** and **3‐MA** are more effective than **4‐MA** in stimulating locomotor activity in mice.^[^ ^50^, ^51^ ^]^ These findings are consistent with our observations in *C. elegans*, where meta‐substituted AMPH derivatives (e.g., **3‐MA**) exhibited more pronounced effects compared to ortho‐ and para‐substituted analogs, suggesting *C. elegans* as a predictive model for identifying promising AMPH derivatives. Furthermore, in the case of halogen‐substituted AMPH derivatives, the pattern observed in our *C. elegans* experiments closely mirrors those seen in mammalian models. For example, **4‐CA** enhanced locomotor activity and SERT selectivity in mice, contrasting with AMPH's DAT preference.^[^ ^52^ ^]^ Taken together, this cross‐species consistency strengthens the translational relevance of our findings.

Overall, our study underscores the effectiveness of the SWIP assay in assessing the activity of ATS, providing valuable insights into the SAR of these compounds. Although *C. elegans* serves as a valuable model for investigating ATS‐induced behaviors, its applicability in translating research findings to mammals may be restricted due to several significant differences. For instance, humans have 14 serotonin receptors,^[^ ^53^, ^54^ ^]^ whereas *C. elegans* has only 6.^[^ ^33^, ^55^, ^56^, ^57^ ^]^ Moreover, there is a functional disparity in monoamine receptors; for example, *C. elegans* have unique amine‐gated chloride channels LGC–53 and LGC–55. In addition, *C. elegans* does not have the complex nervous system and blood – brain barrier that are characteristic of mammals. Its relatively simple metabolic pathways might also change the bioavailability and activity of drugs. These distinctions emphasize the necessity of being cautious when generalizing the results obtained from *C. elegans* to mammalian systems.

The rapid turnover and structural diversity of NPS present considerable challenges for traditional drug screening methods. Conventional approaches, such as mammalian models or in vitro systems, often struggle to keep up with the fast turnover rates of NPS, hindering the ability to assess their full pharmacological profiles. Additionally, the broad structural diversity of NPS complicates the identification of consistent biochemical targets, making it difficult to develop standardized screening protocols.^[^ ^3^, ^5^, ^58^, ^59^, ^60^, ^61^ ^]^ The *C. elegans* model effectively addresses these challenges by offering a high‐throughput in vivo platform that can rapidly screen large numbers of compounds, including those with diverse chemical structures. Furthermore, *C. elegans* mutants enable targeted exploration of receptor‐specific interactions, and its well‐characterized nervous system allows for detailed investigation of NPS effects on key neurobiological pathways, such as dopaminergic and serotonergic signaling. The use of neurotransmitter probes in transparent *C. elegans* holds promise for real‐time, in vivo mechanistic studies.^[^ ^62^, ^63^, ^64^ ^]^ By observing phenotype and behavioral changes in response to these substances, this model provides valuable insights into their acute effects, overcoming limitations associated with more traditional models. Ultimately, *C. elegans* serves as a powerful tool for studying the complex pharmacology of NPS in a controlled, reproducible, and scalable manner, helping to accelerate the development of evidence‐based early warning systems and bridging critical gaps between the emergence of NPS and timely public health responses.

In conclusion, this study highlights the significant utility of *C. elegans* as a model organism for evaluating the activity and mechanisms of ATS compounds. The SWIP assay emerges as a valuable tool for rapid and precise assessment of ATS‐induced behaviors, including acute effects, chiral discrimination, and SAR analysis. Our findings provide critical insights into the mechanisms underlying ATS action. This work not only advances our understanding of ATS but also sets the stage for future research in high‐throughput screening and the development of new therapeutic strategies for managing ATS abuse and its associated health risks.

## 4 Experimental Section

### General Information of ATS

Common reagents and materials used for the synthesis of ATS were purchased from Energy Chemical (Shanghai, China) and Bide Pharmatech Ltd. (China) and used as received without further purification unless otherwise stated. Mass spectrometer (MS) data were acquired in positive ion mode using a Waters e2695‐QDa detector with an electrospray ionization (ESI) source. Nuclear magnetic resonance (NMR) spectra of synthesized compounds were acquired on a Bruker AV‐300 spectrometer (300 MHz ^1^H, 75 MHz ^13^C). Chemical shifts (δ) are expressed in ppm downfield from tetramethylsilane (TMS) using non‐deuterated solvent present in the bulk deuterated solvent (CDCl_3_ and D_2_O). Data are represented as follows: chemical shift, multiplicity (s = singlet, d = doublet, t = triplet, m = multiplet, br = broad), coupling constant in hertz (Hz), and integration.

*S*‐(+)‐METH‐HCl was purchased from the National Institutes for Food and Drug Control. The syntheses of MDA^[^ ^65^ ^]^ were referred to previous report. Amphetamine‐type derivatives were synthesized according to the literature procedure described by Zhang^[^ ^66^ ^]^ and Guerrero.^[^ ^67^ ^]^ The characterization data such as ^1^H NMR, ^13^C NMR, and MS were provided.

### Characterization data for AMPH Derivatives: rac‐1‐Phenylpropan‐2‐amine (**rac‐amphetamine, rac‐AMPH**)


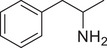


^1^H NMR (300 MHz, CDCl_3_) δ 7.36–7.25 (m, 2H), 7.25–7.05 (m, 3H), 3.16 (dq, *J* = 12.1, 6.2 Hz, 1H), 2.71 (dd, *J* = 13.3, 5.4 Hz, 1H), 2.53 (dd, *J* = 13.3, 8.0 Hz, 1H), 2.02 (s, 3H), 1.12 (d, *J* = 6.3 Hz, 3H). ^13^C NMR (75 MHz, CDCl_3_) δ 139.68, 129.26, 128.40, 126.18, 48.49, 46.63, 23.53. MS (m/z) (ESI): C_9_H_14_N^+^ [M+H]^+^ calcd for: 136.11, found: 136.07.

### *R*‐(‐)‐1‐Phenylpropan‐2‐amine (***R*‐(‐)‐AMPH**)


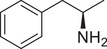


^1^H NMR (300 MHz, D_2_O) δ 7.45–7.04 (m, 5H), 3.47 (h, *J* = 6.8 Hz, 1H), 2.79 (d, *J* = 7.2 Hz, 2H), 1.16 (d, *J* = 6.6 Hz, 3H). ^113^C NMR (75 MHz, D_2_O) δ 136.01, 129.36, 128.95, 127.34, 49.07, 39.99, 17.46. MS (ESI) m/z calcd for C_9_H_14_N^+^ [M +H]^+^ 136.11, found 136.16.

### *S*‐(+)‐1‐Phenylpropan‐2‐amine (***S*‐(+)‐AMPH**)


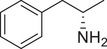


^1^H NMR (300 MHz, CDCl_3_) δ 7.39–7.27 (m, 2H), 7.27–7.10 (m, 3H), 3.18 (dq, *J* = 12.5, 6.3 Hz, 1H), 2.73 (dd, *J* = 13.2, 5.3 Hz, 1H), 2.53 (dd, *J* = 13.2, 8.1 Hz, 1H), 1.17 (br s, 2H), 1.14 (d, *J* = 6.3 Hz, 3H). ^13^C NMR (75 MHz, CDCl_3_) δ 139.76, 129.27, 128.41, 126.18, 48.52, 46.74, 23.64. MS (ESI) m/z calcd for C_9_H_14_N^+^ [M +H]^+^ 136.11, found 136.14.

### *rac*‐N‐methyl‐1‐Phenylpropan‐2‐amine (***rac*‐methamphetamine, *rac*‐METH**)


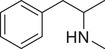


^1^H NMR (300 MHz, CDCl_3_) δ 7.36–7.26 (m, 2H), 7.26–7.13 (m, 3H), 2.89 – 2.73 (m, 2H), 2.64 (dd, *J* = 12.9, 6.2 Hz, 1H), 2.42 (s, 3H), 2.24 (br s, 1H), 1.08 (d, *J* = 6.0 Hz, 3H). ^13^C NMR (75 MHz, CDCl_3_) δ 139.27, 129.30, 128.43, 126.23, 56.40, 43.23, 33.80, 19.46. MS (ESI) m/z calcd for C_10_H_16_N^+^ [M +H]^+^ 150.13, found 150.22.

### *R*‐(‐)‐N‐methyl‐1‐Phenylpropan‐2‐amine (***R*‐(‐)‐METH**)


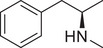


^1^H NMR (300 MHz, CDCl_3_) δ 7.36–7.27 (m, 2H), 7.27–7.16 (m, 3H), 2.89–2.76 (m, 1H), 2.71 (d, *J* = 6.9 Hz, 1H), 2.63 (dd, *J* = 13.0, 6.2 Hz, 1H), 2.41 (s, 3H), 1.53 (s, 1H), 1.08 (d, *J* = 6.1 Hz, 3H). ^13^C NMR (75 MHz, CDCl_3_) δ 139.47, 129.32, 128.42, 126.19, 56.38, 43.46, 34.04, 19.72. MS (ESI) m/z calcd for C_10_H_16_N^+^ [M +H]^+^ 150.13, found 150.21.

### *rac*‐1‐(Benzo[d][1,3]dioxol‐5‐yl)propan‐2‐amine (***rac*‐MDA**)


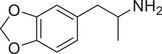


^1^H NMR (300 MHz, CDCl_3_) δ 6.76–6.52 (m, 3H), 5.88 (s, 2H), 3.19–2.97 (m, 1H), 2.59 (dd, *J* = 13.4, 5.5 Hz, 1H), 2.43 (dd, *J* = 13.4, 7.9 Hz, 1H), 1.08 (d, *J* = 6.3 Hz, 3H). ^13^C NMR (75 MHz, CDCl_3_) δ 147.61, 145.96, 133.13, 122.12, 120.18, 109.49, 108.18, 107.84, 100.82, 48.64, 46.12, 45.78, 22.99. MS (m/z) (ESI): C_10_H_14_NO_2_^+^ [M+H]^+^ calcd for: 180.10, found: 180.23.

### *R*‐(‐)‐1‐(Benzo[d][1,3]dioxol‐5‐yl)propan‐2‐amine (***R*‐(‐)‐MDA**)


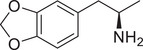


^1^H NMR (300 MHz, CDCl_3_) δ 6.92–6.46 (m, 3H), 5.92 (s, 2H), 3.10 (h, *J* = 6.1 Hz, 1H), 2.62 (dd, *J* = 13.4, 5.4 Hz, 1H), 2.44 (dd, *J* = 13.4, 8.0 Hz, 1H), 1.79 (br s, 2H), 1.10 (d, *J* = 6.3 Hz, 3H). ^13^C NMR (75 MHz, CDCl_3_) δ 147.60, 145.93, 133.35, 122.09, 109.49, 108.16, 100.80, 48.56, 46.12, 23.28. MS (m/z) (ESI): C_10_H_14_NO_2_^+^ [M+H]^+^ calcd for: 180.10, found: 180.23.

### *S*‐(+)‐1‐(Benzo[d][1,3]dioxol‐5‐yl)propan‐2‐amine (***S*‐(+)‐MDA**)


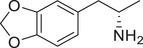


^1^H NMR (300 MHz, CDCl_3_) δ 6.84–6.51 (m, 3H), 5.93 (s, 2H), 3.10 (dq, *J* = 12.5, 6.2 Hz, 1H), 2.63 (dd, *J* = 13.4, 5.2 Hz, 1H), 2.42 (dd, *J* = 13.4, 8.1 Hz, 1H), 1.47 (s, 3H), 1.11 (d, *J* = 6.3 Hz, 3H). ^13^C NMR (75 MHz, CDCl_3_) δ 147.59, 145.91, 133.47, 122.09, 109.49, 108.16, 100.80, 48.56, 46.30, 23.46. MS (m/z) (ESI): C_10_H_14_NO_2_^+^ [M+H]^+^ calcd for: 180.10, found: 180.24.

### *rac*‐1‐(Benzo[d][1,3]dioxol‐5‐yl)‐N‐methylpropan‐2‐amine‐HCl (***rac*‐MDMA‐HCl**)


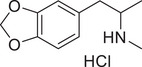


^1^H NMR (300 MHz, D_2_O) δ 6.84–6.54 (m, 3H), 5.82 (s, 2H), 3.35 (dt, *J* = 13.1, 6.5 Hz, 1H), 2.84 (dd, *J* = 13.9, 6.2 Hz, 1H), 2.76–2.66 (m, 1H), 2.57 (s, 3H), 1.14 (d, *J* = 6.6 Hz, 3H). ^13^C NMR (75 MHz, D_2_O) δ 147.38, 146.20, 129.31, 122.71, 109.58, 108.62, 101.04, 56.39, 38.39, 29.84, 14.71. MS (m/z) (ESI): C_11_H_16_NO_2_^+^ [M+H]^+^ calcd for: 194.12, found: 194.27.

### *R*‐(‐)‐1‐(Benzo[d][1,3]dioxol‐5‐yl)‐N‐methylpropan‐2‐amine‐HCl (***R*‐(‐)‐MDMA‐HCl**)


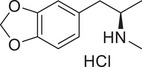


^1^H NMR (500 MHz, D_2_O) δ 6.79 (d, *J* = 7.9 Hz, 1H), 6.74 (s, 1H), 6.69 (d, *J* = 7.9 Hz, 1H), 5.87 (s, 2H), 3.38 (h, *J* = 6.8 Hz, 1H), 2.87 (dd, *J* = 14.0, 6.5 Hz, 1H), 2.74 (dd, *J* = 14.1, 7.7 Hz, 1H), 2.60 (s, 3H), 1.17 (d, *J* = 6.6 Hz, 3H). ^13^C NMR (126 MHz, D_2_O) δ 147.46, 146.29, 129.37, 122.75, 109.64, 108.68, 101.09, 56.43, 38.42, 29.86, 14.74. MS (m/z) (ESI): C_11_H_16_NO_2_^+^ [M+H]^+^ calcd for: 194.12, found: 194.26.

### *S*‐(+)‐1‐(Benzo[d][1,3]dioxol‐5‐yl)‐N‐methylpropan‐2‐amine‐HCl (***S*‐(+)‐MDMA‐HCl**)


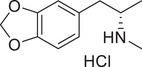


^1^H NMR (500 MHz, D_2_O) δ 6.78 (d, *J* = 7.9 Hz, 1H), 6.74 (s, 1H), 6.68 (d, *J* = 7.9 Hz, 1H), 5.86 (s, 2H), 3.38 (h, *J* = 6.4 Hz, 1H), 2.87 (dd, *J* = 13.9, 6.4 Hz, 1H), 2.73 (dd, J = 13.9, 7.7 Hz, 1H), 2.59 (s, 3H), 1.17 (d, *J* = 6.5 Hz, 3H). ^13^C NMR (126 MHz, D_2_O) δ 147.45, 146.27, 129.37, 122.74, 109.63, 108.68, 101.08, 56.42, 38.42, 29.86, 14.74. MS (m/z) (ESI): C_11_H_16_NO_2_^+^ [M+H]^+^ calcd for: 194.12, found: 194.26.

### 1‐(2‐Methoxyphenyl)propan‐2‐amine (**2‐OMA**)


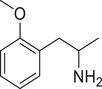


^1^H NMR (300 MHz, CDCl_3_) δ 7.27 – 7.06 (m, 2H), 6.98 – 6.79 (m, 2H), 3.21 (dq, *J* = 12.6, 6.3 Hz, 1H), 2.75 (dd, *J* = 13.0, 5.4 Hz, 1H), 2.55 (dd, *J* = 13.0, 7.9 Hz, 1H), 1.65 (br s, 2H), 1.12 (d, *J* = 6.3 Hz, 3H). ^13^C NMR (75 MHz, CDCl_3_) δ 157.68, 131.09, 128.06, 127.50, 120.32, 110.32, 55.21, 47.06, 41.11, 23.58. MS (m/z) (ESI): C_10_H_16_NO^+^ [M+H]^+^ calcd for: 166.12, found: 166.22.

### 1‐(*o*‐Tolyl)propan‐2‐amine (**2‐MA**)


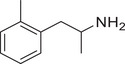


^1^H NMR (300 MHz, CDCl_3_) δ 7.16 (s, 4H), 3.20 (dq, *J* = 12.3, 6.2 Hz, 1H), 2.75 (dd, *J* = 13.5, 5.5 Hz, 1H), 2.57 (dd, *J* = 13.5, 8.1 Hz, 1H), 2.34 (s, 3H), 1.48 (br s, 2H), 1.16 (d, *J* = 6.3 Hz, 3H). ^13^C NMR (75 MHz, CDCl_3_) δ 137.98, 136.39, 130.39, 130.04, 126.31, 125.86, 47.35, 43.90, 23.71, 19.65. MS (m/z) (ESI): C_10_H_16_N^+^ [M+H]^+^ calcd for: 150.13, found: 150.20.

### 1‐(2‐Bromophenyl)propan‐2‐amine (**2‐BA**)


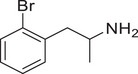


^1^H NMR (300 MHz, CDCl_3_ δ 7.52 (d, *J* = 7.5 Hz, 1H), 7.30–6.96 (m, 3H), 3.35–3.12 (m, 1H), 2.92–2.52 (m, 2H), 1.41 (s, 2H), 1.12 (d, *J* = 6.3 Hz, 3H). ^13^C NMR (75 MHz, CDCl_3_) δ 139.18, 133.01, 131.55, 128.01, 127.37, 124.98, 47.11, 46.61, 23.55. MS (m/z) (ESI): C_9_H_13_BrN^+^ [M+H]^+^ calcd for: 214.02, found: 214.17.

### 1‐(2‐Chlorophenyl)propan‐2‐amine (**2‐CA**)


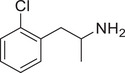


^1^H NMR (300 MHz, CDCl_3_) δ 7.47–7.06 (m, 4H), 3.27 (dt, *J* = 7.7, 5.9 Hz, 1H), 2.94–2.55 (m, 2H), 1.33 (s, 2H), 1.14 (d, *J* = 6.3 Hz, 3H). ^13^C NMR (75 MHz, CDCl_3_) δ 137.44, 134.33, 131.50, 129.62, 127.68, 126.66, 47.02, 44.22, 23.59. MS (m/z) (ESI): C_9_H_13_ClN^+^ [M+H]^+^ calcd for: 170.07, found: 170.05.

### 1‐(2‐Fluorophenyl)propan‐2‐amine (**2‐FA**)


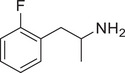


^1^H NMR (300 MHz, CDCl_3_) δ 7.39–6.89 (m, 4H), 3.19 (q, *J* = 6.5 Hz, 1H), 2.87–2.39 (m, 2H), 1.38 (s, 2H), 1.12 (d, *J* = 6.3 Hz, 3H). ^13^C NMR (101 MHz, CDCl_3_) δ 161.39 (d, *J* = 244.7 Hz), 131.70 (d, *J* = 5.1 Hz), 128.04 (d, *J* = 8.2 Hz), 126.59 (d, *J* = 15.9 Hz), 124.00 (d, *J* = 3.6 Hz), 115.39 (d, *J* = 22.5 Hz), 47.60 (d, *J* = 1.2 Hz)., 39.88, 23.56. MS (m/z) (ESI): C_9_H_13_FN^+^ [M+H]^+^ calcd for: 154.10, found: 154.08.

### 1‐(2‐(Trifluoromethyl)phenyl)propan ‐2‐amine (**2‐TFA**)


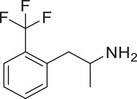


^1^H NMR (300 MHz, CDCl_3_) δ 7.66 (d, *J* = 7.9 Hz, 1H), 7.50 (t, *J* = 7.5 Hz, 1H), 7.35 (dd, *J* = 18.3, 7.7 Hz, 2H), 3.23 (h, *J* = 6.1 Hz, 1H), 2.92 (dd, *J* = 13.8, 4.9 Hz, 1H), 2.72 (dd, *J* = 13.6, 7.8 Hz, 1H), 1.46 (br s, 2H), 1.17 (d, *J* = 6.3 Hz, 3H). ^13^C NMR (75 MHz, CDCl_3_) δ 138.49, 131.71, 131.59, 126.32, 126.23 (d, *J* = 5.8 Hz), 125.95 (d, *J* = 447.9 Hz), 48.21, 43.16, 23.80. MS (m/z) (ESI): C_10_H_13_F_3_N^+^ [M+H]^+^ calcd for: 204.10, found:204.07.

### 1‐(2‐Methoxyphenyl)propan‐2‐amine (**3‐OMA**)


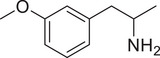


^1^H NMR (300 MHz, CDCl_3_) δ 7.23 (t, *J* = 7.8 Hz, 1H), 6.87–6.68 (m, 3H), 3.81 (s, 3H), 3.18 (dq, *J* = 12.5, 6.3 Hz, 1H), 2.71 (dd, *J* = 13.2, 5.2 Hz, 1H), 2.50 (dd, *J* = 13.2, 8.2 Hz, 1H), 1.75 (br s, 2H), 1.14 (d, *J* = 6.3 Hz, 3H). ^13^C NMR (75 MHz, CDCl_3_) δ 159.63, 141.27, 129.39, 121.63, 114.93, 111.48, 55.14, 48.42, 46.63, 23.52. MS (m/z) (ESI): C_10_H_16_NO^+^ [M+H]^+^ calcd for: 166.12, found: 166.16.

### 1‐(*m*‐Tolyl)propan‐2‐amine (**3‐MA**)


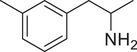


^1^H NMR (300 MHz, CDCl_3_) δ 7.19 (t, *J* = 7.4 Hz, 1H), 7.00 (t, *J* = 10.3 Hz, 3H), 3.15 (dq, *J* = 12.6, 6.3 Hz, 1H), 2.68 (dd, *J* = 13.2, 5.3 Hz, 1H), 2.47 (dd, *J* = 13.2, 8.1 Hz, 1H), 2.39–2.23 (m, 3H), 1.61 (br s, 2H), 1.12 (d, *J* = 6.3 Hz, 3H). ^13^C NMR (101 MHz, CDCl_3_) δ 139.42, 137.98, 130.06, 128.32, 126.99, 126.26, 48.48, 46.34, 23.32, 21.39. MS (m/z) (ESI): C_10_H_16_N^+^ [M+H]^+^ calcd for: 150.13, found: 150.20.

### 1‐(3‐Bromophenyl)propan‐2‐amine (**3‐BA**)


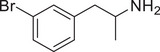


^1^H NMR (300 MHz, CDCl_3_) δ 7.46–6.98 (m, 4H), 3.16 (dt, *J* = 8.0, 6.0 Hz, 1H), 2.79–2.39 (m, 2H), 1.41 (s, 3H), 1.12 (d, *J* = 6.3 Hz, 3H). ^13^C NMR (75 MHz, CDCl_3_) δ 142.09, 132.20, 129.96, 129.34, 127.92, 122.49, 48.35, 46.22, 23.54. MS (m/z) (ESI): C_9_H_13_BrN^+^ [M+H]^+^ calcd for: 214.02, found: 214.17.

### 1‐(3‐Chlorophenyl)propan‐2‐amine (**3‐CA**)


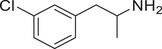


^1^H NMR (300 MHz, CDCl_3_) δ 7.20 (t, *J* = 6.6 Hz, 4H), 7.06 (d, *J* = 6.6 Hz, 1H), 3.16 (dd, *J* = 13.4, 6.3 Hz, 1H), 2.67 (dd, *J* = 13.3, 5.3 Hz, 1H), 2.50 (dd, *J* = 13.3, 8.0 Hz, 1H), 1.47 (s, 3H), 1.11 (d, *J* = 6.3 Hz, 3H). ^13^C NMR (75 MHz, CDCl_3_) δ 141.69, 134.16, 129.66, 129.28, 127.45, 126.43, 48.33, 46.15, 23.45. MS (m/z) (ESI): C_9_H_13_ClN^+^ [M+H]^+^ calcd for: 170.07, found: 170.19.

### 1‐(3‐Fluorophenyl)propan‐2‐amine (**3‐FA**)


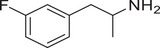


^1^H NMR (300 MHz, CDCl_3_) δ 7.38–7.13 (m, 1H), 6.93 (dd, *J* = 19.4, 8.5 Hz, 3H), 3.31–3.06 (m, 1H), 2.78–2.42 (m, 2H), 1.40 (s, 2H), 1.12 (d, *J* = 6.3 Hz, 3H). ^13^C NMR (75 MHz, CDCl_3_) δ 162.95 (d, *J* = 245.5 Hz), 142.36 (d, *J* = 7.1 Hz), 129.88 (d, *J* = 8.3 Hz), 124.99 (d, *J* = 2.8 Hz), 116.07 (d, *J* = 20.8 Hz), 113.17 (d, *J* = 21.0 Hz), 48.42, 46.40 (d, *J* = 1.7 Hz), 23.61. MS (m/z) (ESI): C_9_H_13_FN^+^ [M+H]^+^ calcd for: 154.10, found: 154.21.

### 1‐(3‐(Trifluoromethyl)phenyl)propan‐2‐amine (**3‐TFA**)


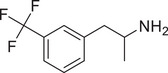


^1^H NMR (300 MHz, CDCl_3_) δ 7.43 (dq, *J* = 15.9, 7.9 Hz, 4H), 3.20 (dq, *J* = 12.5, 6.2 Hz, 1H), 2.76 (dd, *J* = 13.3, 5.5 Hz, 1H), 2.61 (dd, *J* = 13.3, 7.9 Hz, 1H), 1.68 (br s, 2H), 1.13 (d, *J* = 6.3 Hz, 3H). ^13^C NMR (75 MHz, CDCl_3_) δ ^13^C NMR (75 MHz, CDCl_3_) δ 140.55, 132.66, 128.81, 128.24 (d, *J* = 338.2 Hz), 126.65 (d, *J* = 642.2 Hz), 125.84 (q, *J* = 3.8 Hz), 123.12 (q, *J* = 3.8 Hz), 48.35, 46.21, 23.40. MS (m/z) (ESI): C_10_H_13_F_3_N^+^ [M+H]^+^ calcd for: 204.10, found:204.25.

### 1‐(4‐Methoxyphenyl)propan‐2‐amine (**4‐OMA**)


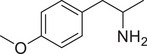


^1^H NMR (300 MHz, CDCl_3_) δ 7.12 (d, *J* = 8.6 Hz, 2H), 6.86 (d, *J* = 8.7 Hz, 2H), 3.81 (s, 3H), 3.14 (dq, *J* = 12.6, 6.3 Hz, 1H), 2.68 (dd, *J* = 13.4, 5.4 Hz, 1H), 2.48 (dd, *J* = 13.4, 8.0 Hz, 1H), 1.67 (br s, 2H), 1.13 (d, *J* = 6.3 Hz, 3H). ^13^C NMR (75 MHz, CDCl_3_) δ 158.06, 131.61, 130.17, 113.81, 55.26, 48.61, 45.54, 23.33. MS (m/z) (ESI): C_10_H_16_NO^+^ [M+H]^+^ calcd for: 166.12, found: 166.20.

### 1‐(*p*‐Tolyl)propan‐2‐amine (**4‐MA**)


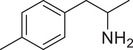


^1^H NMR (300 MHz, CDCl_3_) δ 7.19 – 7.00 (m, 4H), 3.16 (dq, *J* = 12.6, 6.3 Hz, 1H), 2.70 (dd, *J* = 13.3, 5.3 Hz, 1H), 2.49 (dd, *J* = 13.3, 8.1 Hz, 1H), 2.34 (s, 3H), 1.43 (br s, 2H), 1.13 (d, *J* = 6.3 Hz, 3H). ^13^C NMR (75 MHz, CDCl_3_) δ 136.52, 135.68, 129.14, 129.10, 48.56, 46.13, 23.46, 21.04. MS (m/z) (ESI): C_10_H_16_N^+^ [M+H]^+^ calcd for: 150.13, found: 150.09.

### 1‐(4‐Bromophenyl)propan‐2‐amine (**4‐BA**)


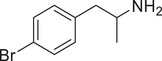


^1^H NMR (300 MHz, Chloroform‐*d*) δ 7.41 (d, *J* = 8.3 Hz, 2H), 7.05 (d, *J* = 8.3 Hz, 2H), 3.23–3.02 (m, 1H), 2.65 (dd, *J* = 13.3, 5.4 Hz, 1H), 2.48 (dd, *J* = 13.3, 8.0 Hz, 1H), 1.60 (s br, 3H), 1.10 (d, *J* = 6.3 Hz, 3H). ^13^C NMR (75 MHz, CDCl_3_) δ 138.47, 131.46, 131.46, 130.99, 130.99, 120.07, 48.36, 45.69, 23.28. MS (m/z) (ESI): C_9_H_13_BrN^+^ [M+H]^+^ calcd for: 214.02, found: 214.18.

### 1‐(4‐Chlorophenyl)propan‐2‐amine (**4‐CA**)


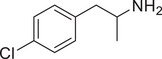


^1^H NMR (300 MHz, CDCl_3_) δ 7.18–7.06 (m, 2H), 7.04–6.86 (m, 2H), 3.12 (dq, *J* = 12.5, 6.3 Hz, 1H), 2.66 (dd, *J* = 13.4, 5.4 Hz, 1H), 2.48 (dd, *J* = 13.4, 8.0 Hz, 1H), 1.40 (br s, 2H), 1.09 (d, *J* = 6.3 Hz, 3H). ^13^C NMR (75 MHz, CDCl_3_) δ 138.11, 131.98, 130.57, 128.50, 48.41, 45.86, 23.49. MS (m/z) (ESI): C_9_H_13_ClN^+^ [M+H]^+^ calcd for: 170.07, found: 170.05. MS (m/z) (ESI): C_9_H_13_ClN^+^ [M+H]^+^ calcd for: 170.07, found: 170.19.

### 1‐(4‐Fluorophenyl)propan‐2‐amine (**4‐FA**)


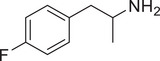


^1^H NMR (300 MHz, CDCl_3_) δ 7.10 (dd, *J* = 8.5, 5.6 Hz, 2H), 6.94 (t, *J* = 8.7 Hz, 2H), 3.17–3.00 (m, 1H), 2.63 (dd, *J* = 13.4, 5.4 Hz, 1H), 2.46 (dd, *J* = 13.4, 8.0 Hz, 1H), 1.38 (s, 2H), 1.07 (d, *J* = 6.3 Hz, 3H). ^13^C NMR (75 MHz, CDCl_3_) δ161.48 (d, *J* = 243.8 Hz), 135.30 (d, *J* = 3.3 Hz), 130.56 (d, *J* = 7.8 Hz), 115.12 (d, *J* = 21.1 Hz), 48.49, 48.48, 45.68. MS (m/z) (ESI): C_9_H_13_FN^+^ [M+H]^+^ calcd for: 154.10, found: 154.21.

### 1‐(4‐(Trifluoromethyl)phenyl)propan‐2‐amine (**4‐TFA**)


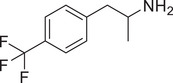


^1^H NMR (500 MHz, CDCl_3_) δ 7.54 (d, *J* = 7.7 Hz, 2H), 7.29 (d, *J* = 7.6 Hz, 2H), 3.37 – 3.03 (m, 1H), 2.74 (dd, *J* = 13.3, 5.5 Hz, 1H), 2.60 (dd, *J* = 13.3, 7.9 Hz, 1H), 1.64 (br s, 2H), 1.12 (d, *J* = 6.2 Hz, 3H). ^13^C NMR (126 MHz, CDCl_3_) δ 143.77, 129.52, 128.55 (d, *J* = 32.5 Hz), 125.28 (q, *J* = 3.8 Hz), 124.30 (d, *J* = 272.3 Hz), 48.32, 46.20, 23.42. MS (m/z) (ESI): C_10_H_13_F_3_N^+^ [M+H]^+^ calcd for: 204.10, found: 204.07.

### 1‐(3,4‐Dimethoxyphenyl)propan‐2‐amine (**3,4‐di‐OMA**)


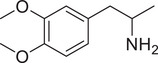


^1^H NMR (300 MHz, CDCl_3_) δ 6.79 (d, *J* = 8.6 Hz, 1H), 6.71 (d, *J* = 6.5 Hz, 2H), 3.84 (d, *J* = 4.0 Hz, 6H), 3.28–3.01 (m, 1H), 2.66 (dd, *J* = 13.4, 5.4 Hz, 1H), 2.48 (dd, *J* = 13.4, 8.0 Hz, 1H), 2.16 (s, 2H), 1.12 (d, *J* = 6.3 Hz, 3H). ^13^C NMR (75 MHz, CDCl_3_) δ 148.73, 147.40, 131.91, 121.11, 112.29, 111.13, 55.82, 55.76, 48.54, 45.60, 23.00. MS (m/z) (ESI): C_11_H_18_NO_2_^+^ [M+H]^+^ calcd for: 196.13, found: 196.26.

### 1‐(3,5‐Dimethoxyphenyl)propan‐2‐amine (**3,5‐di‐OMA**)


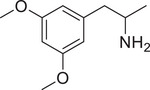


^1^H NMR (300 MHz, CDCl_3_) δ 6.49 (s, 1H), 6.36 (s, 2H), 3.80 (d, *J* = 4.2 Hz, 6H), 3.36 – 3.10 (m, 1H), 2.69 (dd, *J* = 13.2, 5.5 Hz, 1H), 2.52 (dd, *J* = 13.2, 8.0 Hz, 1H), 2.23 (br s, 2H), 1.17 (d, *J* = 6.3 Hz, 3H). ^13^C NMR (75 MHz, CDCl_3_) δ 160.78, 141.66, 107.20, 104.93, 98.20, 55.23, 48.40, 46.29, 22.99. MS (m/z) (ESI): C_11_H_18_NO_2_^+^ [M+H]^+^ calcd for: 196.13, found: 196.25.

### 1‐(2,5‐Dimethoxyphenyl)propan‐2‐amine (**2,5‐di‐OMA**)


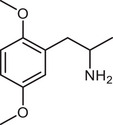


^1^H NMR (300 MHz, CDCl_3_) δ 6.75 (d, *J* = 10.6 Hz, 3H), 3.76 (d, *J* = 1.5 Hz, 6H), 3.20 (h, *J* = 6.3 Hz, 1H), 2.71 (dd, *J* = 13.1, 5.3 Hz, 1H), 2.52 (dd, *J* = 13.0, 7.9 Hz, 1H), 1.68 (s, 2H), 1.12 (d, *J* = 6.5 Hz, 3H). ^13^C NMR (75 MHz, CDCl_3_) δ 153.29, 152.00, 129.26, 117.33, 111.38, 111.26, 55.83, 55.64, 47.14, 41.18, 23.54. MS (m/z) (ESI): C_11_H_18_NO_2_^+^ [M+H]^+^ calcd for: 196.13, found: 196.14.

### *C. elegans* Strain and Culture

*C. elegans* strains were maintained under standard conditions at 20 °C.^[^ ^68^ ^]^ Bristol strain N2 from CGC (Caenorhabditis Genetics Center) was used as the wild type strain. Other strains were also obtained from CGC: LX703 (*dop‐3(vs106)* X); MT13952 (*lgc‐53(n4330)* X); LX636 (*dop‐1(vs101)* X); LX706 (*dop‐2(vs105)* V; *dop‐1(vs100)* X); LX702 (*dop‐2(vs105)* V); RM2702 (*dat‐1(ok157)* III); MT15620 (*cat‐2(n4547)* II); FG58 (*dop‐4(tm1392)* X); LC33 (*bas‐1(tm351)* III); CB1111 (*cat‐1(e1111)* X); MT14984 (*tph‐1(n4622)* II); DA1814 (*ser‐1(ok345)* X); MT9886 (*mod‐1(ok103)* V); DA2100 (*ser‐7(tm1325)* X); RB745 (*ser‐4(ok512)* III); MT8944 (*mod‐5(n822)* I); RB1172 (*acr‐15(ok1214)* V); NC292 (*acr‐5(ok182)* III); TQ225 (*trp‐1(sy690)* III); TQ194 (*trp‐2(sy691)* III); MT14680 (*lgc‐55(n4331)* V); KP4 (*glr‐1(n2461)* III); MT6308 (*eat‐4(ky5)* III); RB1808 (*glr‐2(ok2342)* III).

### Swimming‐Induced Paralysis (SWIP) Assay

In the SWIP assay, late L4 stage *C. elegans* grown on NGM plates with OP50 bacteria were used for time‐dose experiments. For each trial, 8–12 *C. elegans* were placed in a glass spot plate well containing 20 µL of a 200 mm sucrose solution, with or without different concentrations of ATS. All compounds were in their basic form and were dissolved in DMSO to prepare 1 m stock solutions, then diluted to achieve the desired test concentrations with a final DMSO content of 1%. The number of uncoordinated swimming *C. elegans* was recorded each minute to calculate the paralysis rate, with three independent experiments conducted. To determine the primary mechanisms of ATS‐induced SWIP, 10 day 1 adult *C. elegans* were used in each trial, with drug concentrations of 15 mm *S*‐(+)‐METH and 10 mm *rac*‐MDMA. The onset of the first SWIP behavior was recorded, and three independent experiments were performed.

### Reversibility Assays

Approximately 10 *C. elegans* were exposed to a 10 mm ATS solution for 10 min as in the SWIP assay. Subsequently, these *C. elegans* were transferred to a glass spot plate well containing 20 µL of a drug‐free vehicle solution (200 mm sucrose solution with 1% DMSO). Behavioral recovery was monitored by observing the return to coordinated swimming after 10 min. The recovery rate was then documented and quantified, with three independent experiments conducted.

### Crawling‐Induced Paralysis (CIP) Assay

Day‐1 adult *C. elegans* were transferred to 2% agar plates containing either 15 mm METH or 10 mm MDMA. Their locomotion morphology was then observed. *C. elegans* exhibiting head‐straight immobilization were defined as exhibiting crawling‐induced paralysis. The first time point of abnormal movement was recorded. The experiment was performed in triplicate, and each repeat was performed using at least 10 *C. elegans*.

### Statistical Analysis

Statistical analysis was performed using GraphPad Prism 9.1.0 software (GraphPad Software, La Jolla, CA, USA). Data were presented as the mean ± SEM. For SWIP and CIP assays, p values were determined by Student's t‐test to analyze the difference between the two groups. Differences were considered significant at *p* < 0.05.

## Conflict of Interest

The authors declare no conflict of interest.

## Author Contributions

Y.L. conducted the *C. elegans* experiments and wrote the original manuscript. H.L. supervised the project and provided revisions to the manuscript. H.W. synthesized the compounds and contributed to manuscript writing. X.W. proposed the concept, supervised the project, and revised the manuscript.

## Supporting information



Supporting Information

Supplemental Video 1

Supplemental Video 2

Supplemental Video 3

Supplemental Video 4

Supplemental Video 5

Supplemental Video 6

Supplemental Video 7

Supplemental Video 8

## Data Availability

The data that support the findings of this study are available in the supplementary material of this article.
