# Supplementary material for: Utilizing Caenorhabditis Elegans as a Rapid and Precise Model for Assessing Amphetamine‐Type Stimulants: A Novel Approach to Evaluating New Psychoactive Substances Activity and Mechanisms
Source: Adv Sci (Weinh). 2025 Mar 11;12(17):2500808. doi: 10.1002/advs.202500808 (PMC12061310; doi:10.1002/advs.202500808)
Supplement: Supplementary file 1 — Supporting Information [file ADVS-12-2500808-s006.docx]

**Supporting information**

**Utilizing *Caenorhabditis elegans* as a Rapid and Precise Model for Assessing Amphetamine-Type Stimulants: A Novel Approach to Evaluating New Psychoactive Substances Activity and Mechanisms**

Yuanpeng Li^1,2^, Hongyuan Li^1*^, Hongshuang Wang^1*^, Xiaohui Wang^1,2,3*^

^1^Laboratory of Chemical Biology, Changchun Institute of Applied Chemistry, Chinese Academy of Sciences, Changchun, Jilin, 130022, China

^2^School of Applied Chemistry and Engineering, University of Science and Technology of China, Hefei, Anhui, 230026, China

^3^State Key Laboratory of Brain Machine Intelligence, Zhejiang University, Hangzhou, 310027, China

***** Corresponding authors

E-mail: [hongyuan.li@ciac.ac.cn](mailto:hongyuan.li@ciac.ac.cn); [hongshuang.wang@ciac.ac.cn](mailto:hongshuang.wang@ciac.ac.cn); [xiaohui.wang@ciac.ac.cn](mailto:xiaohui.wang@ciac.ac.cn)


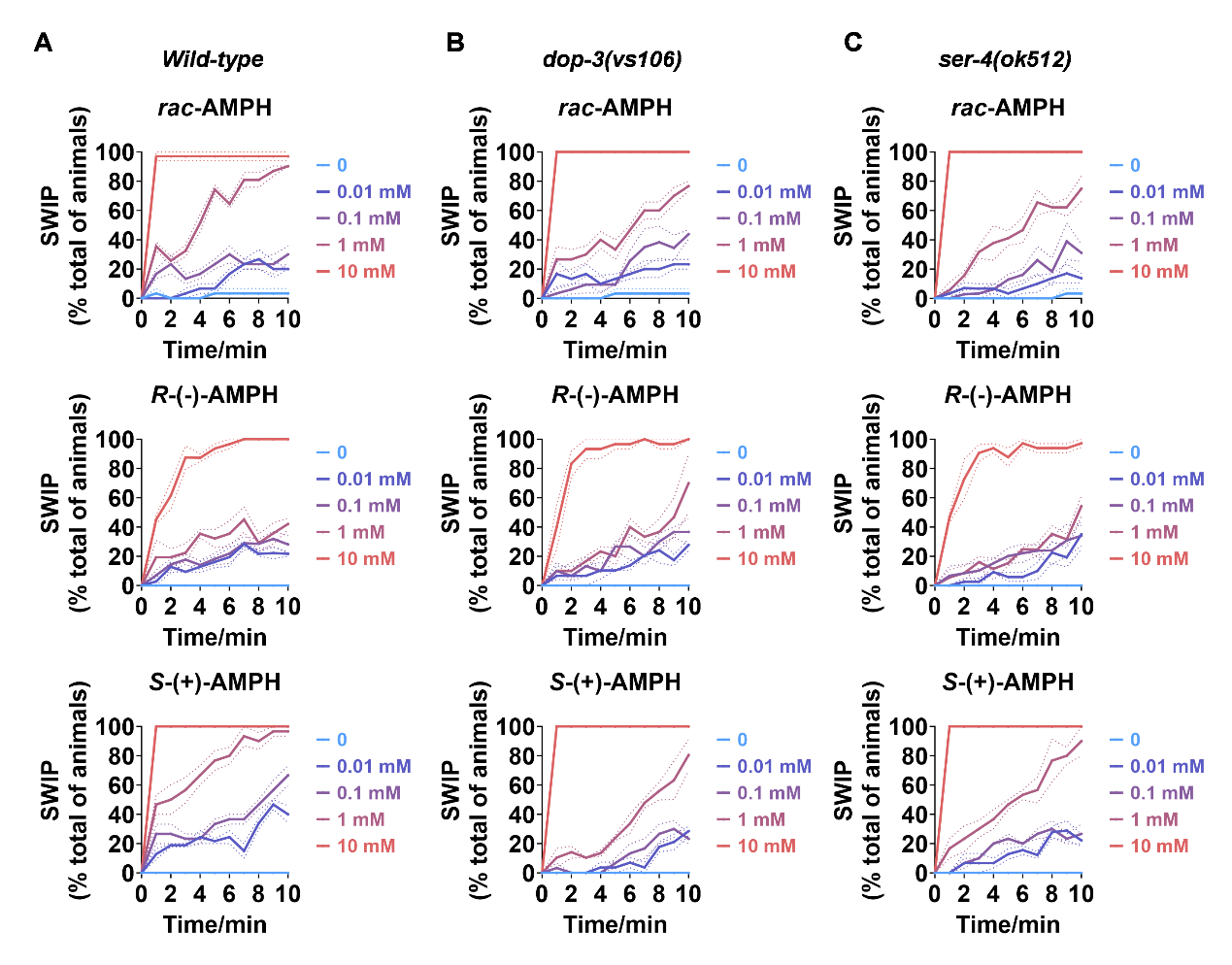


**Figure S1.** Chiral activity of AMPH-induced SWIP behavior in *C. elegans*. (A-C) SWIP behavior induced by various concentrations (0, 0.01, 0.1, 1, and 10 mM) of *rac*-AMPH, *R*-(-)-AMPH, and *S*-(+)-AMPH in: (A) N2, (B) LX703, (C) RB745. Three independent experiments were conducted.

**
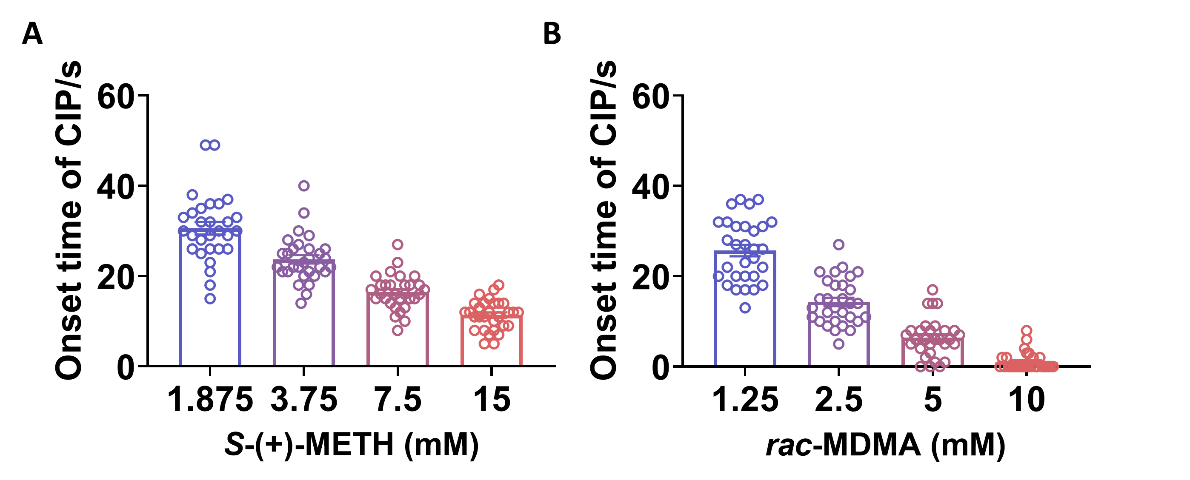
**

**Figure S2.** The onset time of *S-*(+)-METH and *rac*-MDMA caused CIP behaviors in N2. Day-1 adult *C. elegans* grown on NGM plates with OP50 bacteria were used for CIP behavior measurement. The onset time of CIP were recorded after transferring each *C. elegans* to 2% agar plates containing either 15 mM *S-*(+)-METH (A) or 10 mM *rac*-MDMA (B). The experiment was repeated three times, and during each experiment, at least 10 *C. elegans* were used.

**
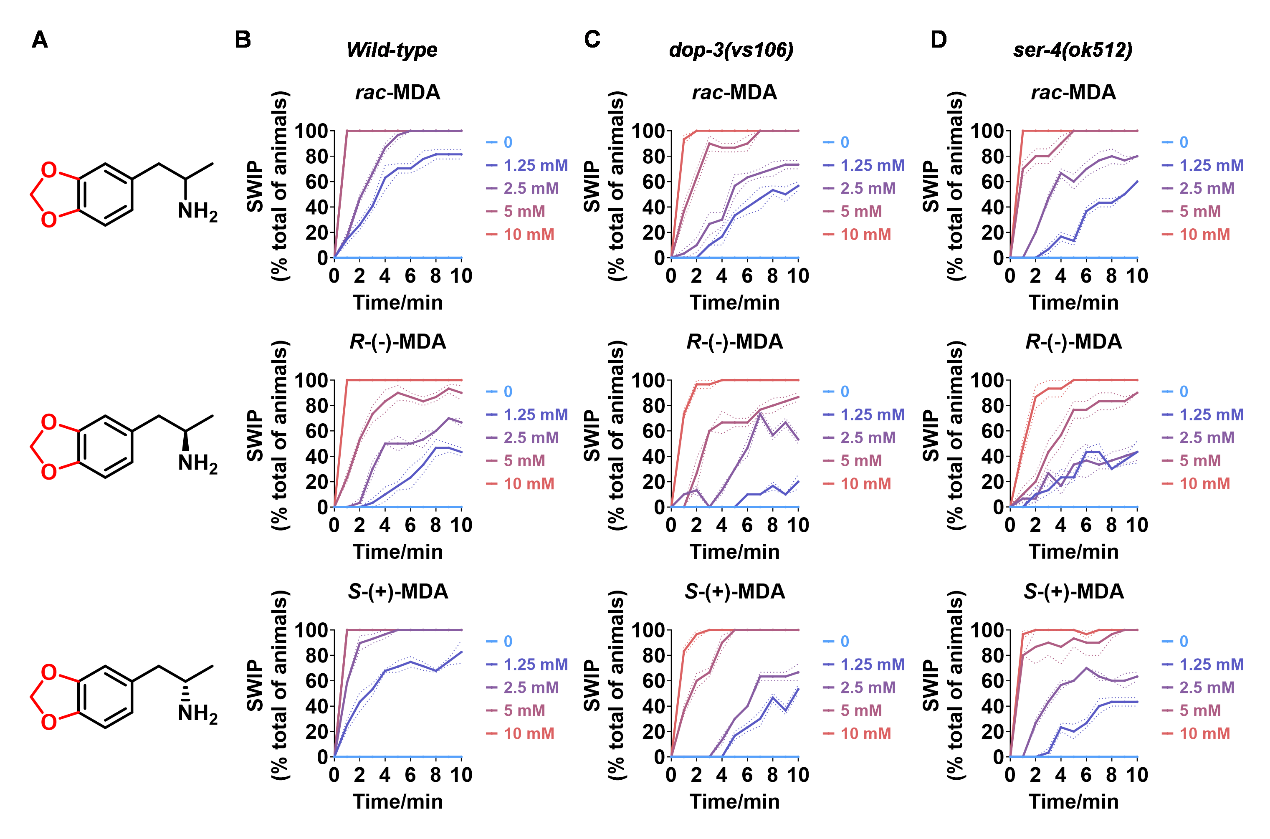
**

**Figure S3.** Chiral activity of MDA-induced SWIP behavior in *C. elegans*. (A) Chemical structures of *rac*-MDA, *R*-(-)-MDA, and *S*-(+)-MDA. (B-D) SWIP behavior induced by various concentrations (0, 1.25, 2.5, 5 and 10 mM) of *rac*-MDA, *R*-(-)-MDA, and *S*-(+)-MDA in: (B) N2, (C) *dop-3* mutants, (D) *ser-4* mutants. Three independent experiments were conducted.

**
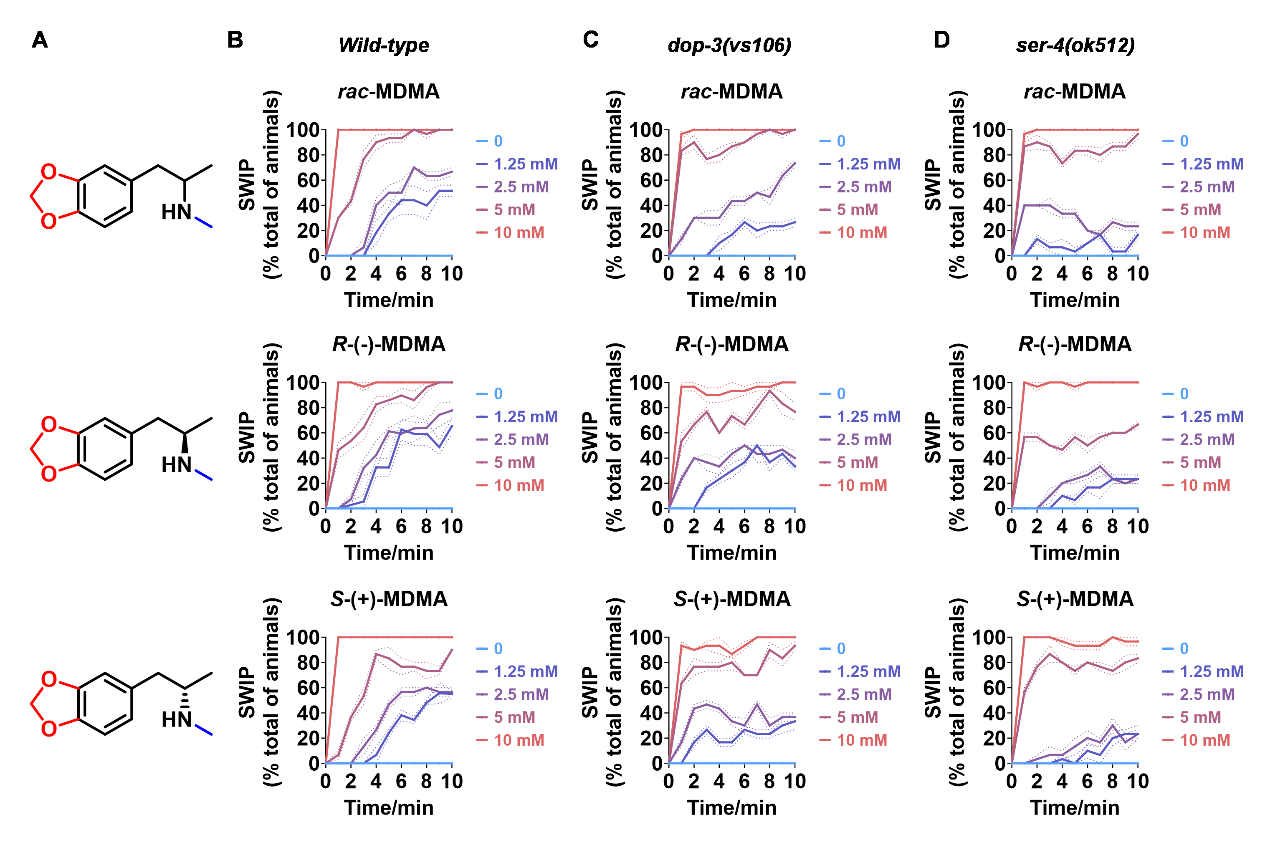
**

**Figure S4.** Chiral activity of MDMA-induced SWIP behavior in *C. elegans*. (A) Chemical structures of *rac*-MDMA, *R*-(-)-MDMA, and *S*-(+)-MDMA. (**B-D)** SWIP behavior induced by various concentrations (0, 1.25, 2.5 5 and 10 mM) of *rac*-MDMA, *R*-(-)-MDMA, and *S*-(+)-MDMA in: (**B)** N2, (**C)** *dop-3* mutants, (**D)** *ser-4* mutants. Three independent experiments were conducted.


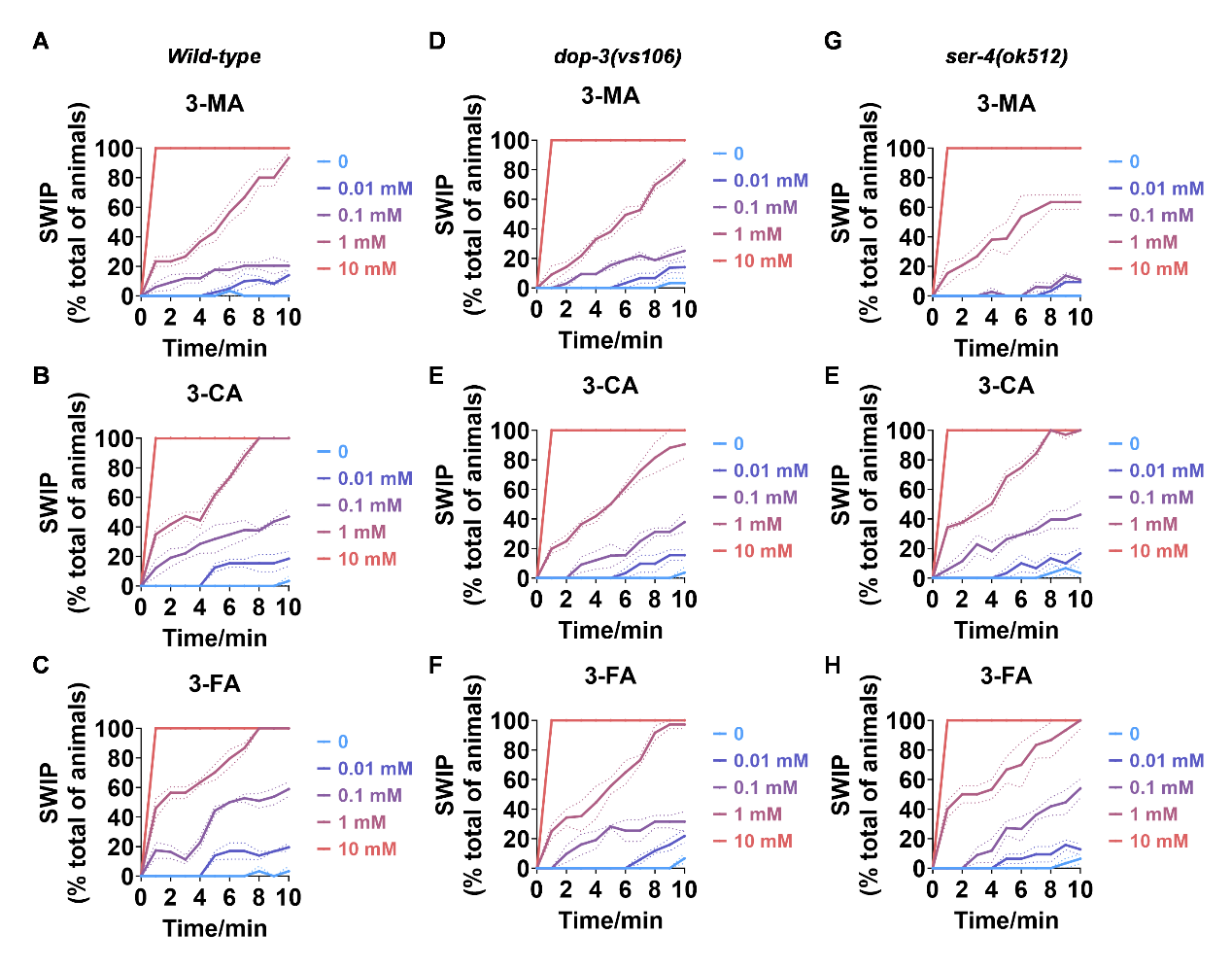


**Figure S5.** Functional comparison of ATS-induced SWIP behavior in *C. elegans*. SWIP behaviors induced by various concentrations (0, 0.01, 0.1, 1, and 10 mM) of 3-MA, 3-CA and 3-FA in N2 (A-C), LX703 (D-F), RB745 (G-H). Three independent experiments were conducted.


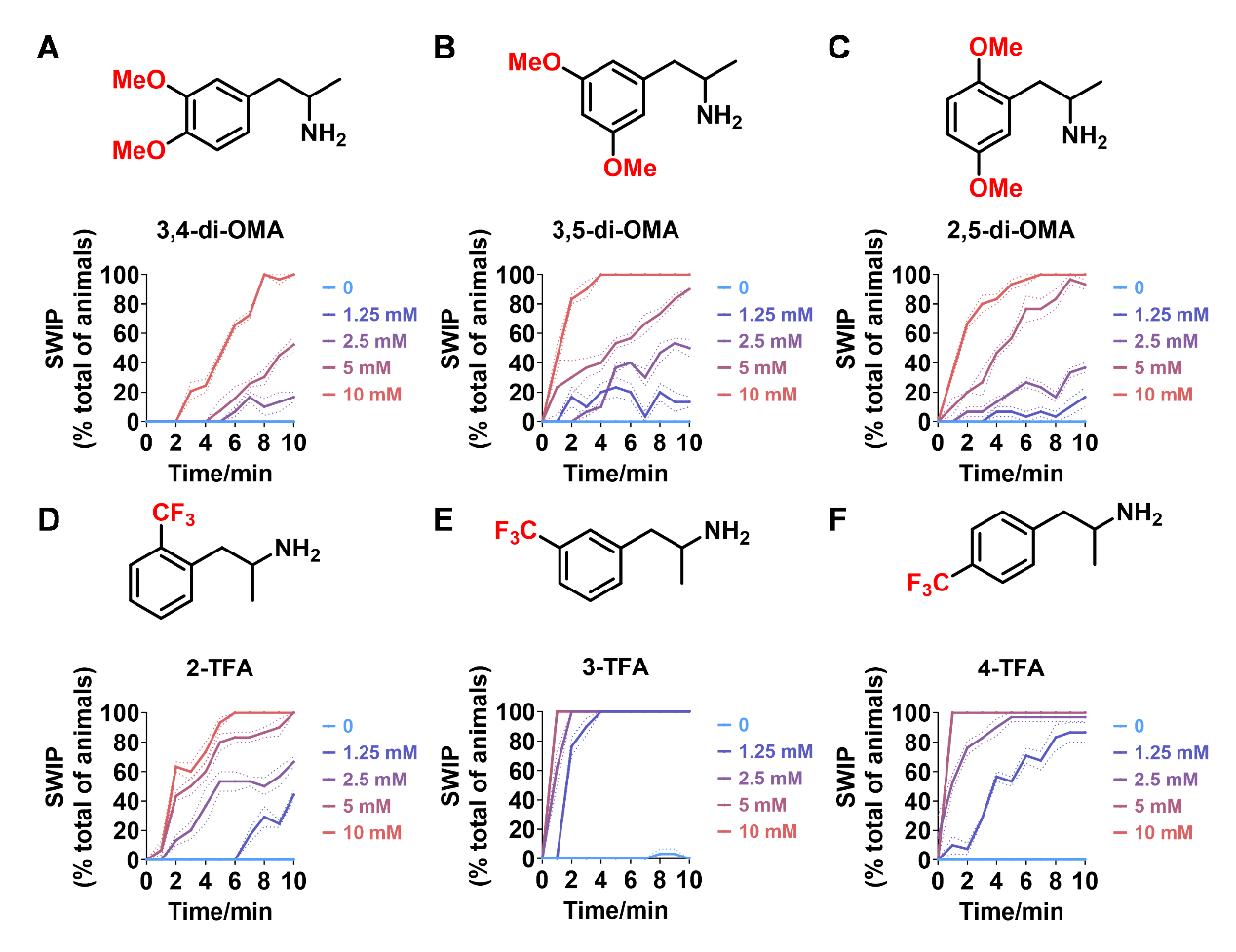


**Figure S6.** Structural and functional comparison of ATS-induced SWIP behavior in *C. elegans*. (**A-C)** Chemical structures of OCH_3_ disubstituted at the *meta*- and *para*- position (3,4-di-OMA, A), *meta*- position (3,5-di-OMA, B) and *ortho*- and *para*- position (2,5-di-OMA, C) in AMPH, and their related SWIP behavior at concentrations of 0, 1.25, 2.5, 5, and 10 mM in N2. (D-F) Chemical structures of CF_3_ substituent at the *ortho*-position (2-TFA, D), *meta*-position (3-TFA, E) and *para*- position (3-TFA, F) in AMPH and its related SWIP behavior at concentrations of 0, 1.25, 2.5, 5, and 10 mM in N2. Three independent experiments were conducted.

**
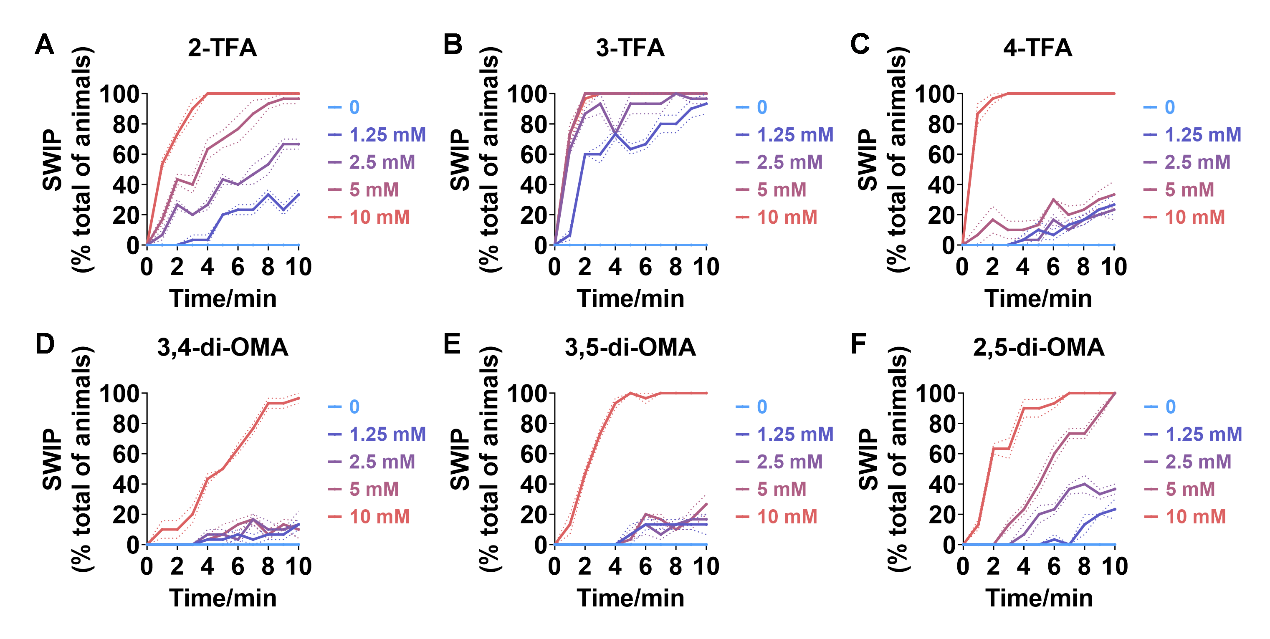
**

**Figure S7.** Structural and functional comparison of ATS-induced SWIP behavior in *dop-3* mutants. **(**A) SWIP behaviors of CF_3_ (2-TFA) substituent at the *ortho*-position in AMPH at concentrations of 0, 1.25, 2.5, 5, and 10 mM in *dop-3* mutants. (B) SWIP behaviors of CF_3_ (3-TFA) substituent at the *meta*-position in AMPH at concentrations of 0, 1.25, 2.5, 5, and 10 mM in *dop-3* mutants. (C) SWIP behaviors of CF_3_ (4-TFA) substituent at the *para-*position in AMPH at concentrations of 0, 1.25, 2.5, 5, and 10 mM in *dop-3* mutants. (D) SWIP behaviors of OCH_3_ disubstituted at the 3’- and 4’- position (3,4-di-OMA) in AMPH at concentrations of 0, 1.25, 2.5, 5, and 10 mM in dop-3 mutants. (E) SWIP behaviors of OCH_3_ disubstituted at the 3’- and 5’- position (3,5-di-OMA) in AMPH at concentrations of 0, 1.25, 2.5, 5, and 10 mM in dop-3 mutants. (F) SWIP behaviors of OCH_3_ disubstituted at the 2’- and 5’- position (2,5-di-OMA) in AMPH at concentrations of 0, 1.25, 2.5, 5, and 10 mM in *dop-3* mutants. Three independent experiments were conducted.

**
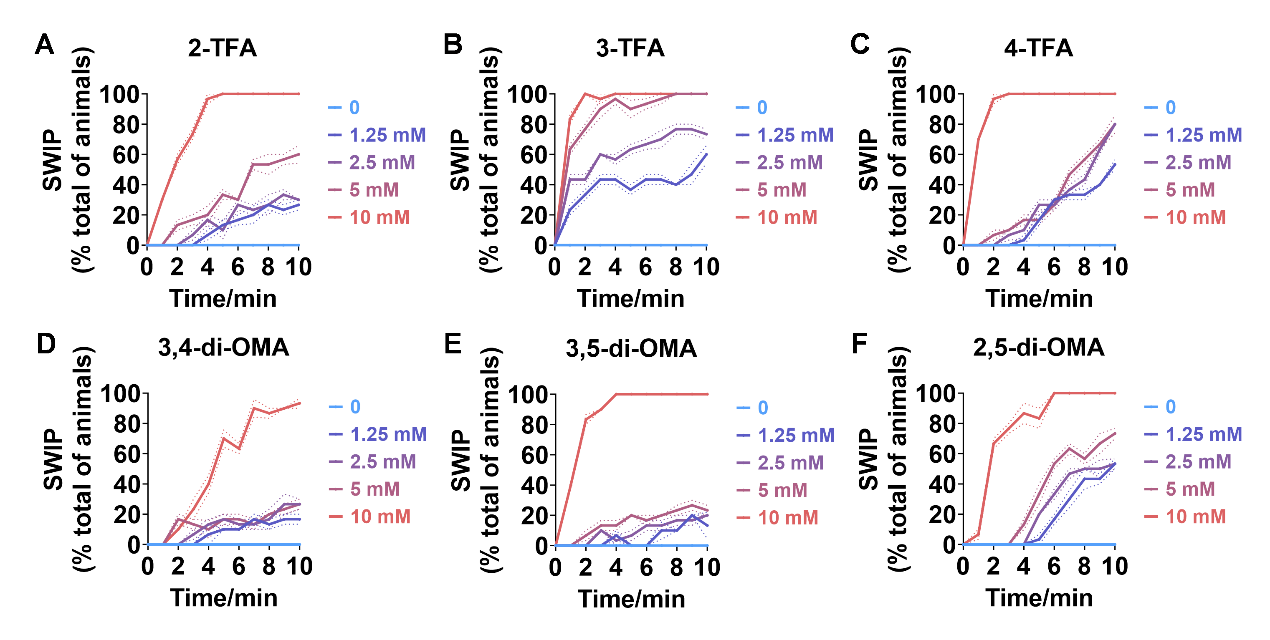
**

**Figure S8.** Structural and functional comparison of ATS-induced SWIP behavior in *ser-4* mutants. (A) SWIP behaviors of CF_3_ (2-TFA) substituent at the *ortho*-position in AMPH at concentrations of 0, 1.25, 2.5, 5, and 10 mM in *ser-4* mutants. (B) SWIP behaviors of CF_3_ (3-TFA) substituent at the *meta*-position in AMPH at concentrations of 0, 1.25, 2.5, 5, and 10 mM in *ser-4* mutants. (C) SWIP behaviors of CF_3_ (4-TFA) substituent at the *para-*position in AMPH at concentrations of 0, 1.25, 2.5, 5, and 10 mM in *ser-4* mutants. (D) SWIP behaviors of OCH_3_ disubstituted at the 3’- and 4’- position (3,4-di-OMA) in AMPH at concentrations of 0, 1.25, 2.5, 5, and 10 mM in *ser-4* mutants. (E) SWIP behaviors of OCH_3_ disubstituted at the 3’- and 5’- position (3,5-di-OMA) in AMPH at concentrations of 0, 1.25, 2.5, 5, and 10 mM in ser-4 mutants. (F) SWIP behaviors of OCH_3_ disubstituted at the 2’- and 5’- position (2,5-di-OMA) in AMPH at concentrations of 0, 1.25, 2.5, 5, and 10 mM in *ser-4* mutants. Three independent experiments were conducted.

**
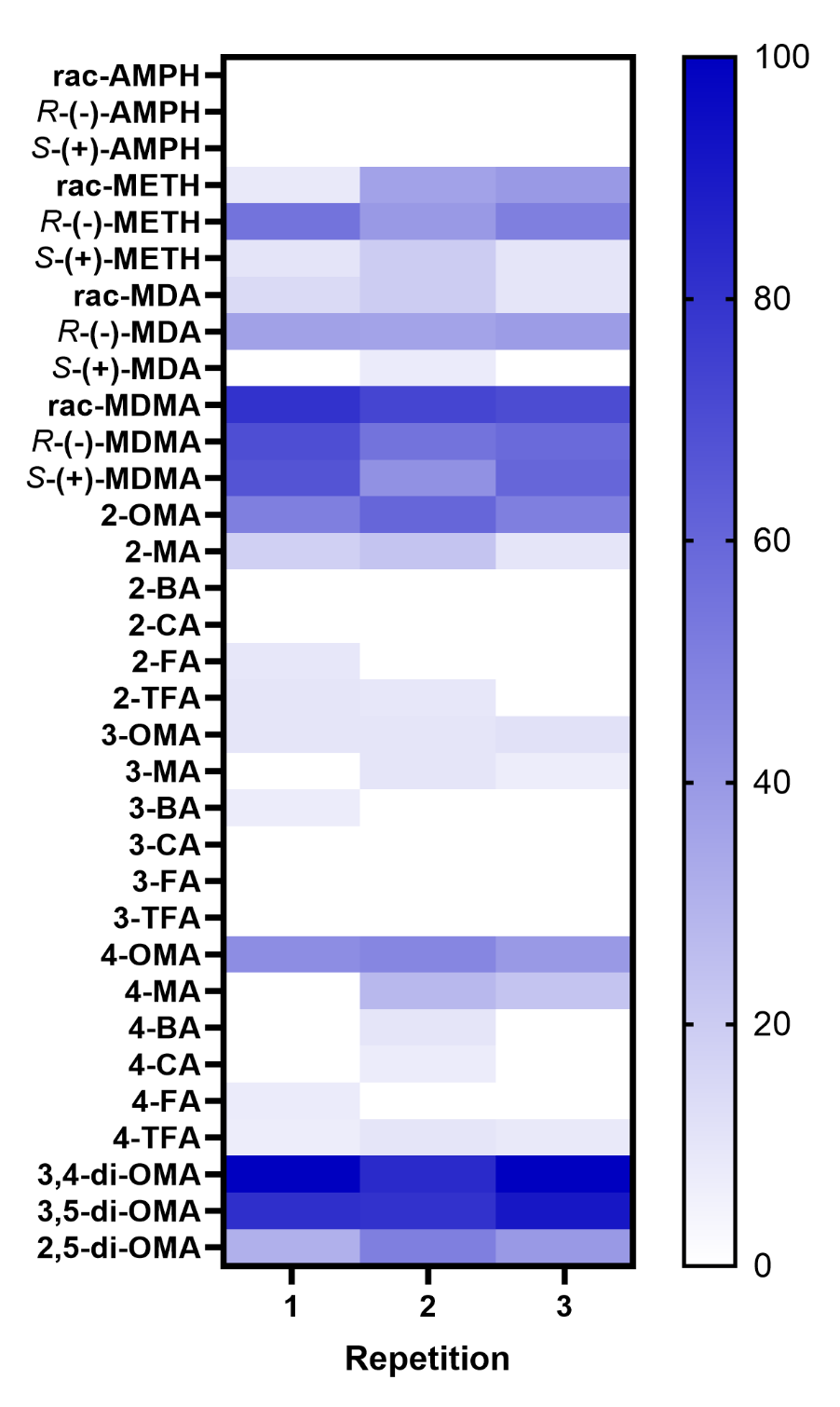
**

**Figure S9.** Recovery from ATS-induced SWIP in *C. elegans*. The heatmap illustrates the recovery rates of wild-type *C. elegans* (N2 strain) following exposure to various ATS. Adult *C. elegans* were exposed to ATS solutions for 10 minutes on Day 1, after which they were transferred to ATS-free solution. Recovery from paralysis was assessed by scoring the return to normal swimming behavior 10 minutes post-transfer. The heatmap shows recovery rates across experimental replicates (x-axis) for each ATS tested (y-axis).

**NMR spectra (^1^H, ^13^C) of representative compounds**


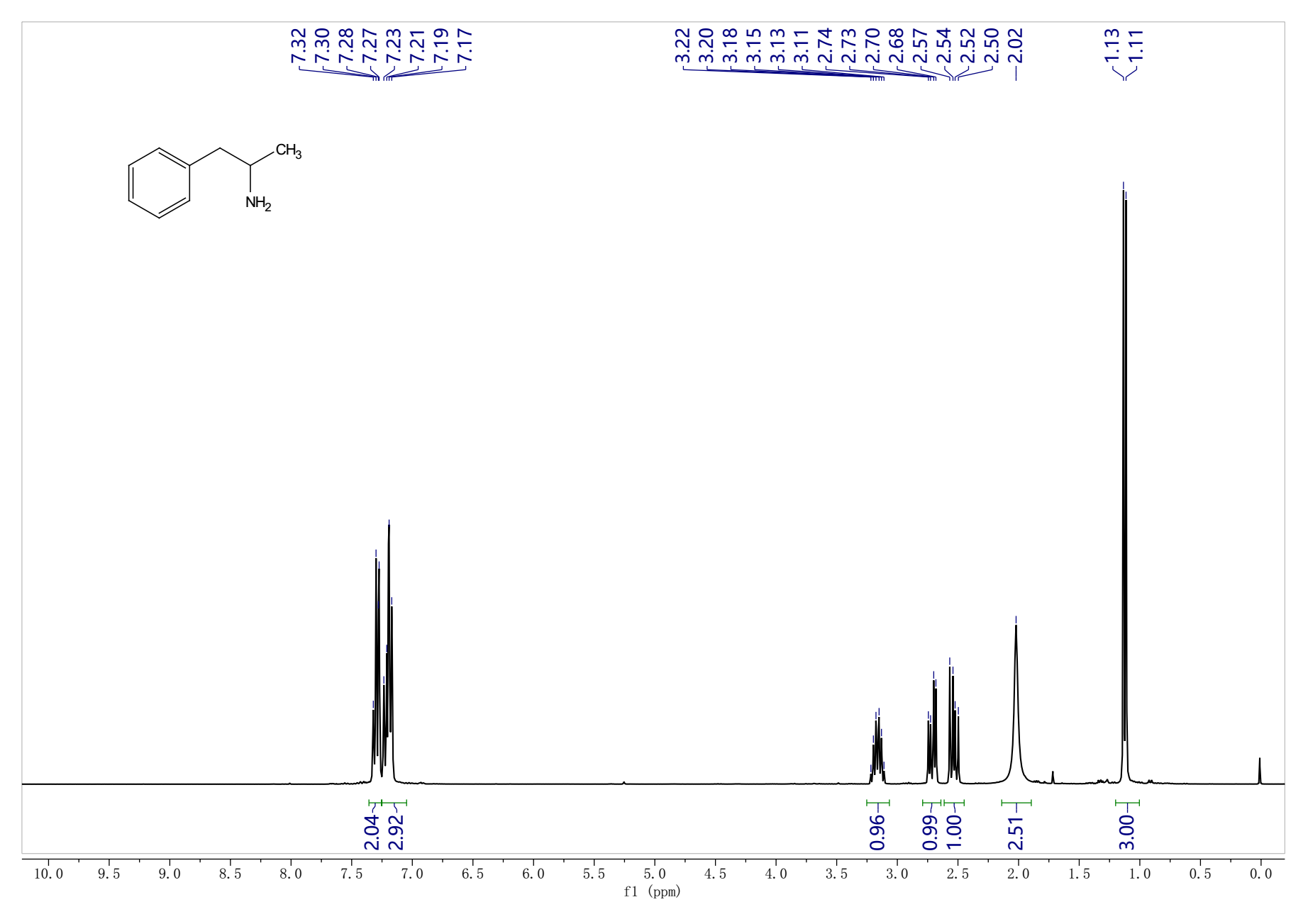


**Figure S10.** ^1^H NMR spectrum of ***rac*-AMPH**


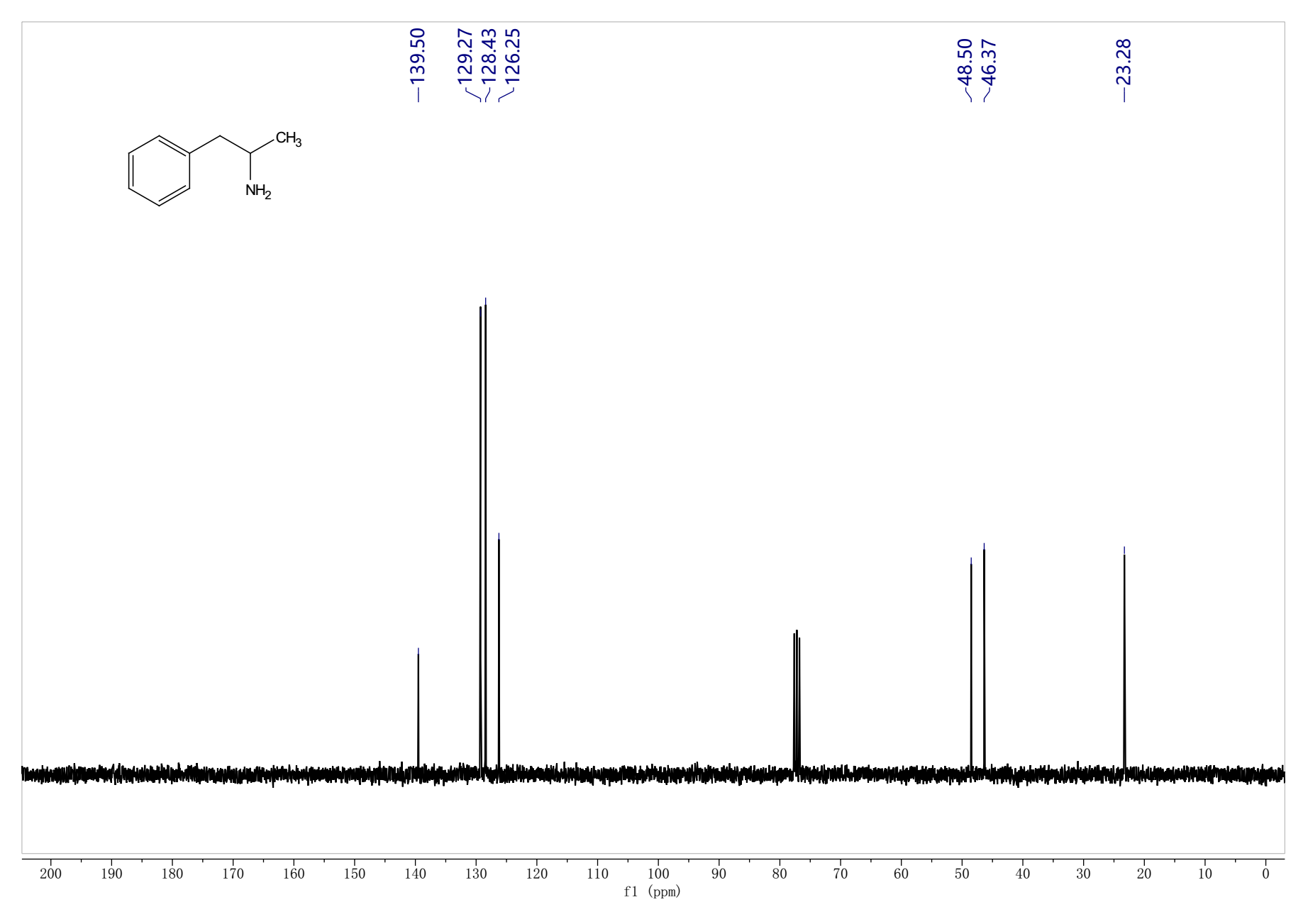


**Figure S11.** ^13^C NMR spectrum of ***rac*-AMPH**


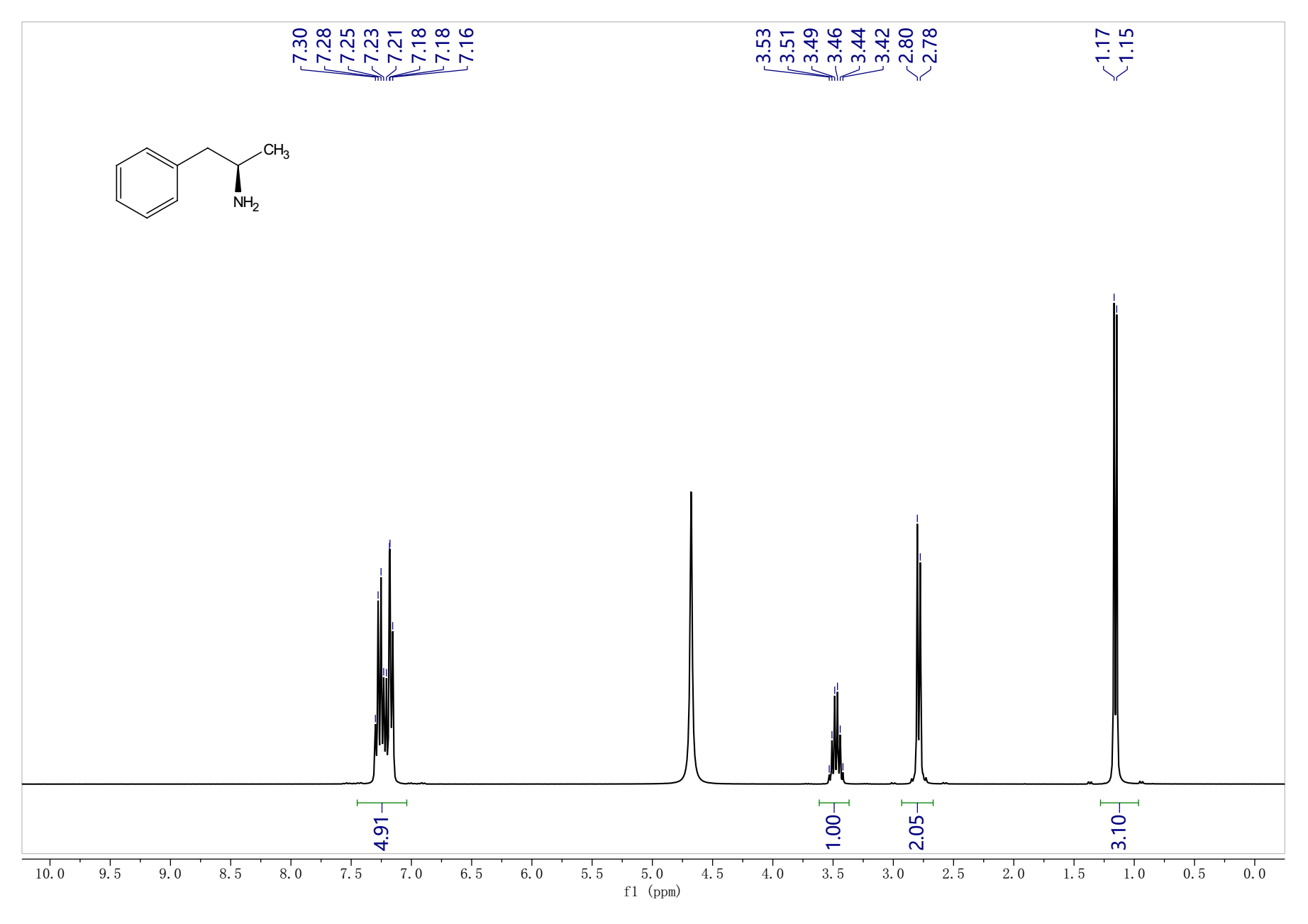


**Figure S12.** ^1^H NMR spectrum of ***R*-(-)-AMPH**


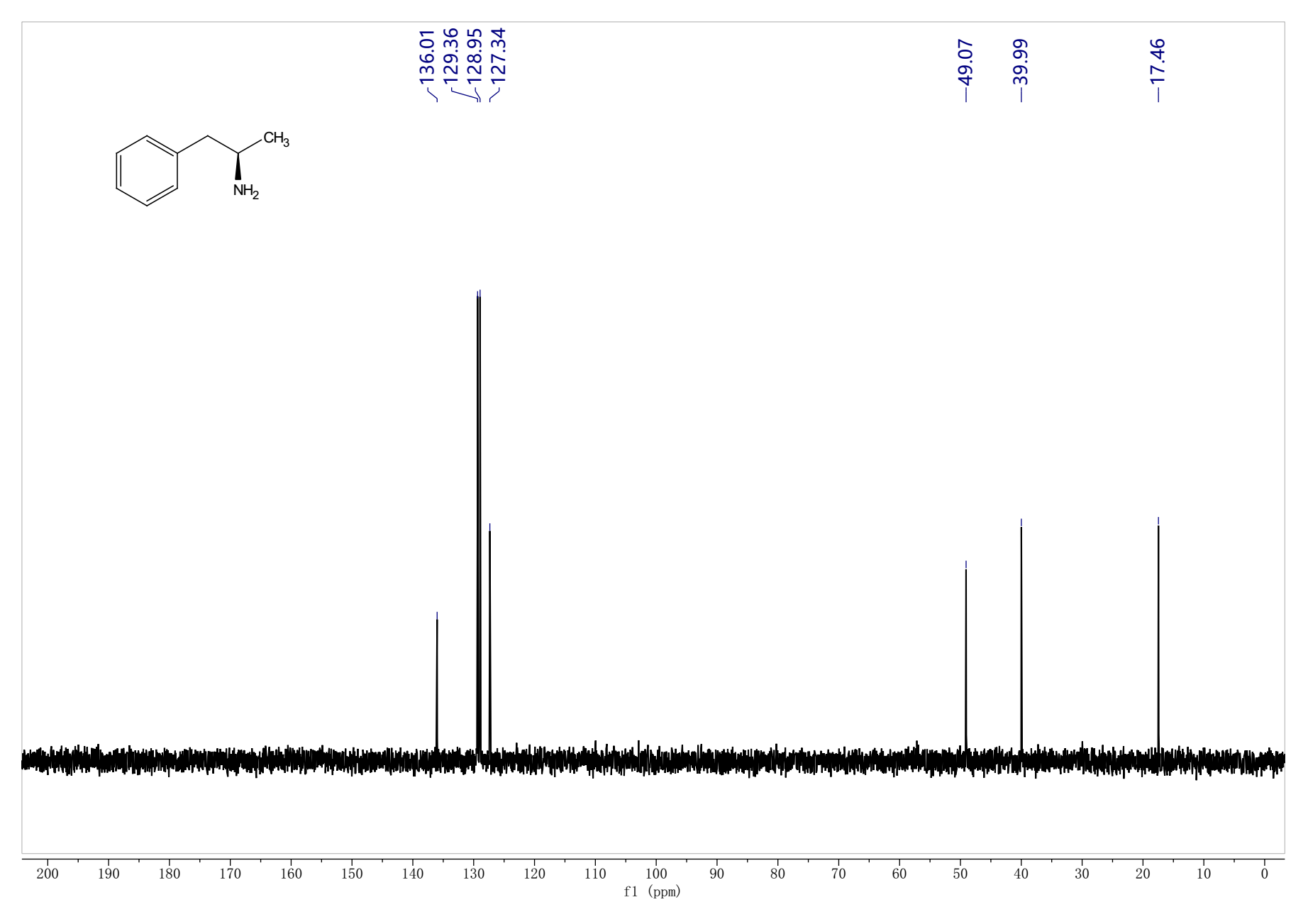


**Figure S13.** ^13^C NMR spectrum of ***R*-(-)-AMPH**


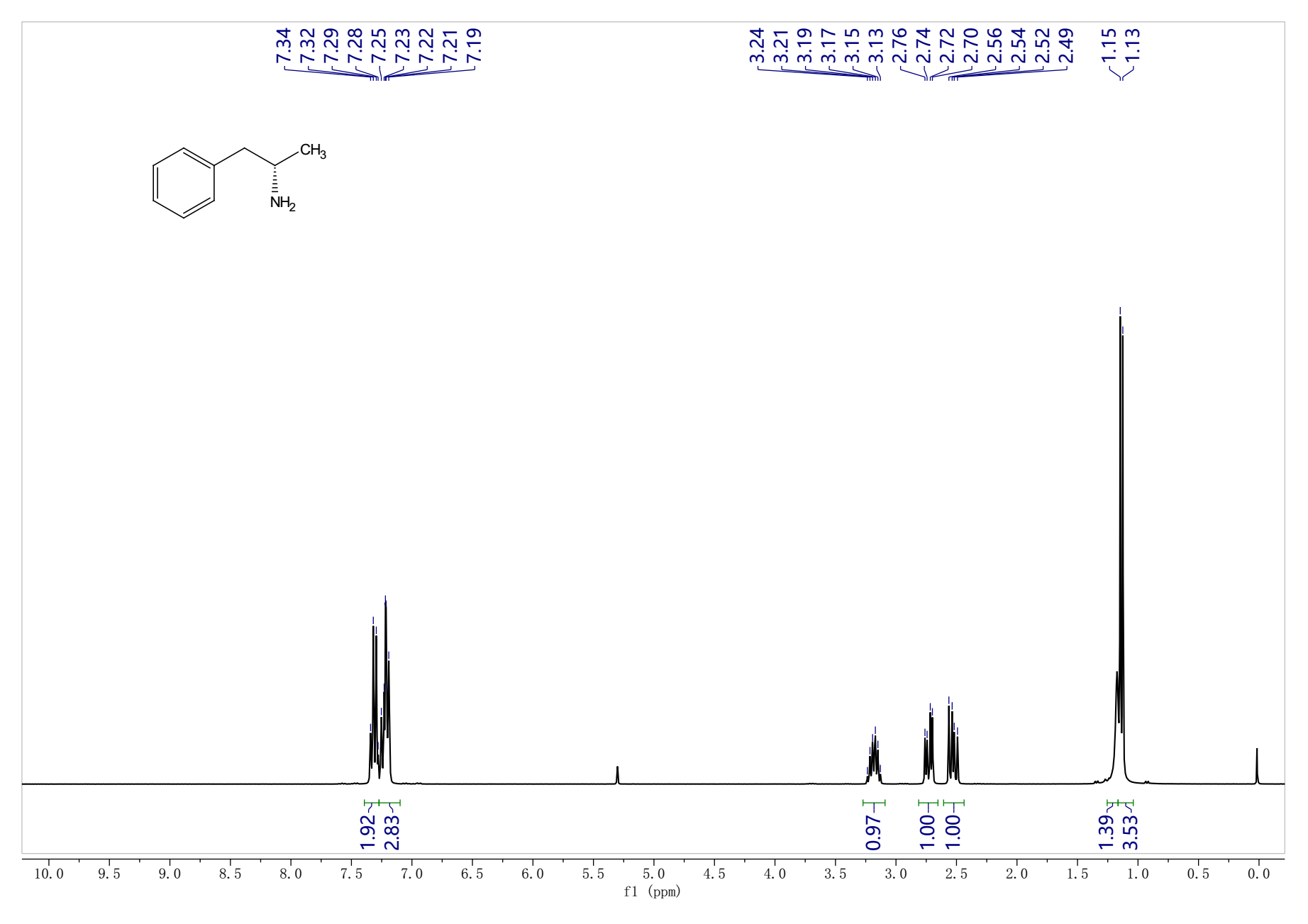


**Figure S14.** ^1^H NMR spectrum of ***S-*(+)-AMPH**


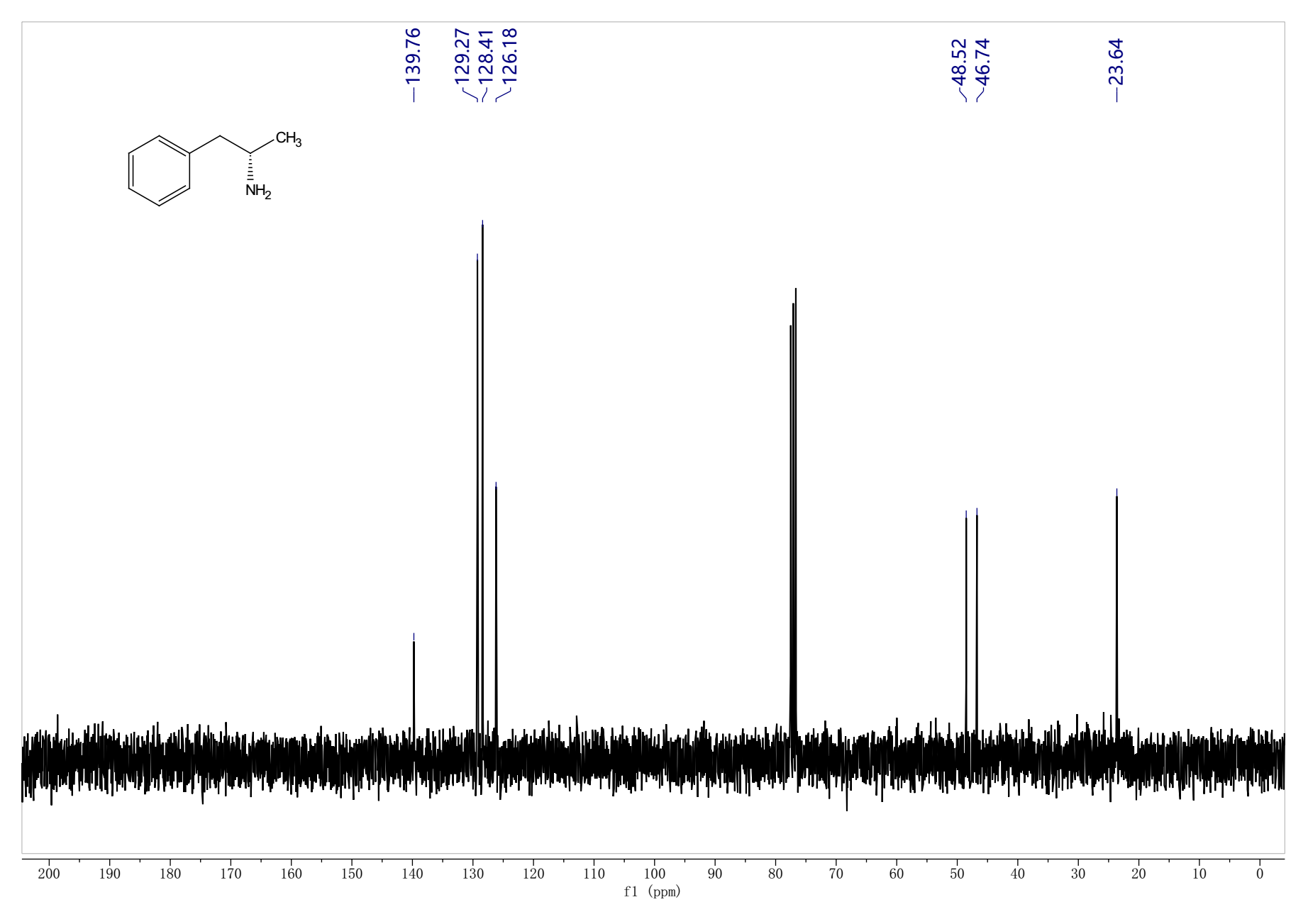


**Figure S15.** ^13^C NMR spectrum of ***S-*(+)-AMPH**


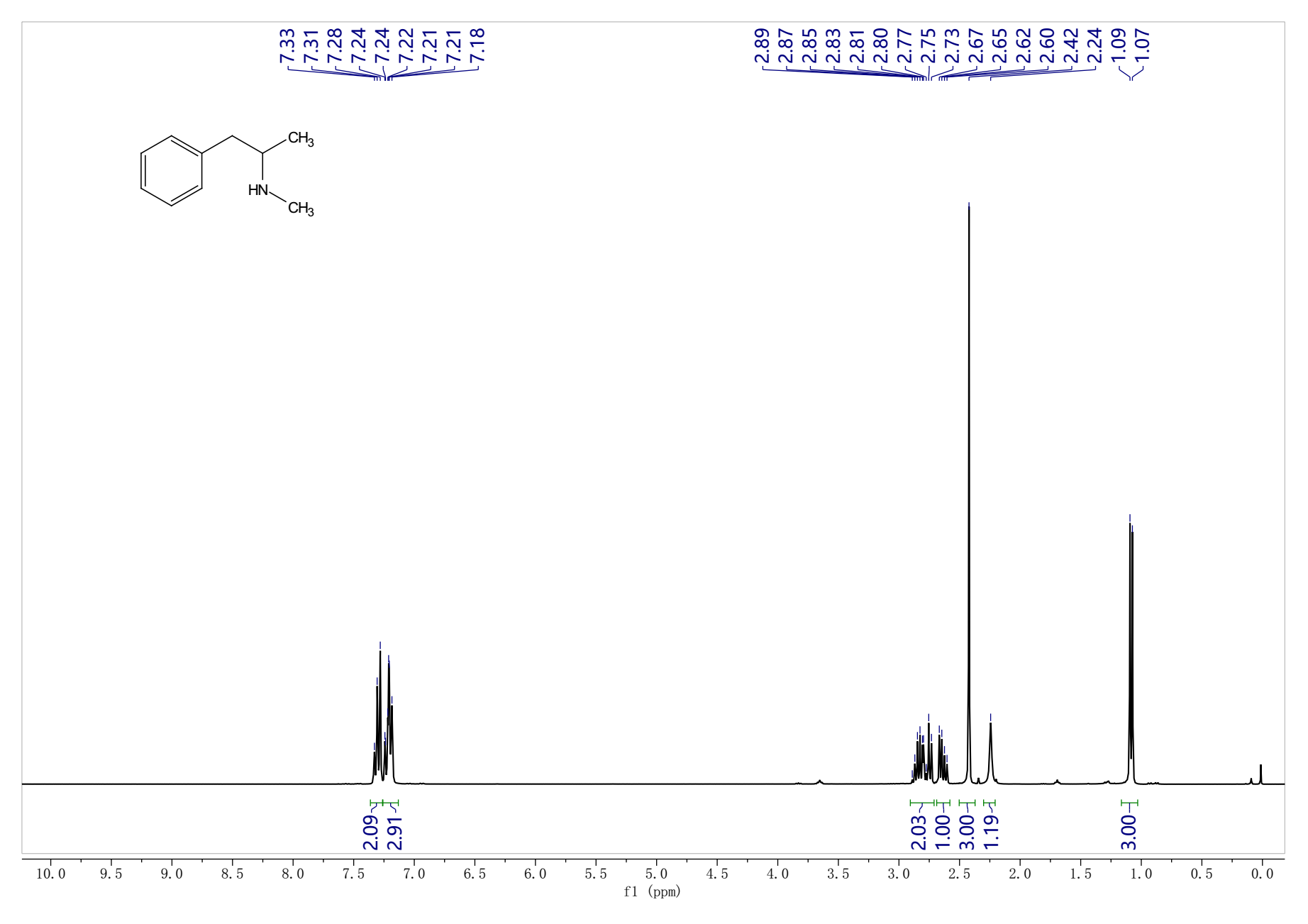


**Figure S16.** ^1^H NMR spectrum of ***rac*-METH**


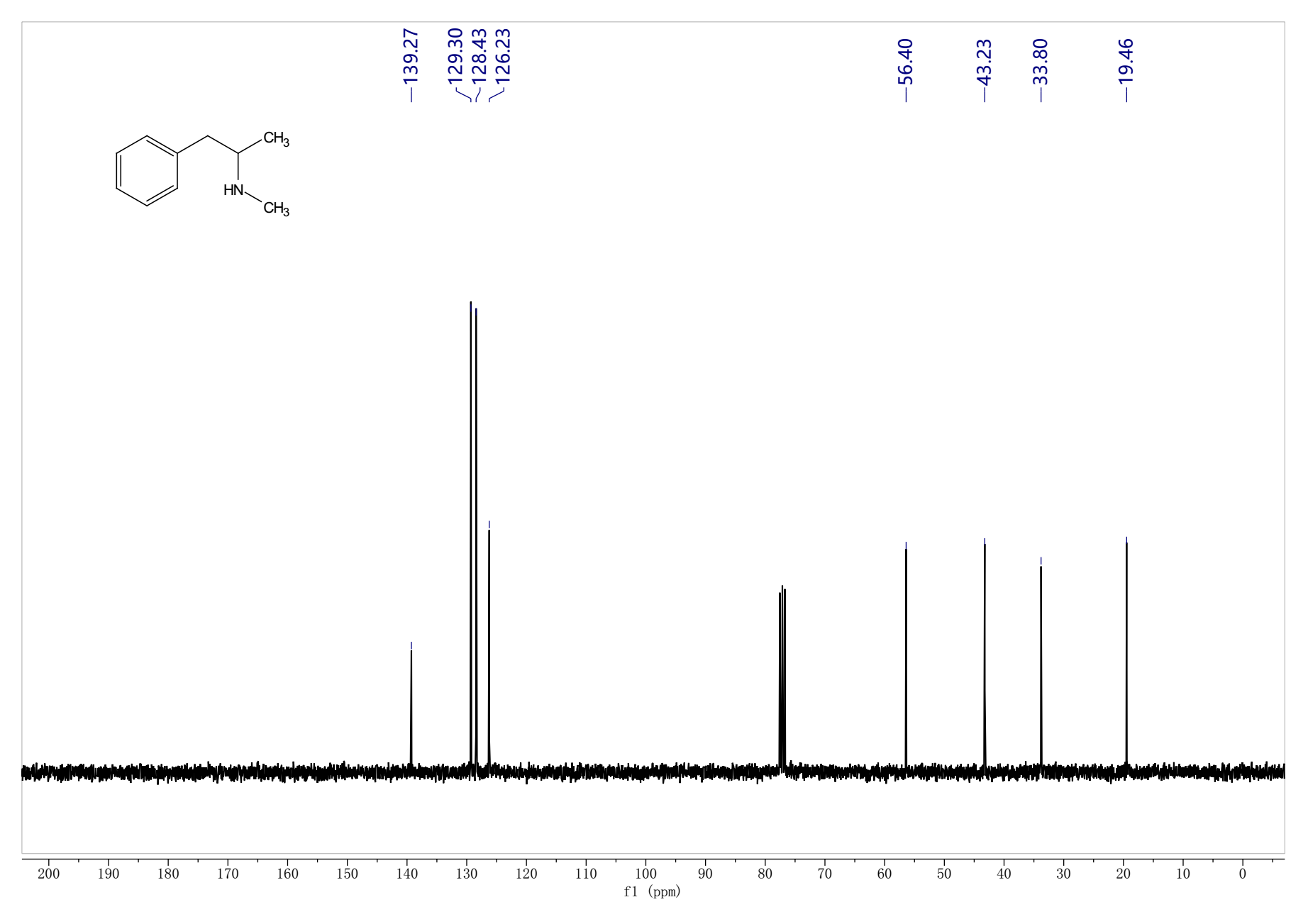


**Figure S17.** ^13^C NMR spectrum of ***rac*-METH**


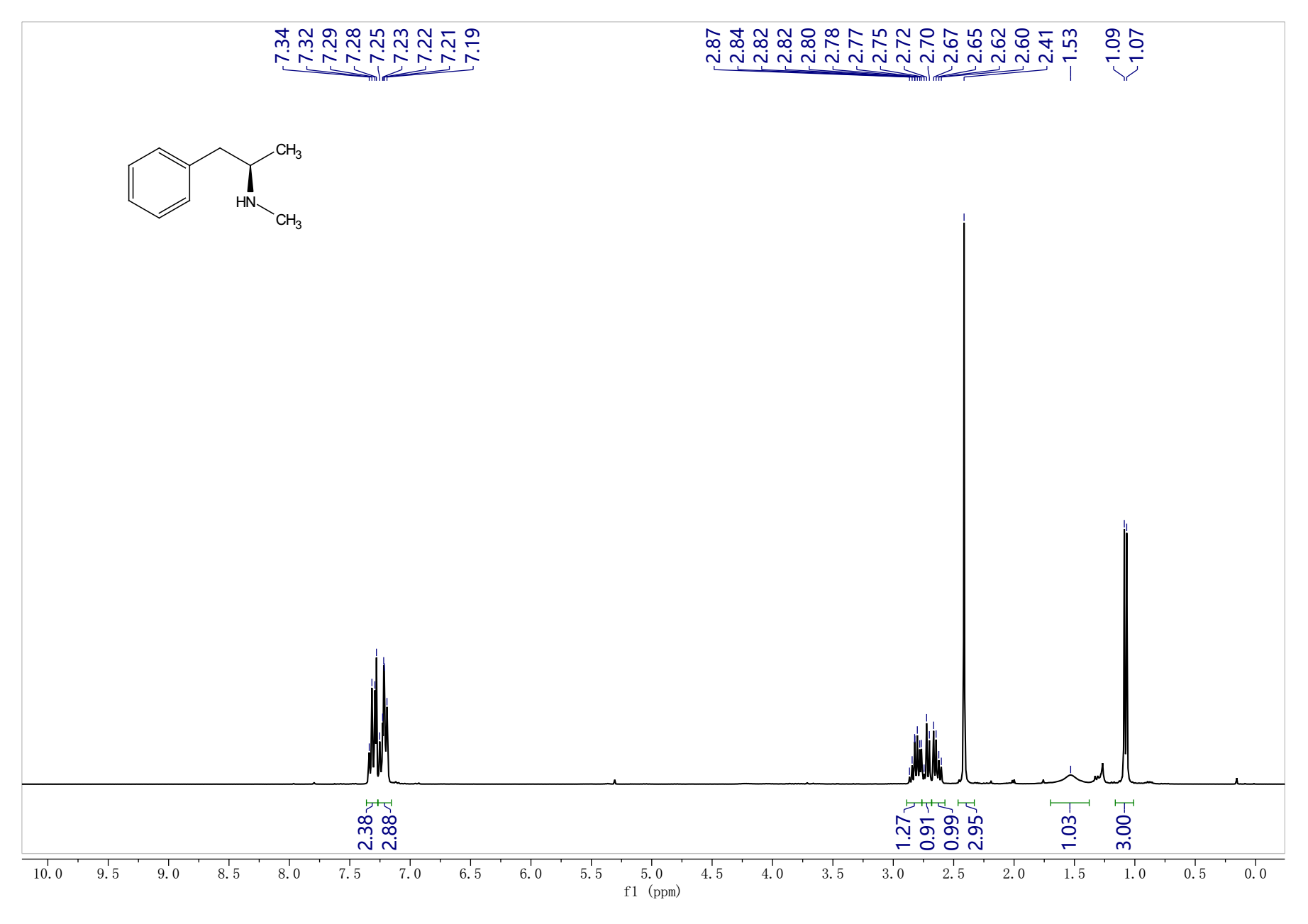


**Figure S18.** ^1^H NMR spectrum of ***R*-(-)-METH**


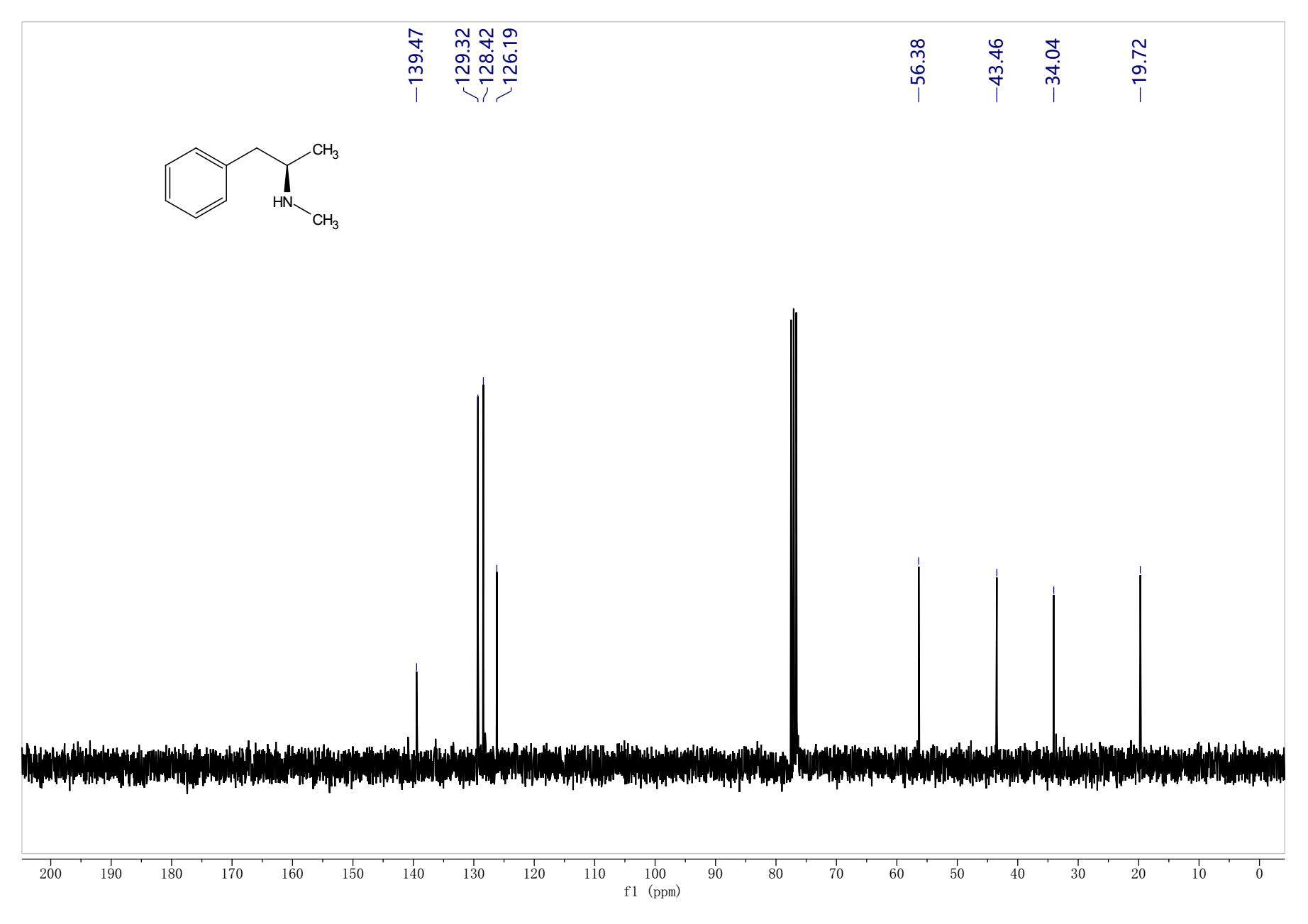


**Figure S19.** ^13^C NMR spectrum of ***R*-(-)-METH**


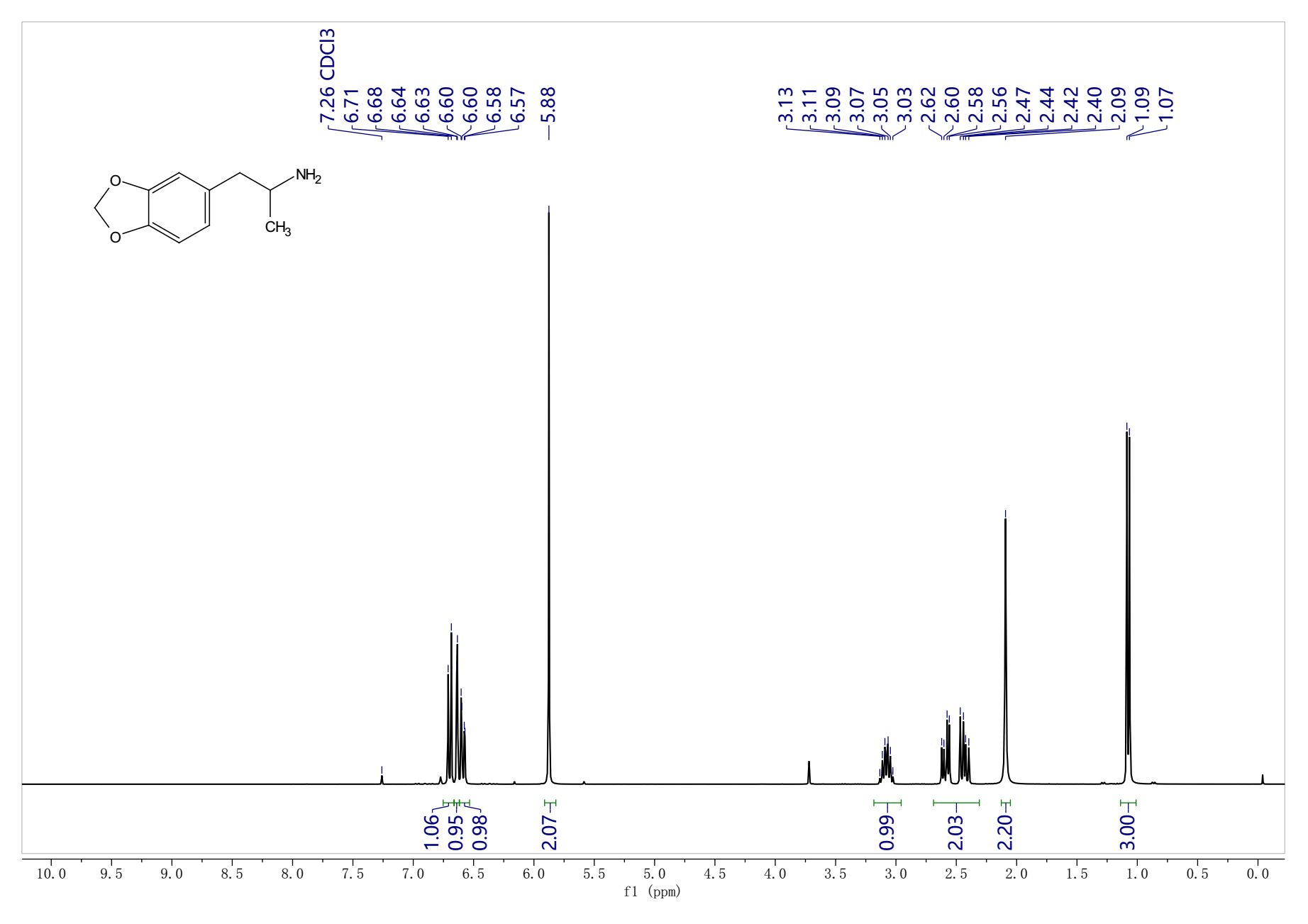


**Figure S20.** ^1^H NMR spectrum of ***rac*-MDA**


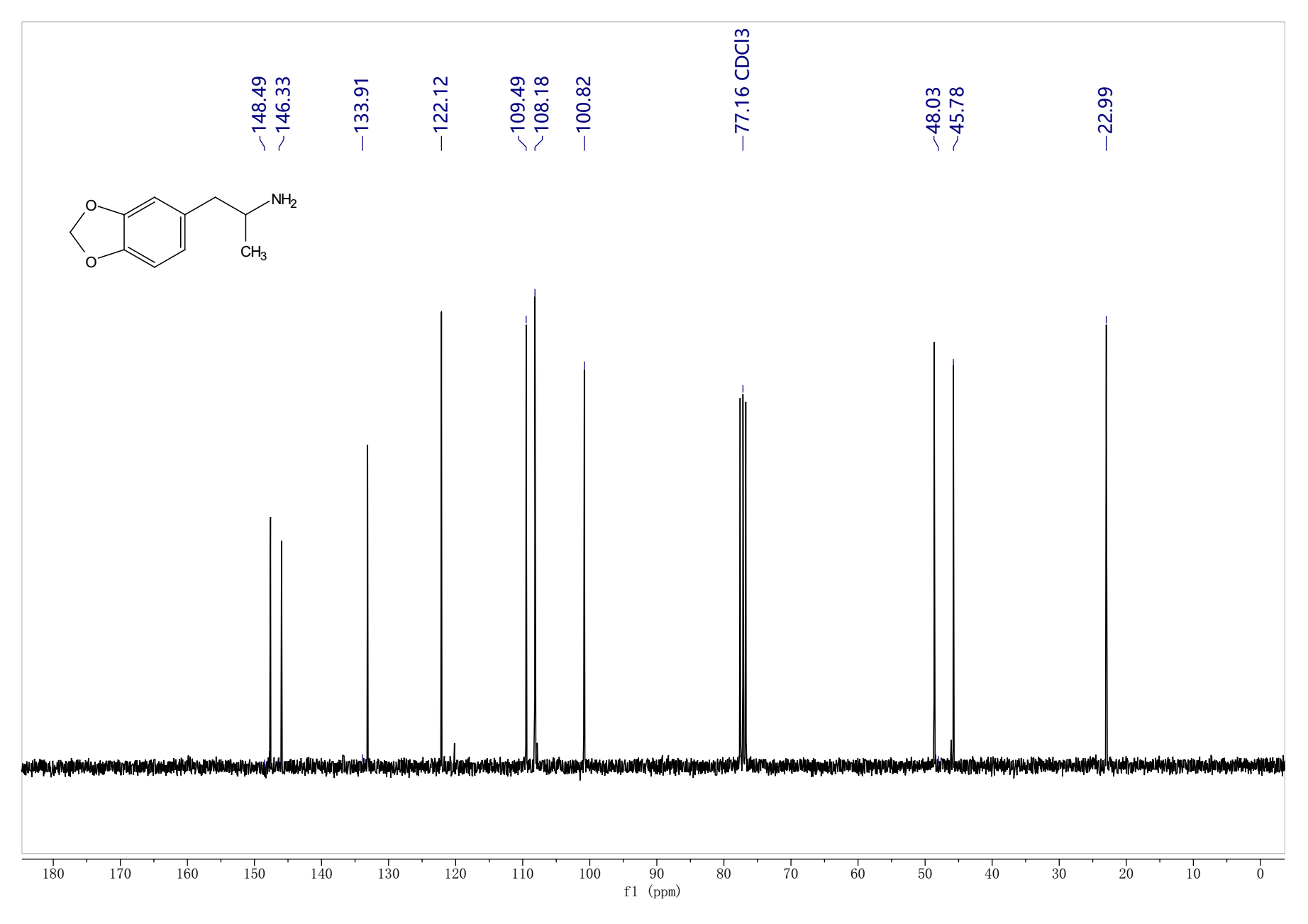


**Figure S21.** ^13^C NMR spectrum of ***rac*-MDA**


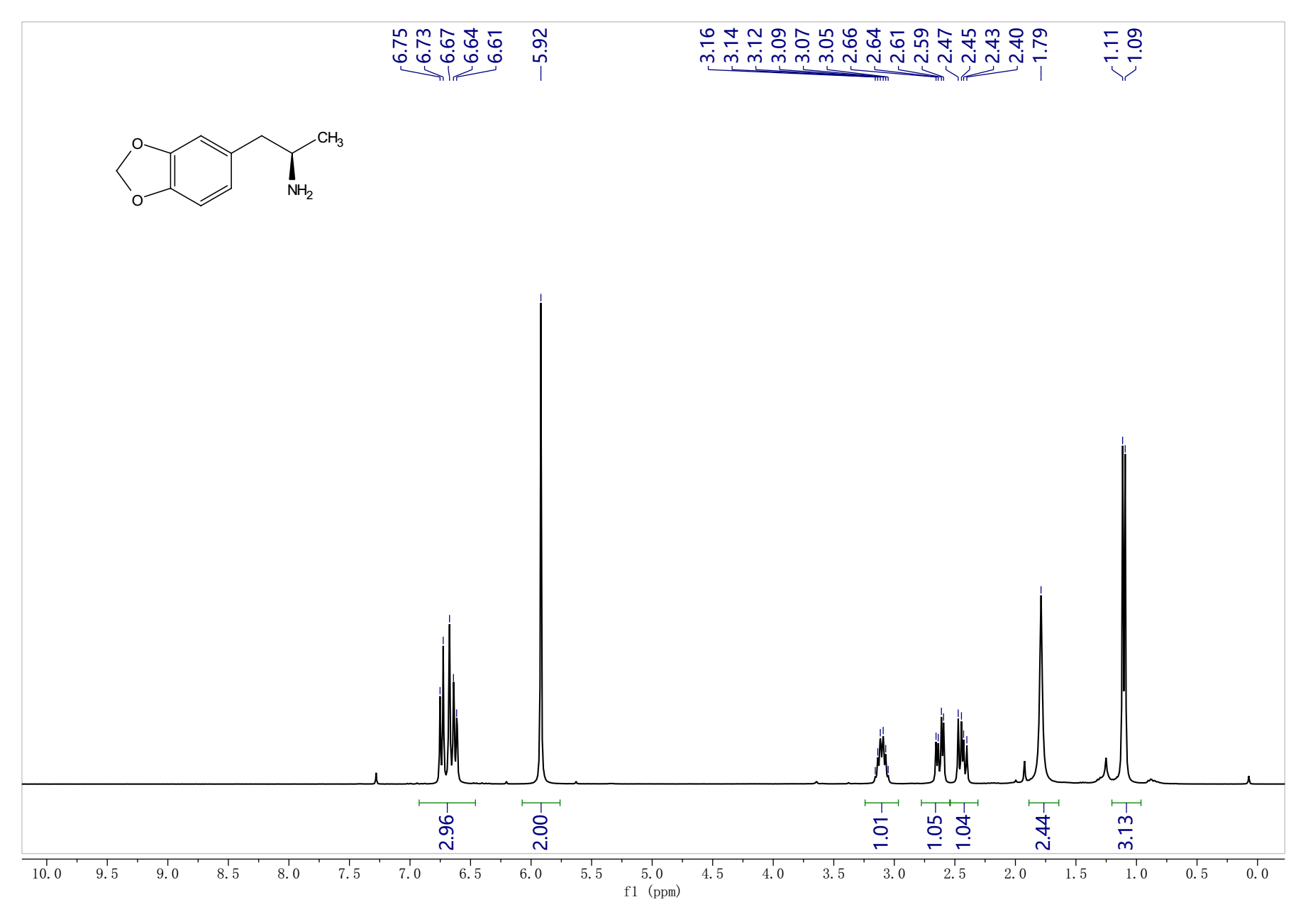


**Figure S22.** ^1^H NMR spectrum of ***R*-(-)-MDA**


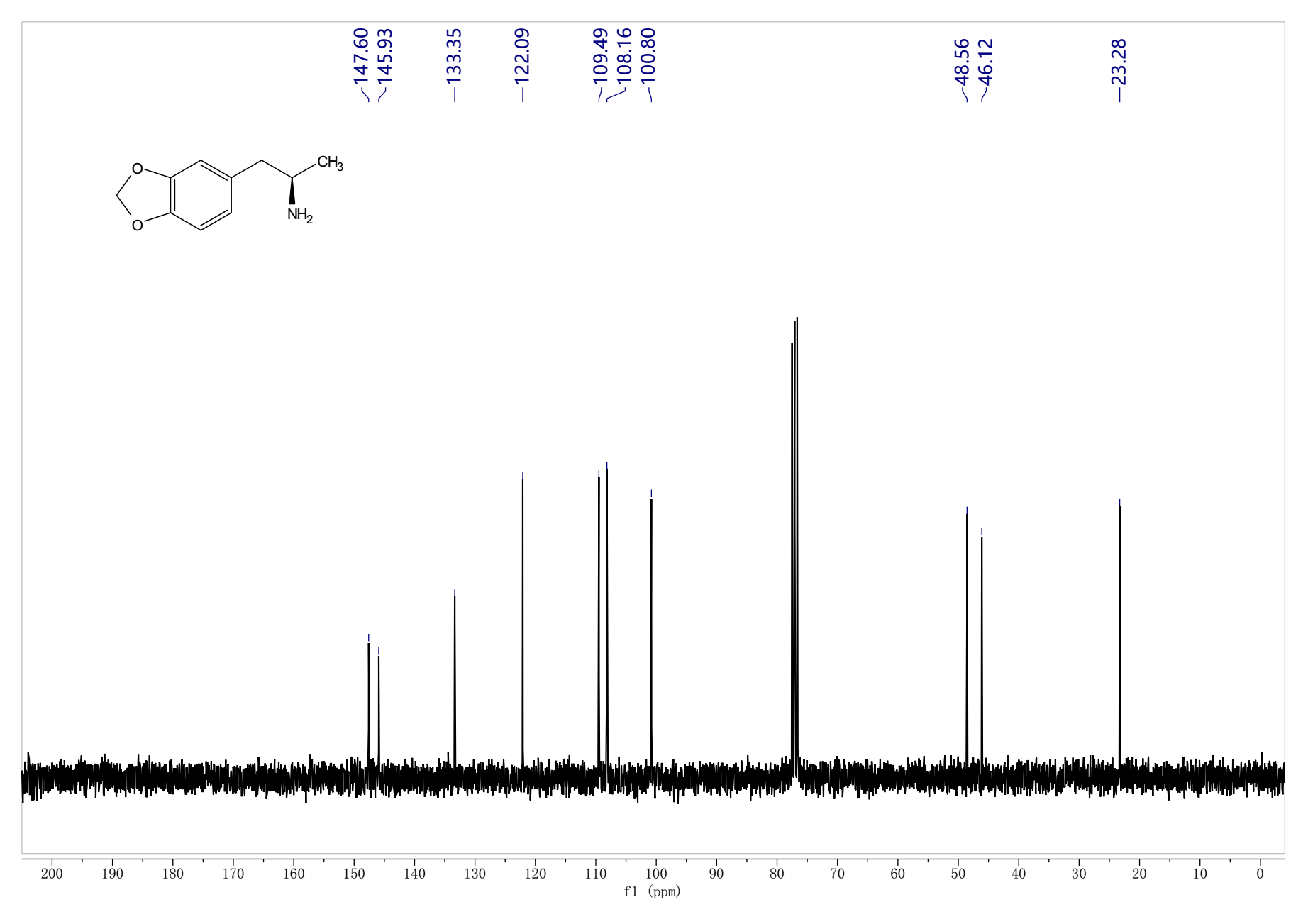


**Figure S23.** ^13^C NMR spectrum of ***R*-(-)-MDA**


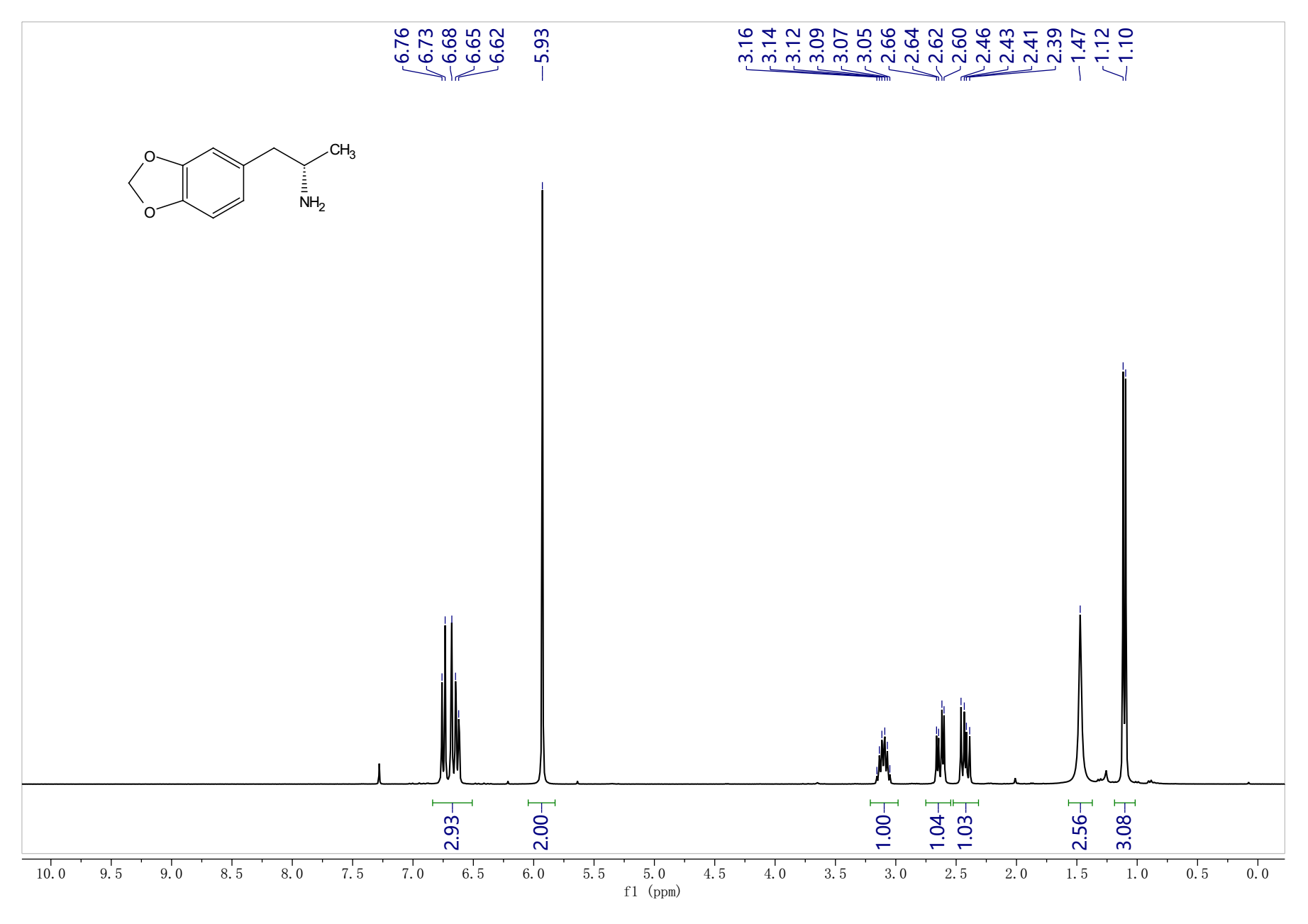


**Figure S24.** ^1^H NMR spectrum of ***S-*(+)-MDA**


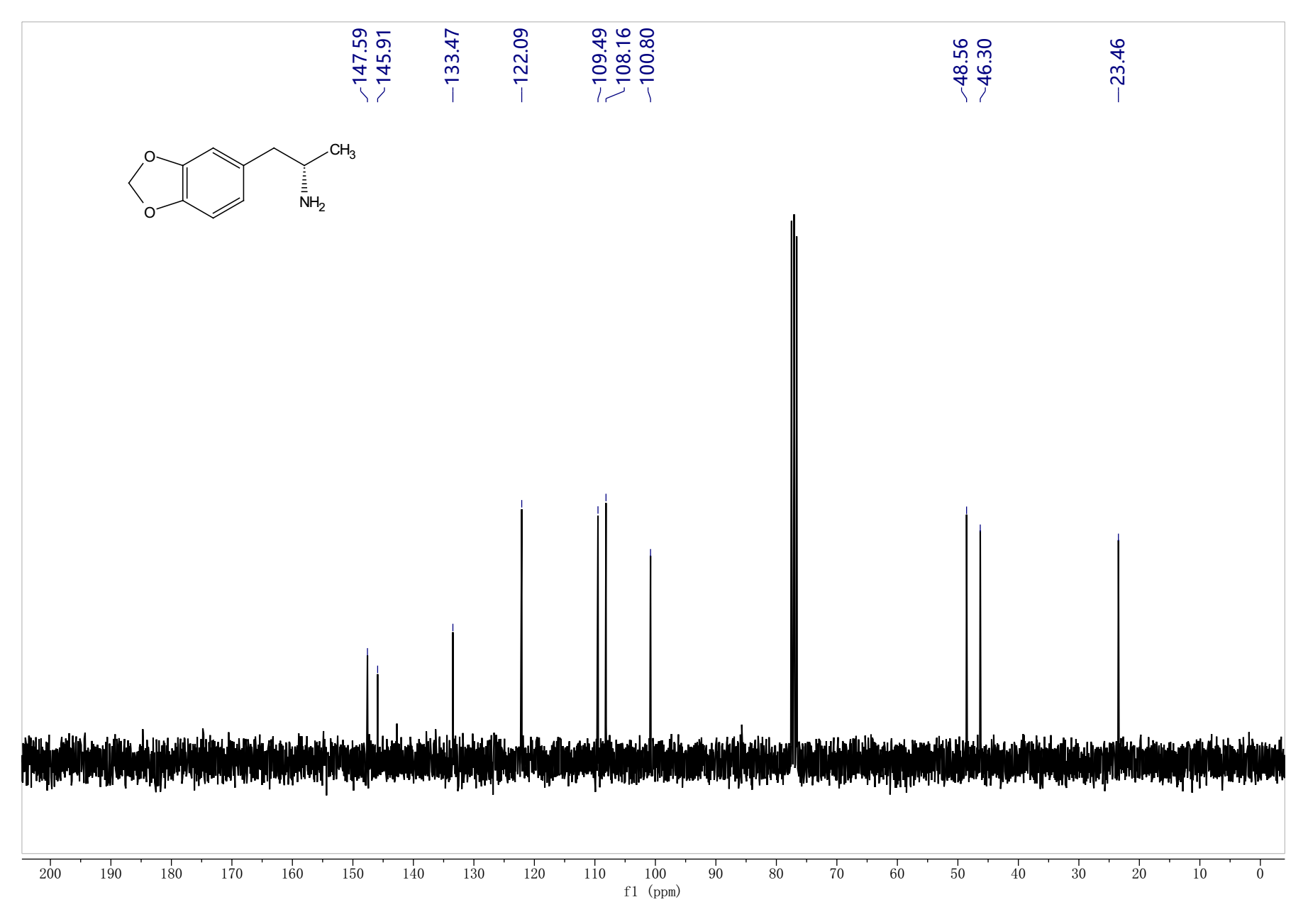


**Figure S25.** ^13^C NMR spectrum of ***S-*(+)-MDA**


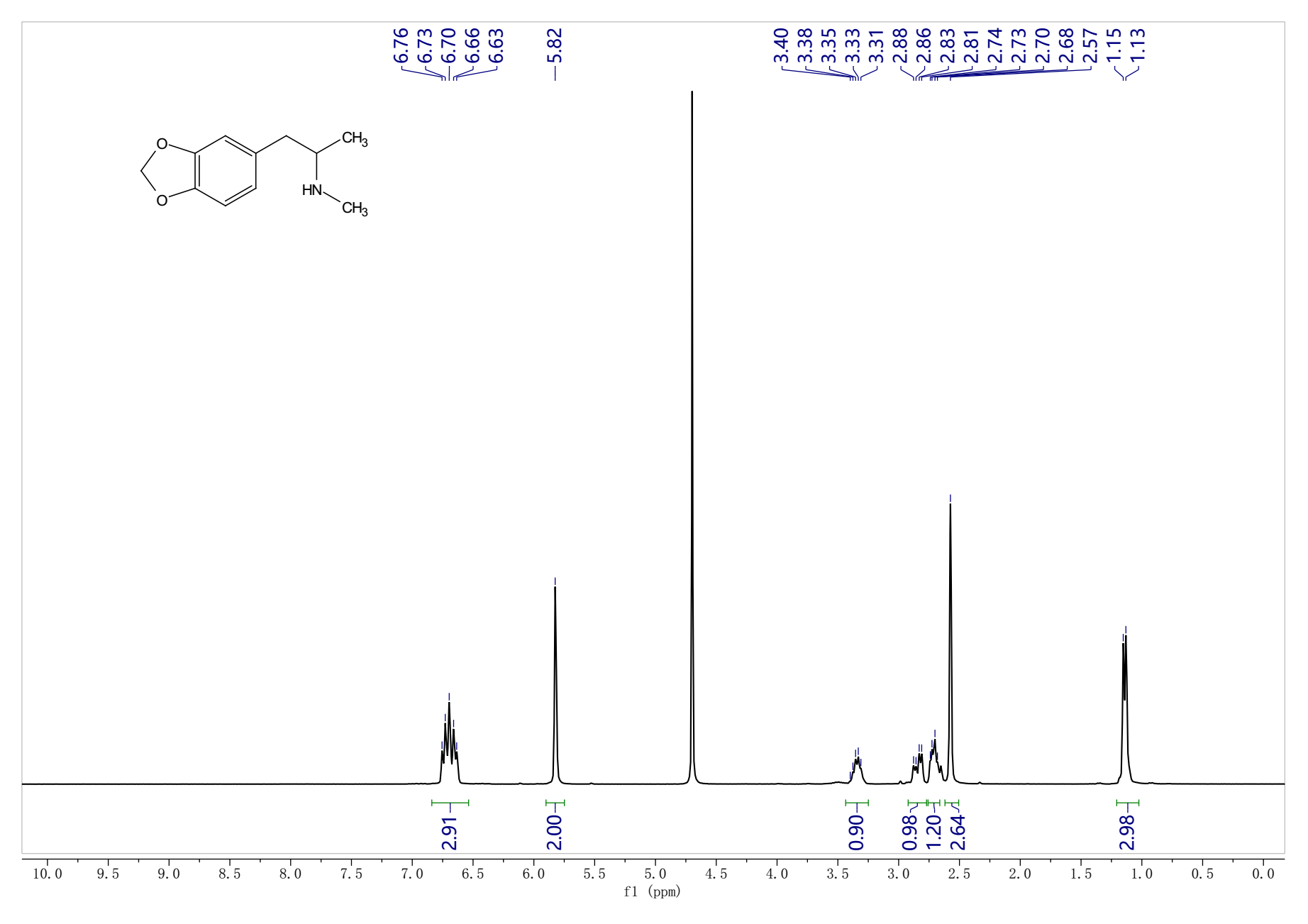


**Figure S26.** ^1^H NMR spectrum of ***rac*-MDMA**


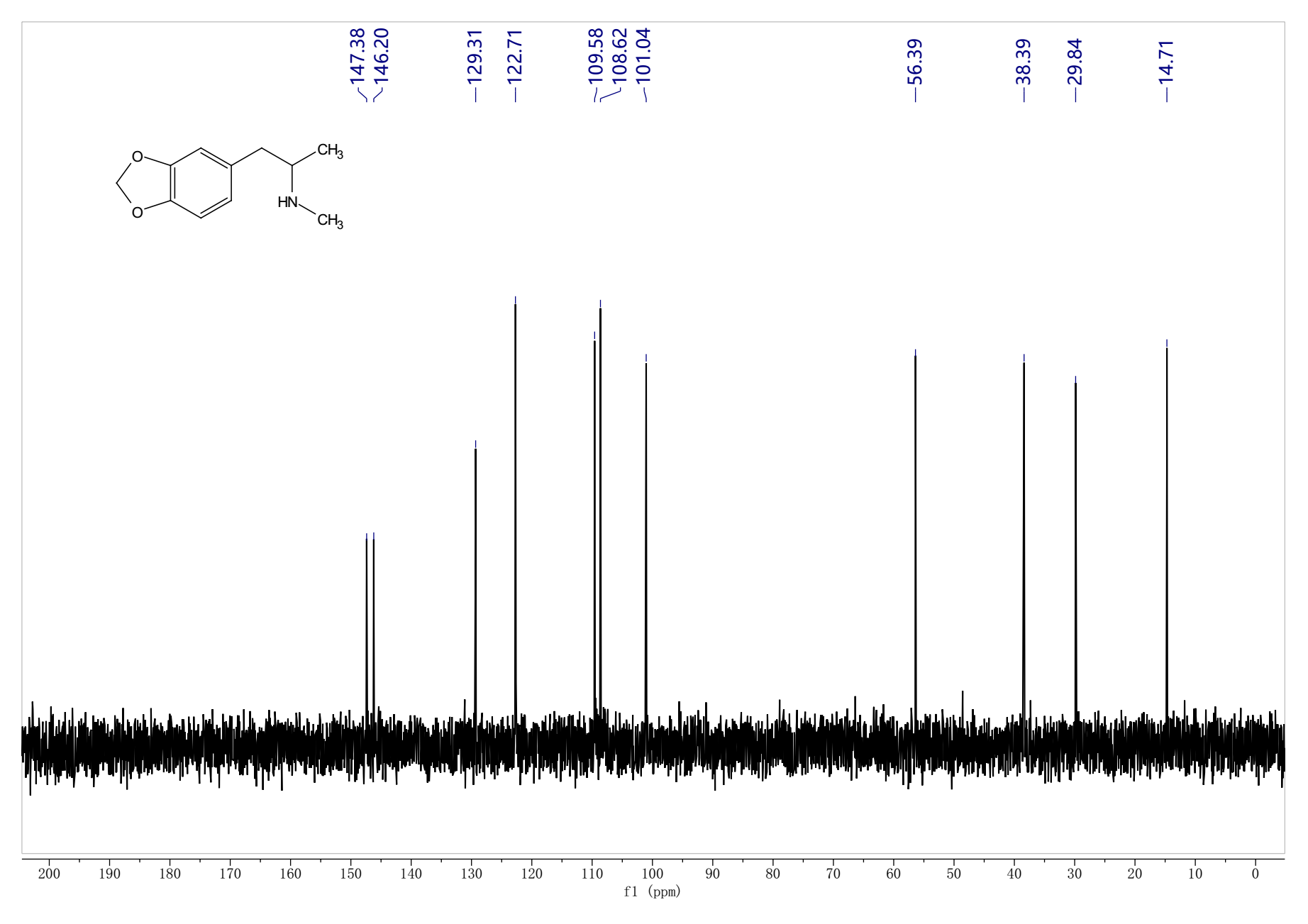


**Figure S27.** ^13^C NMR spectrum of ***rac*-MDMA**


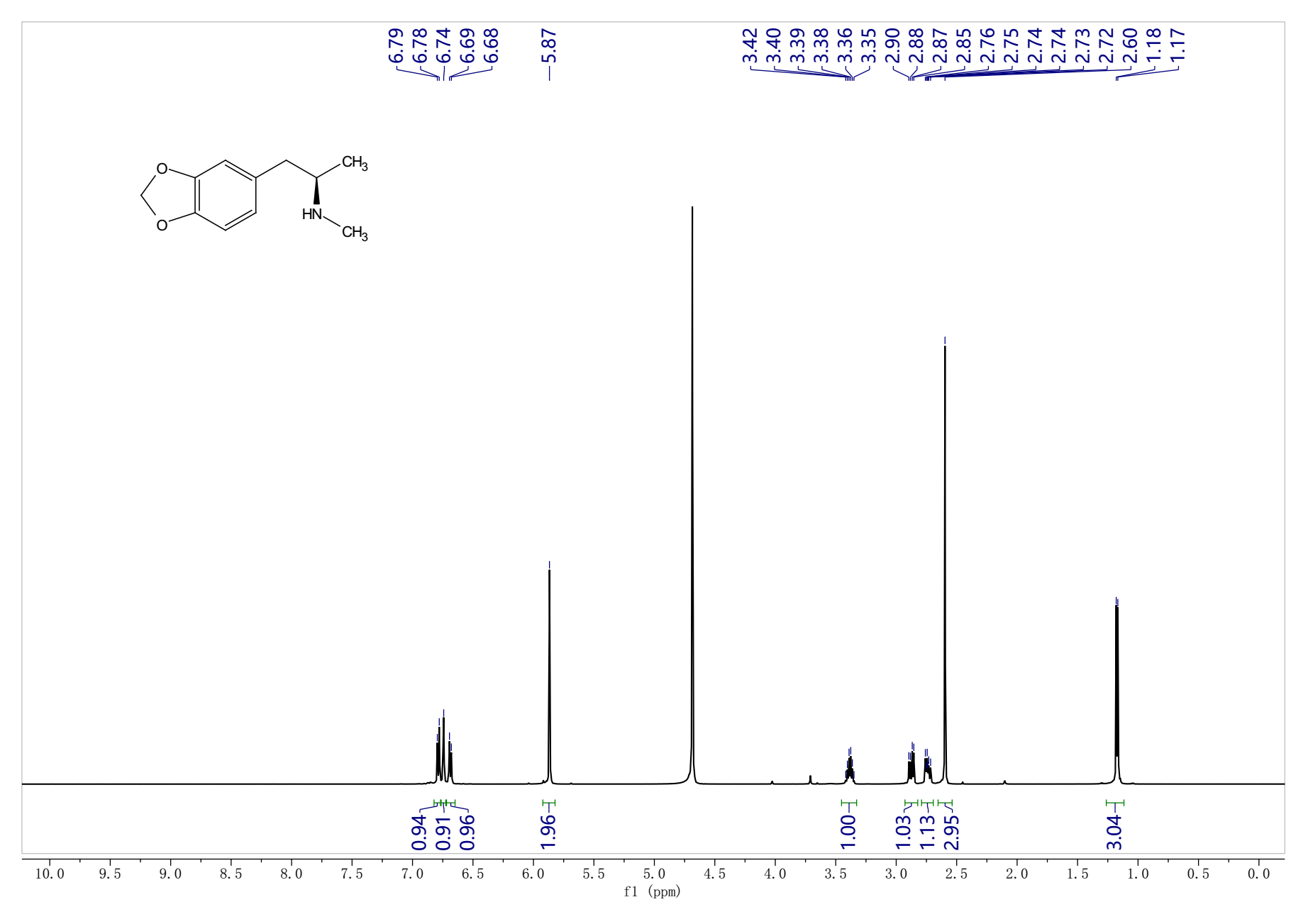


**Figure S28.** ^1^H NMR spectrum of ***R*-(-)-MDMA**


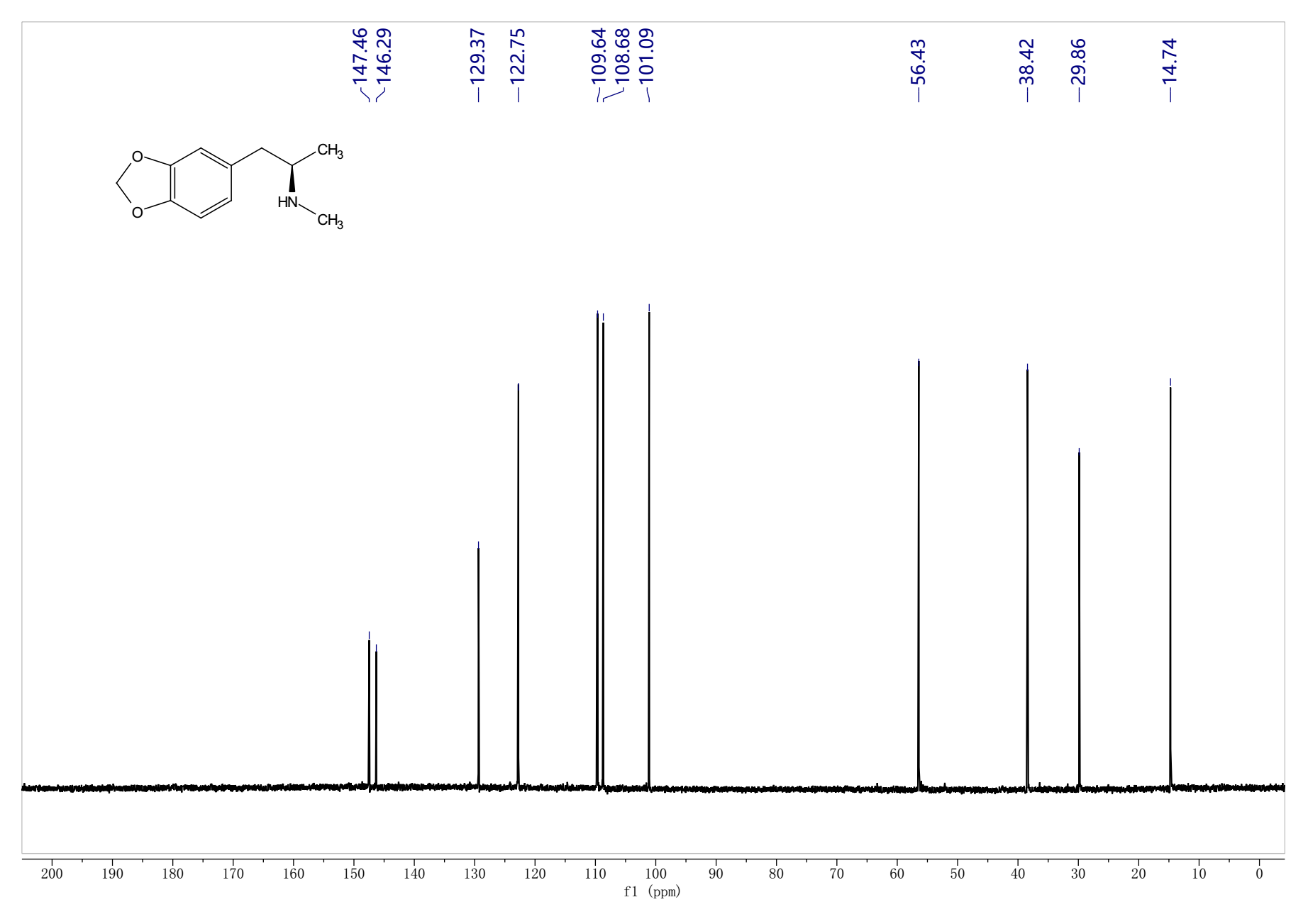


**Figure S29.** ^13^C NMR spectrum of ***R*-(-)-MDMA**


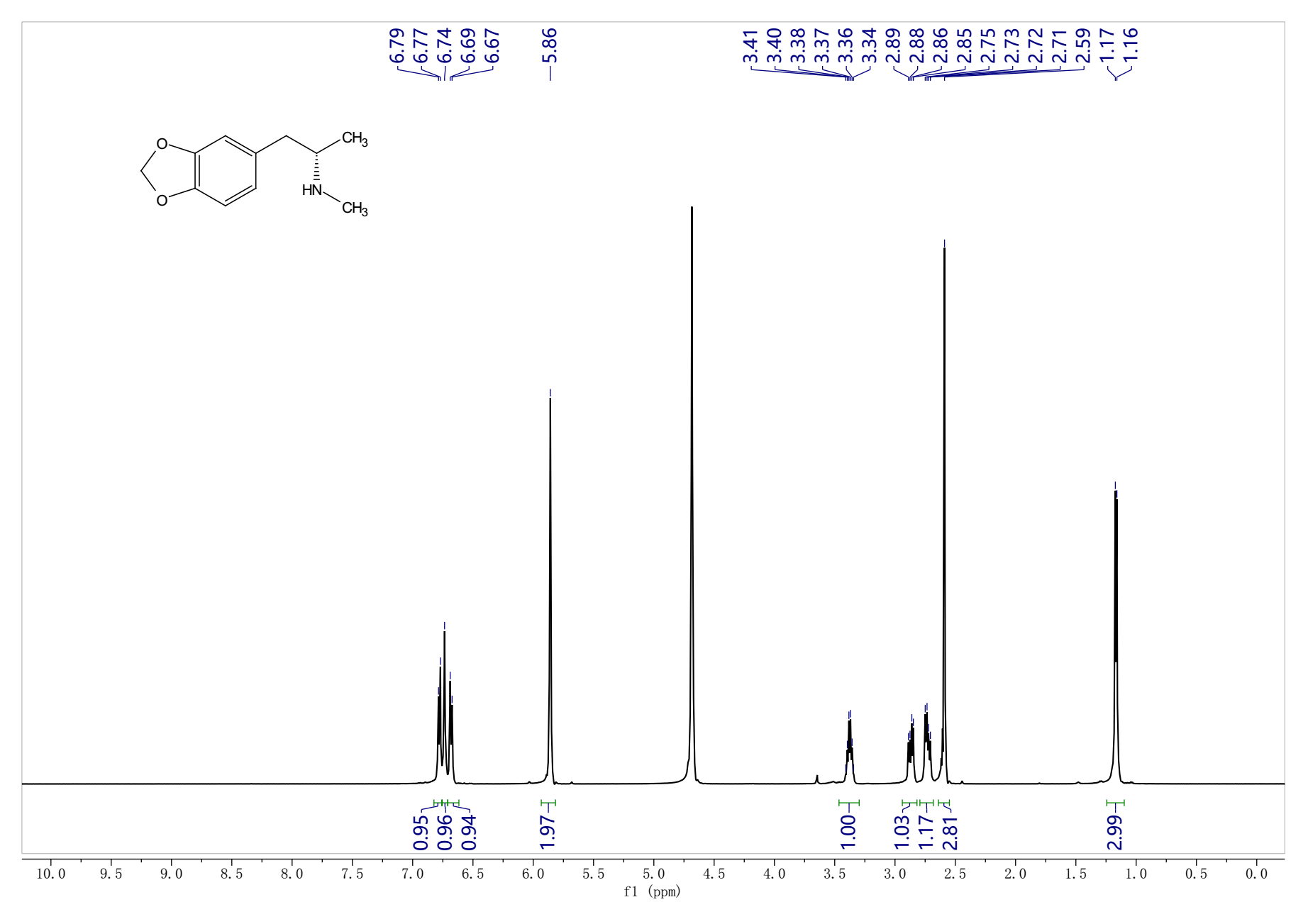


**Figure S30.** ^1^H NMR spectrum of ***S-*(+)-MDMA**


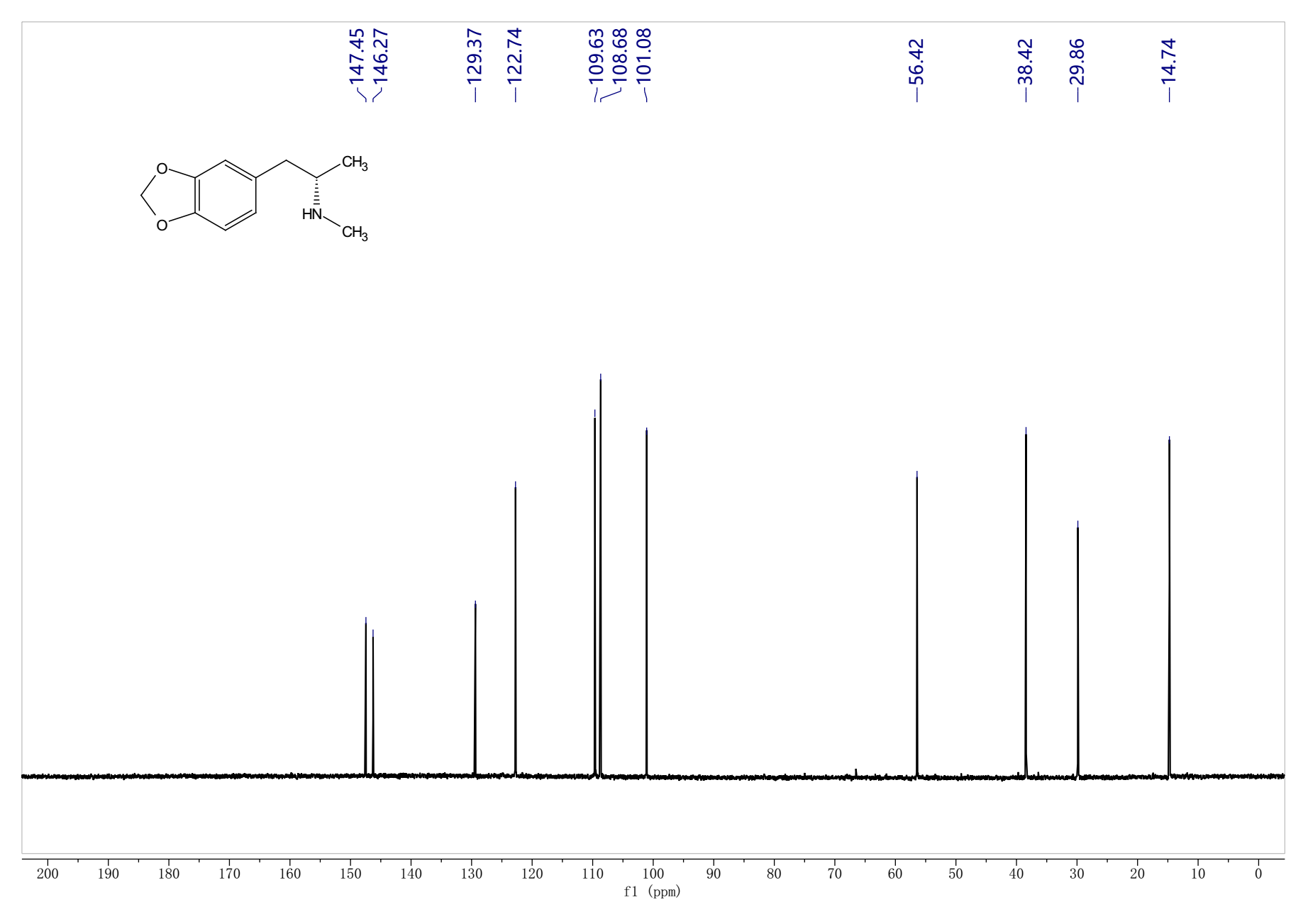


**Figure S31.** ^13^C NMR spectrum of ***S-*(+)-MDMA**


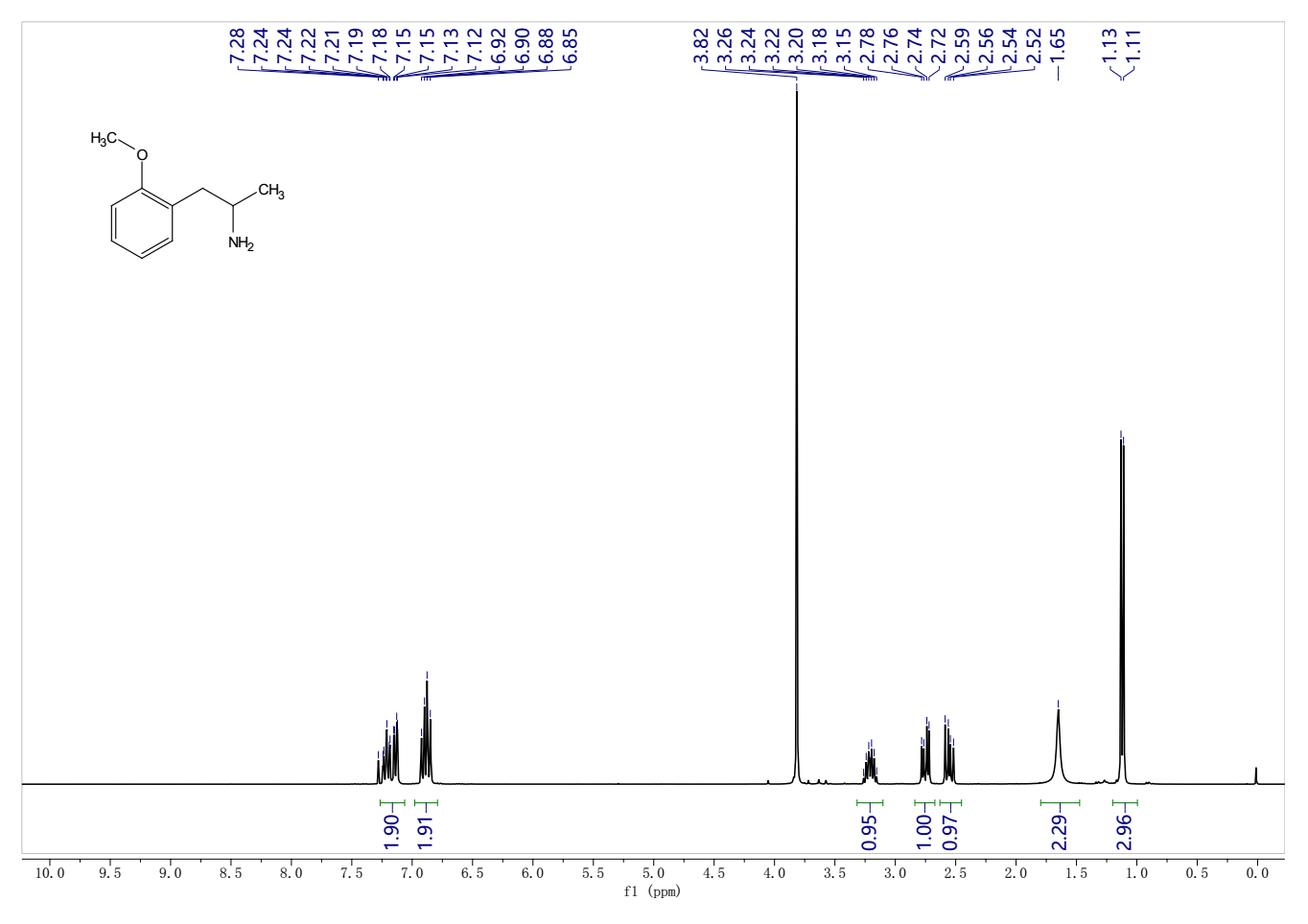


**Figure S32.** ^1^H NMR spectrum of **2-OMA**


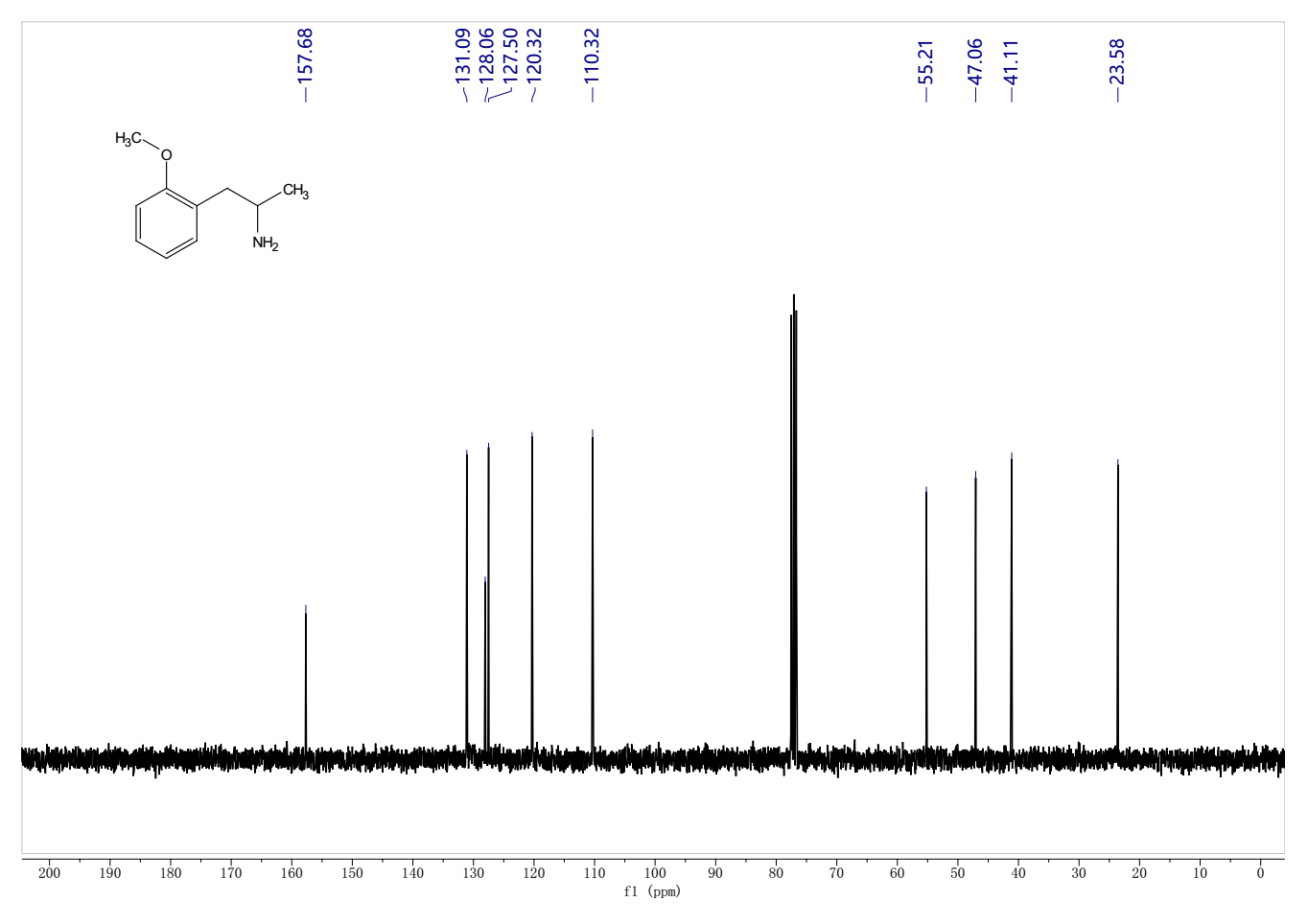


**Figure S33.** ^13^C NMR spectrum of **2-OMA**


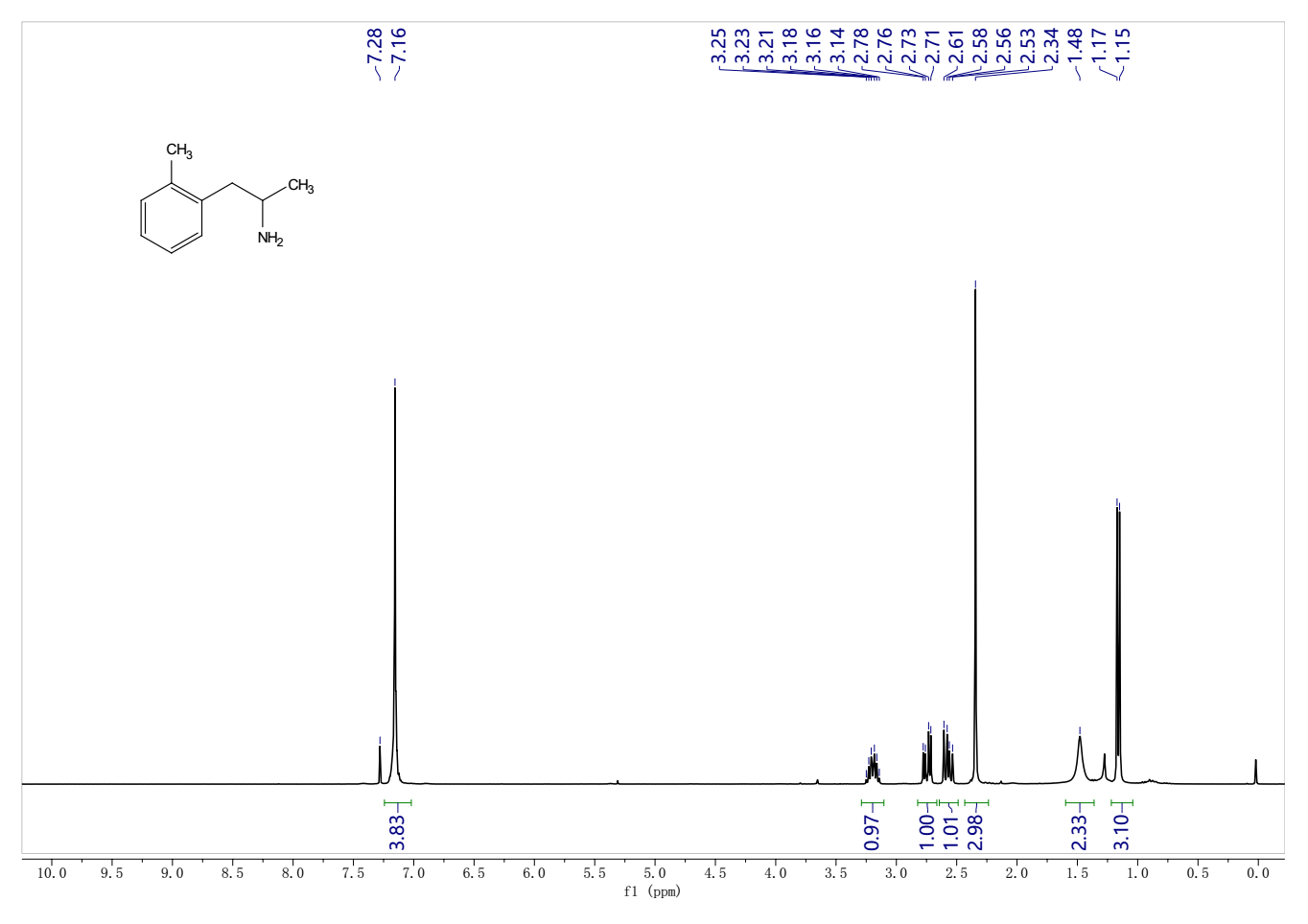


**Figure S34.** ^1^H NMR spectrum of **2-MA**


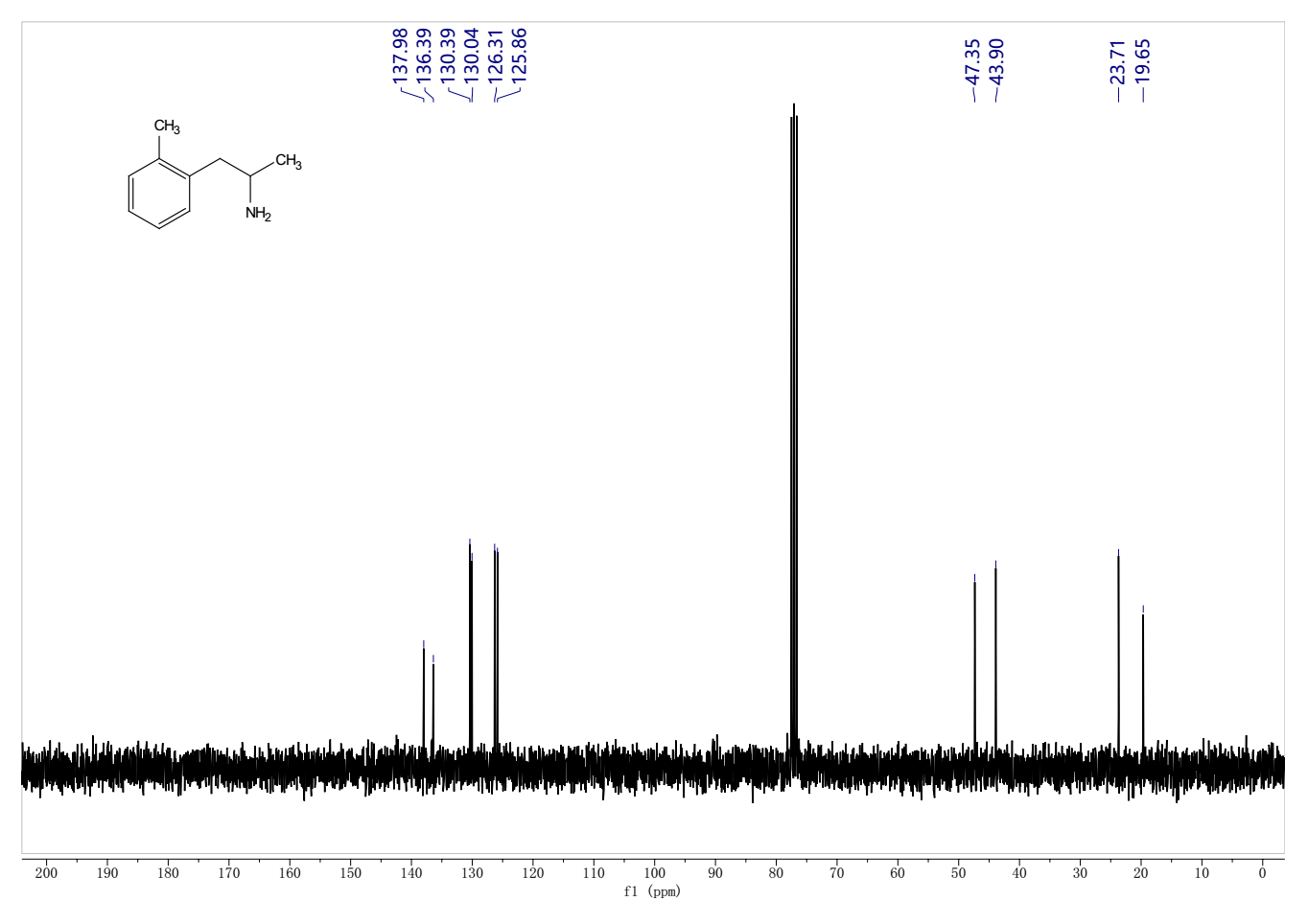


**Figure S35.** ^13^C NMR spectrum of **2-MA**


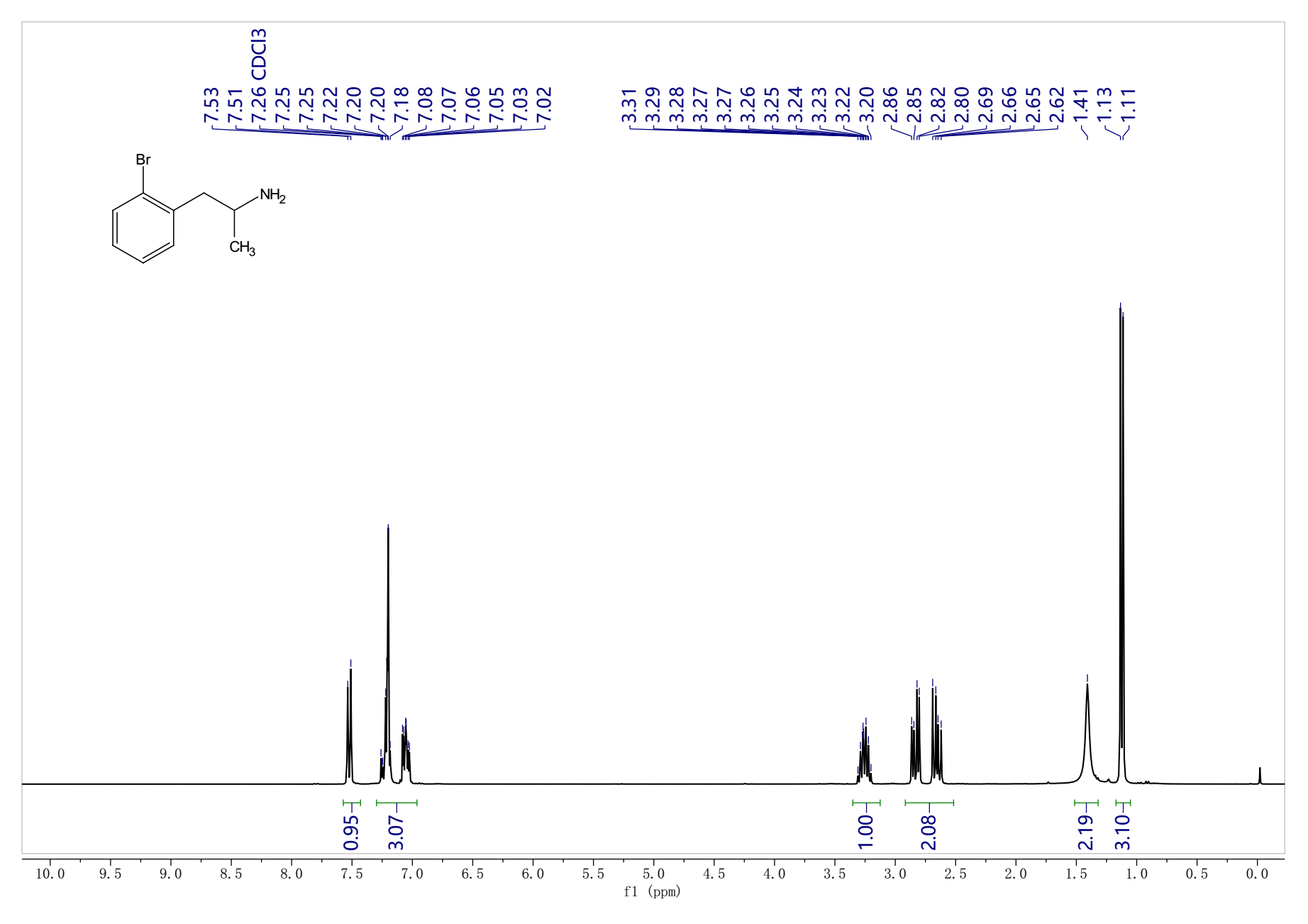


**Figure S36.** ^1^H NMR spectrum of **2-BA**


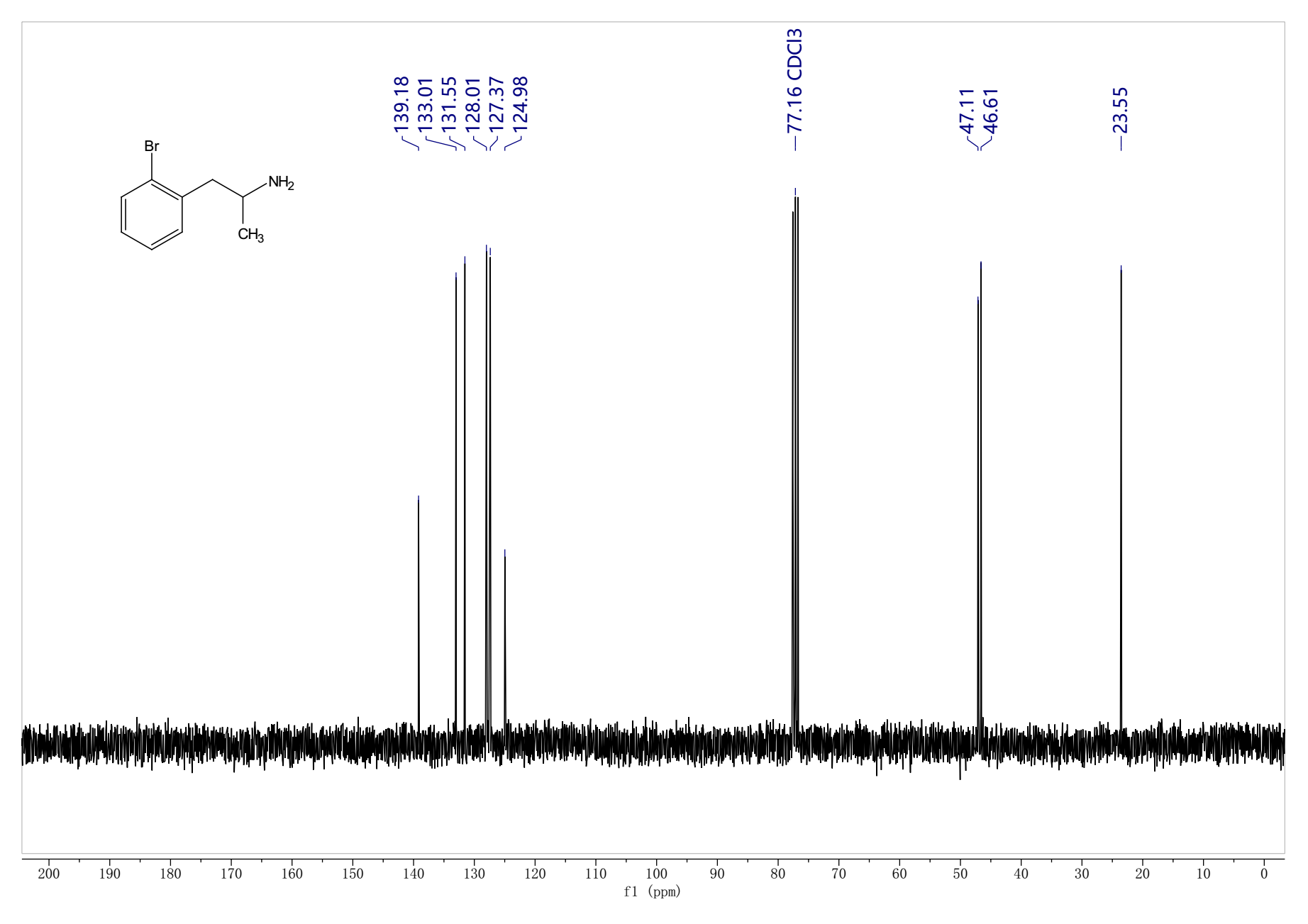


**Figure S37.** ^13^C NMR spectrum of **2-BA**


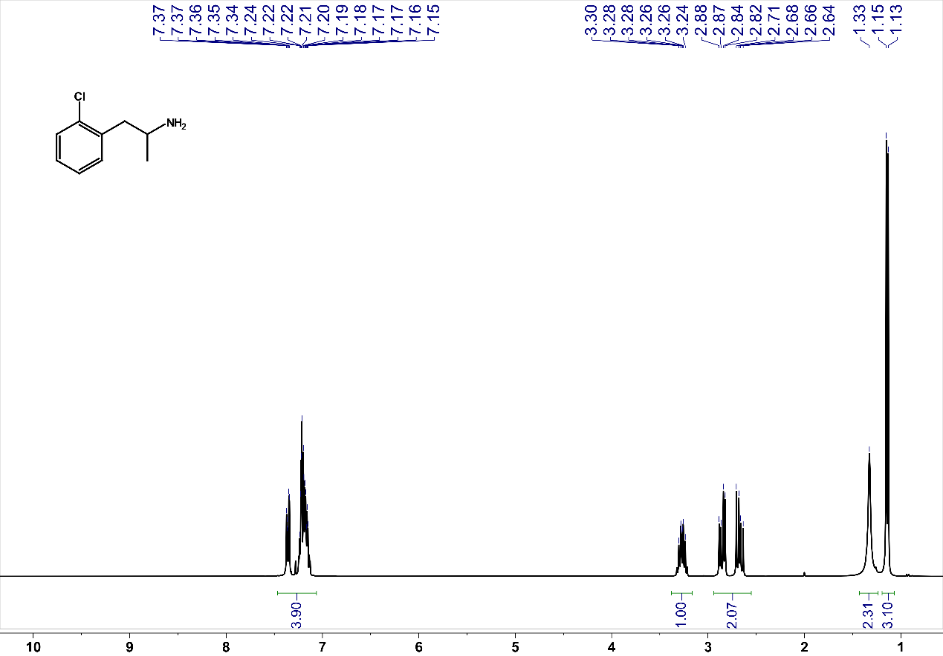


**Figure S38.** ^1^H NMR spectrum of **2-CA**


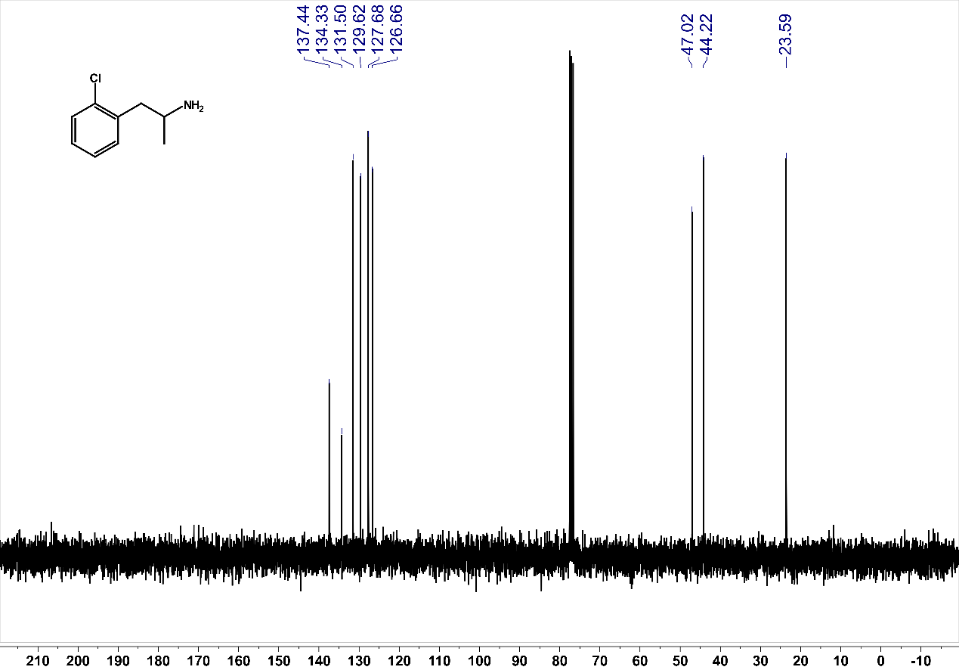


**Figure S39.** ^13^C NMR spectrum of **2-CA**


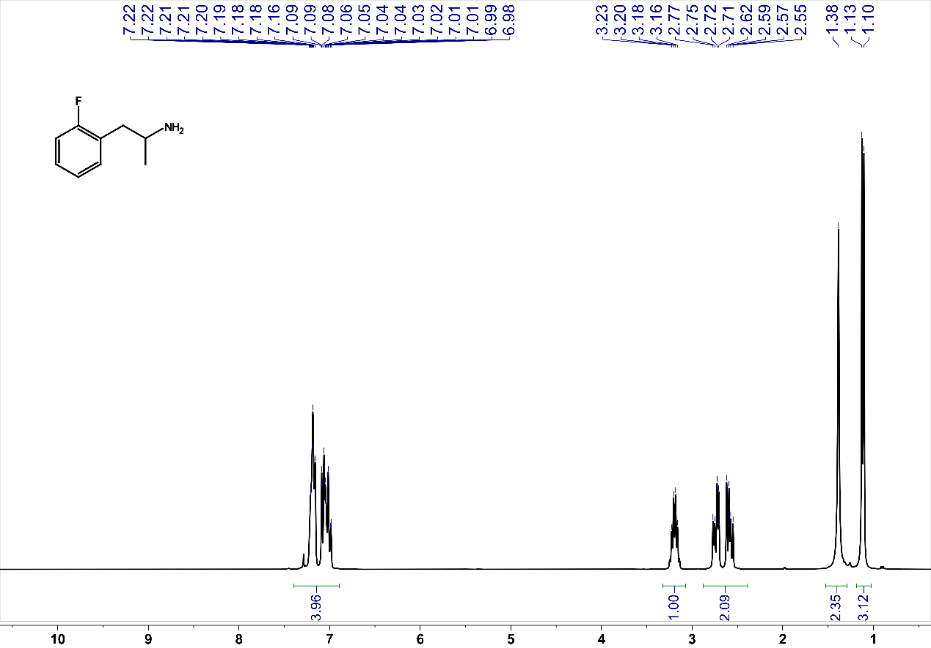


**Figure S40.** ^1^H NMR spectrum of **2-FA**


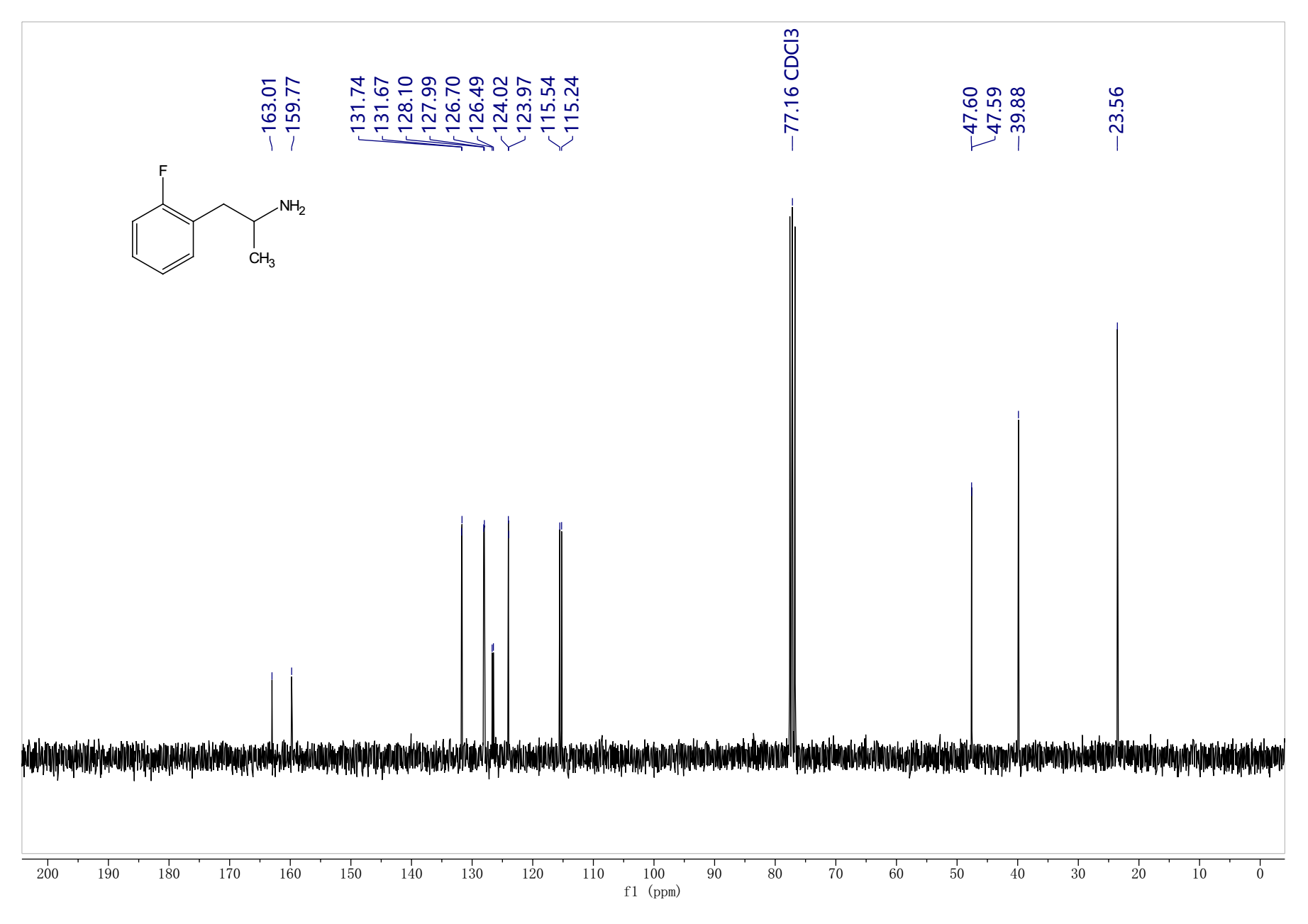


**Figure S41.** ^13^C NMR spectrum of **2-FA**


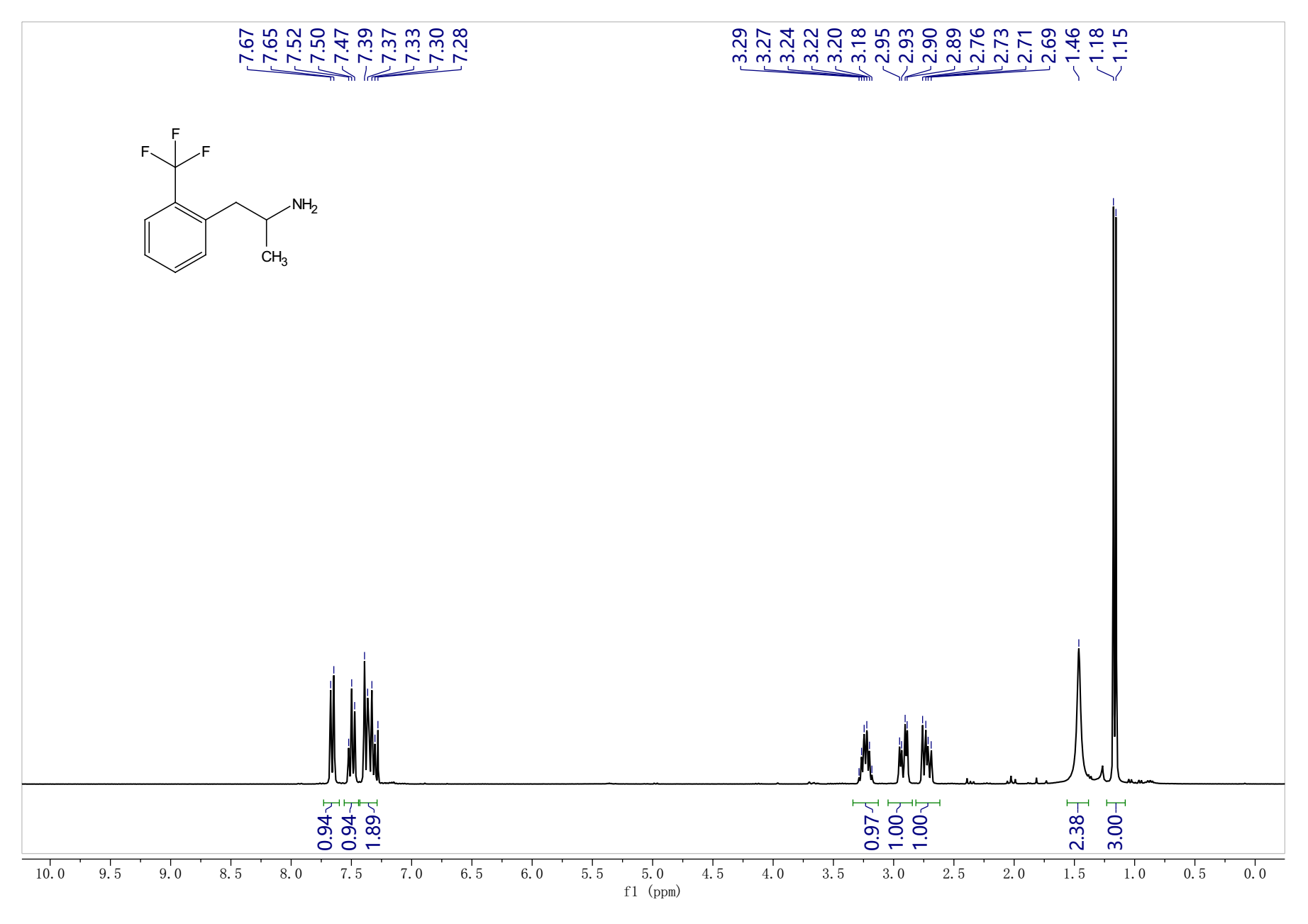


**Figure S42.** ^1^H NMR spectrum of **2-TFA**


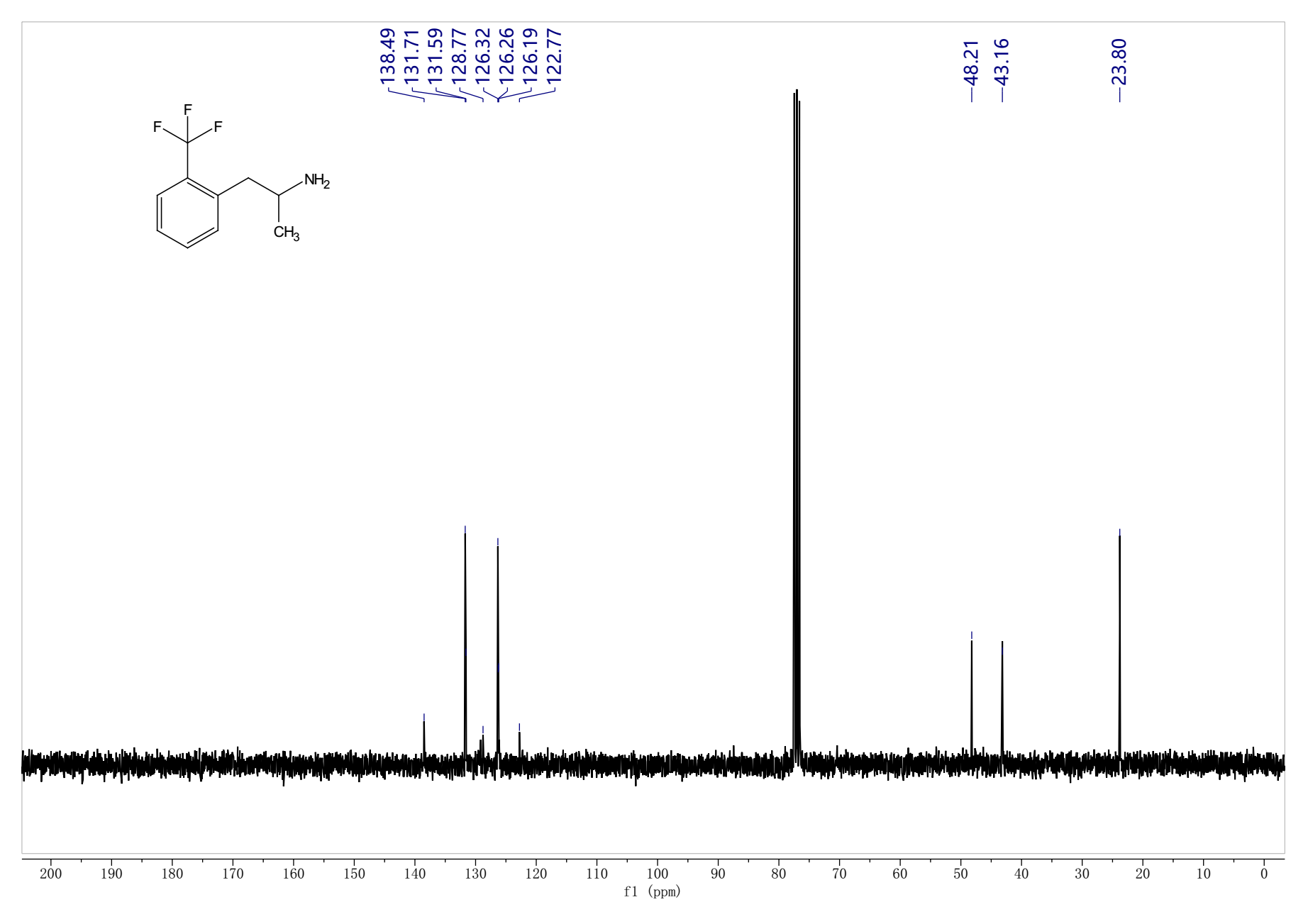


**Figure S43.** ^13^C NMR spectrum of **2-TFA**


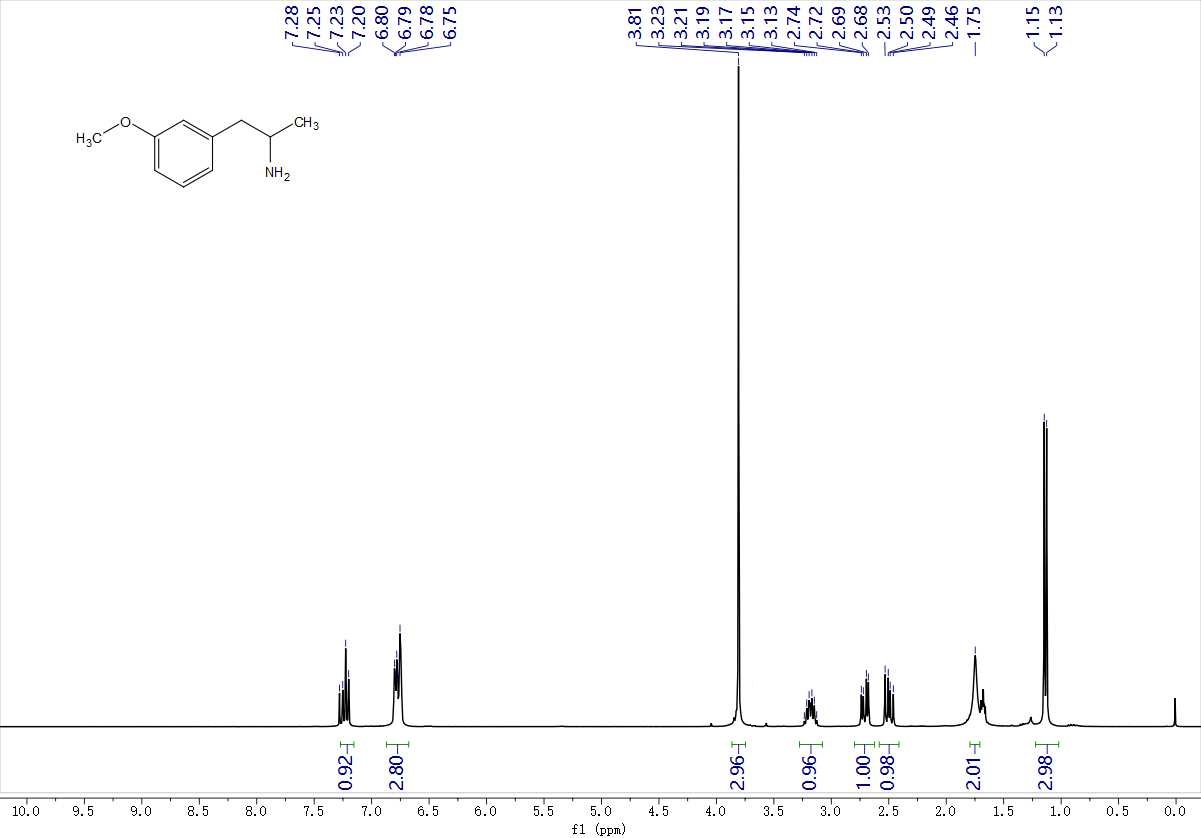


**Figure S44.** ^1^H NMR spectrum of **3-OMA**


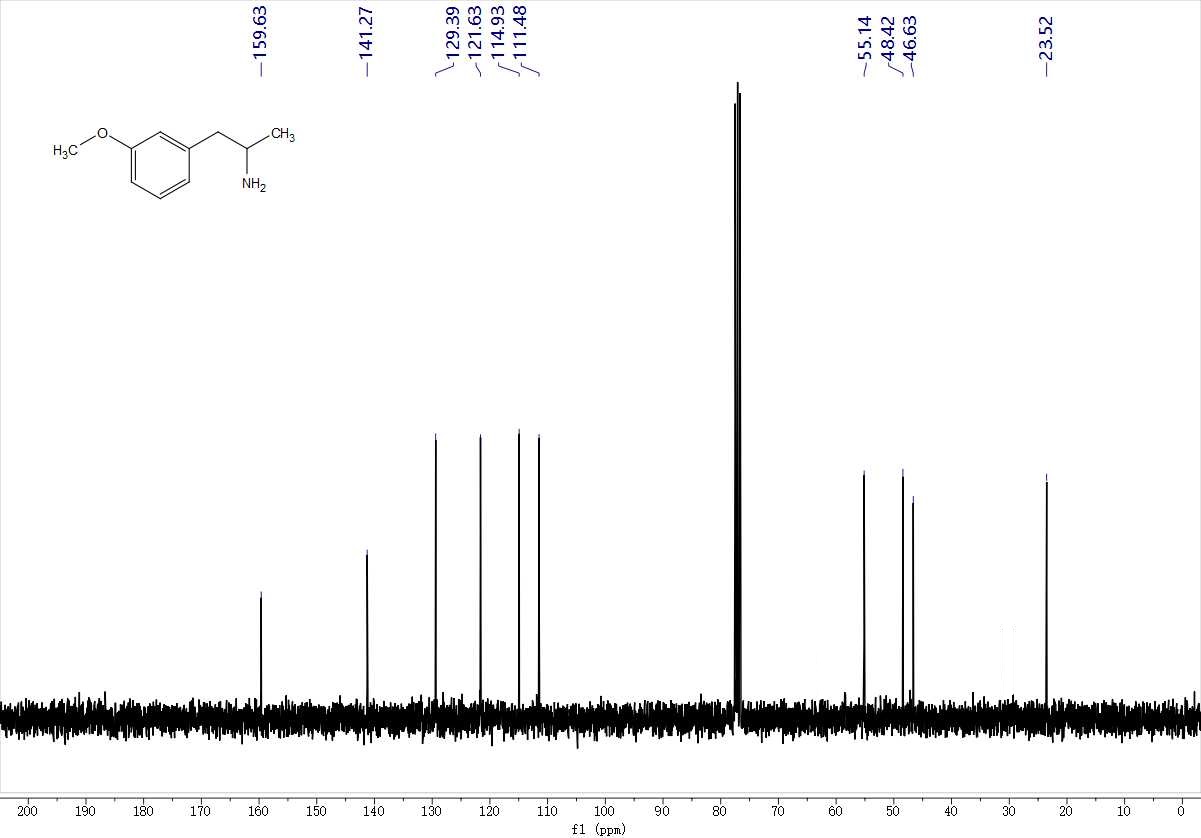


**Figure S45.** ^13^C NMR spectrum of **3-OMA**


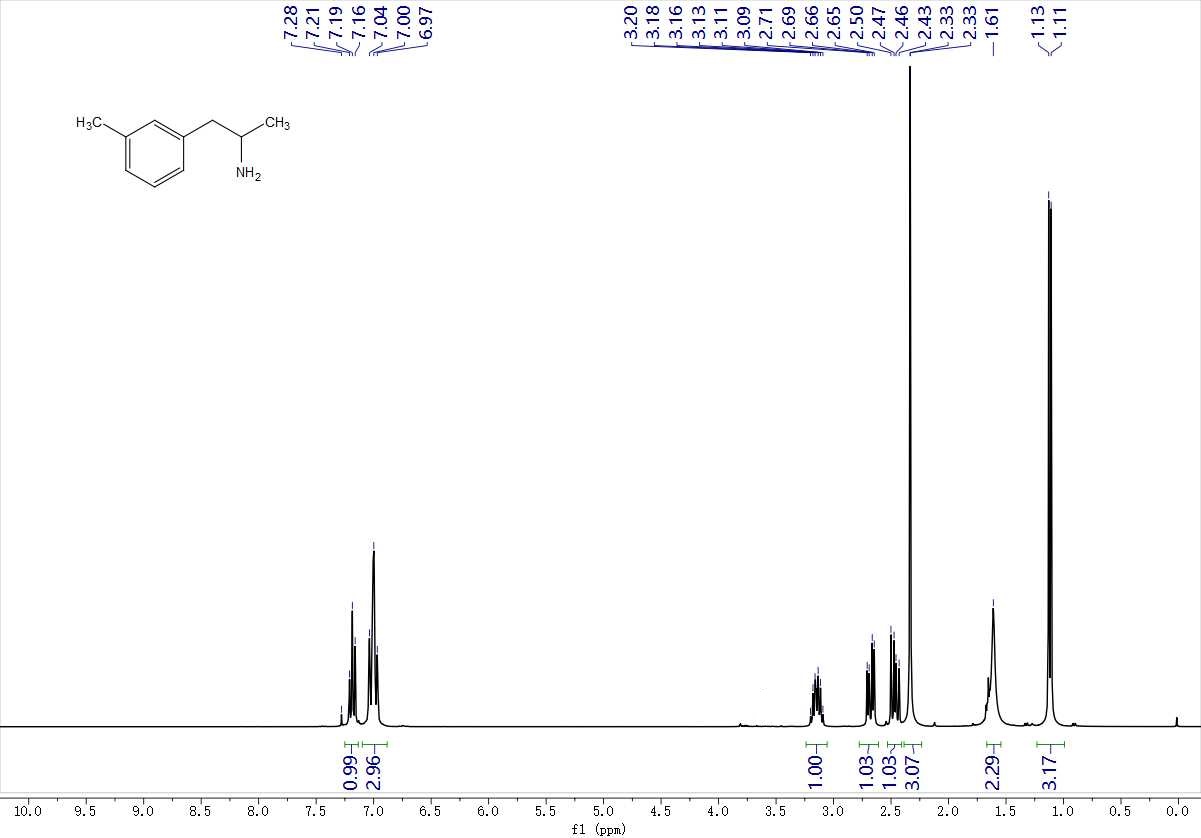


**Figure S46.** ^1^H NMR spectrum of **3-MA**


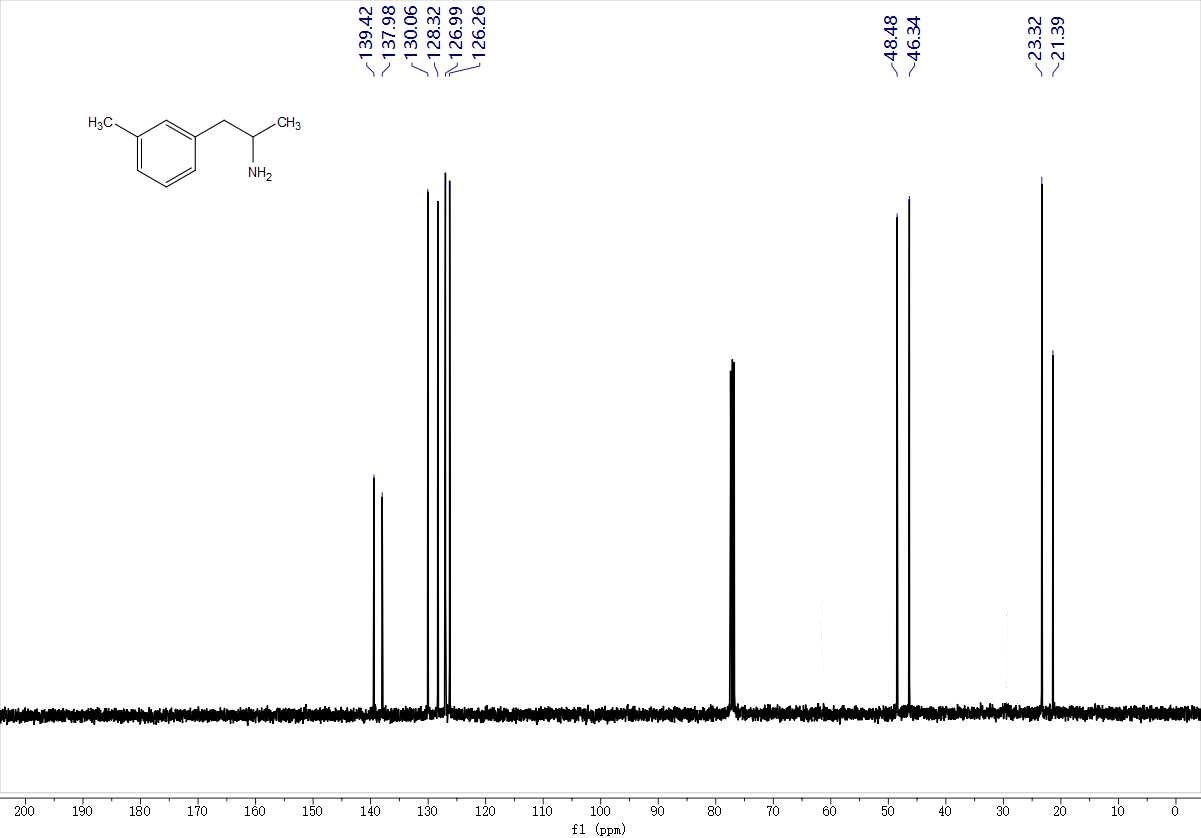


**Figure S47.** ^13^C NMR spectrum of **3-MA**


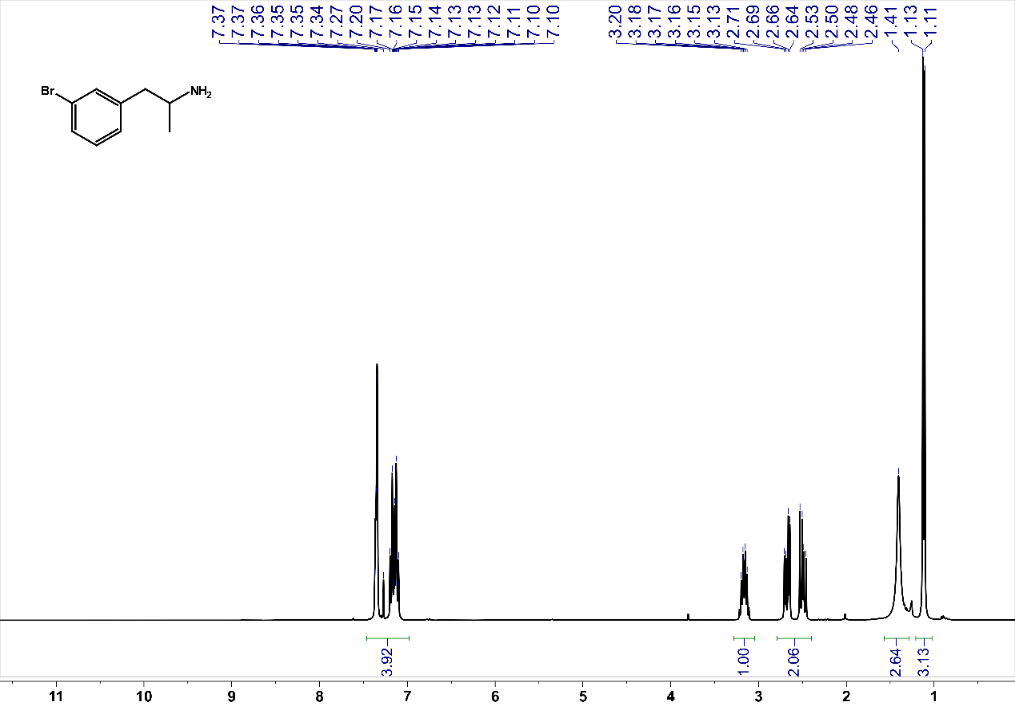


**Figure S48.** ^1^H NMR spectrum of **3-BA**


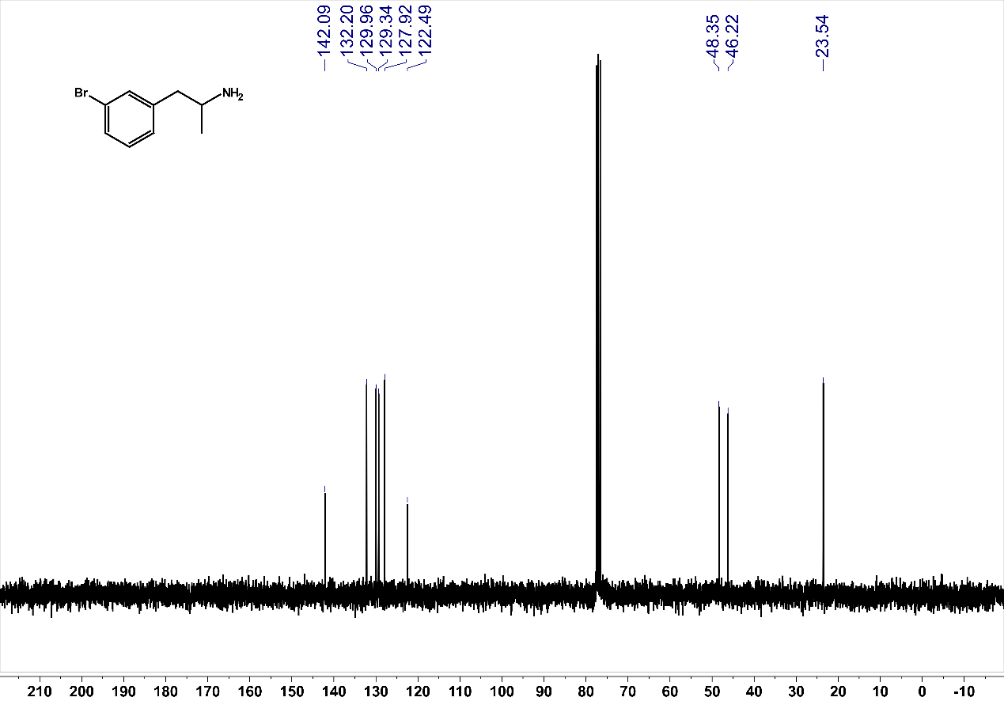


**Figure S49.** ^13^C NMR spectrum of **3-BA**


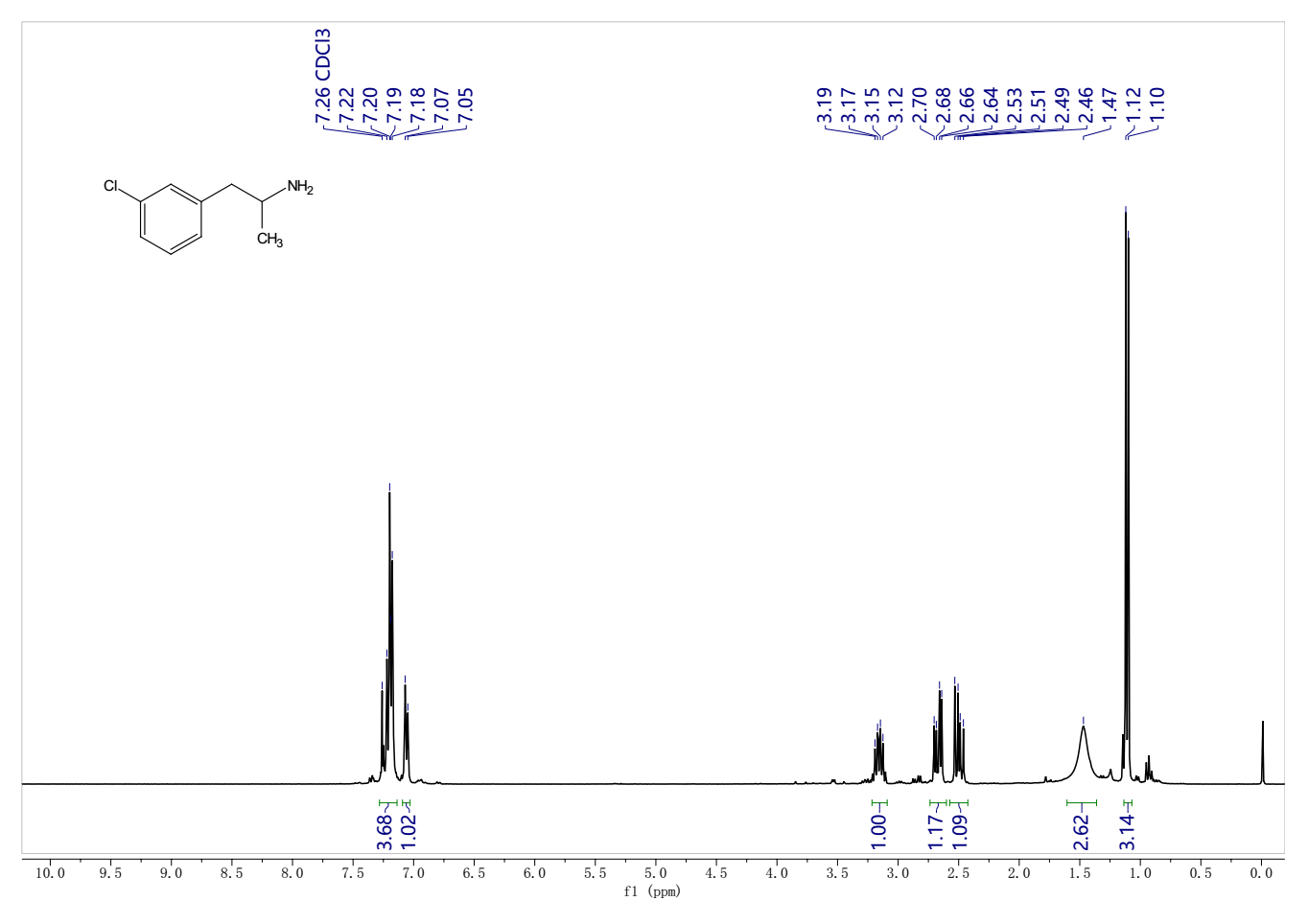


**Figure S50.** ^1^H NMR spectrum of **3-CA**


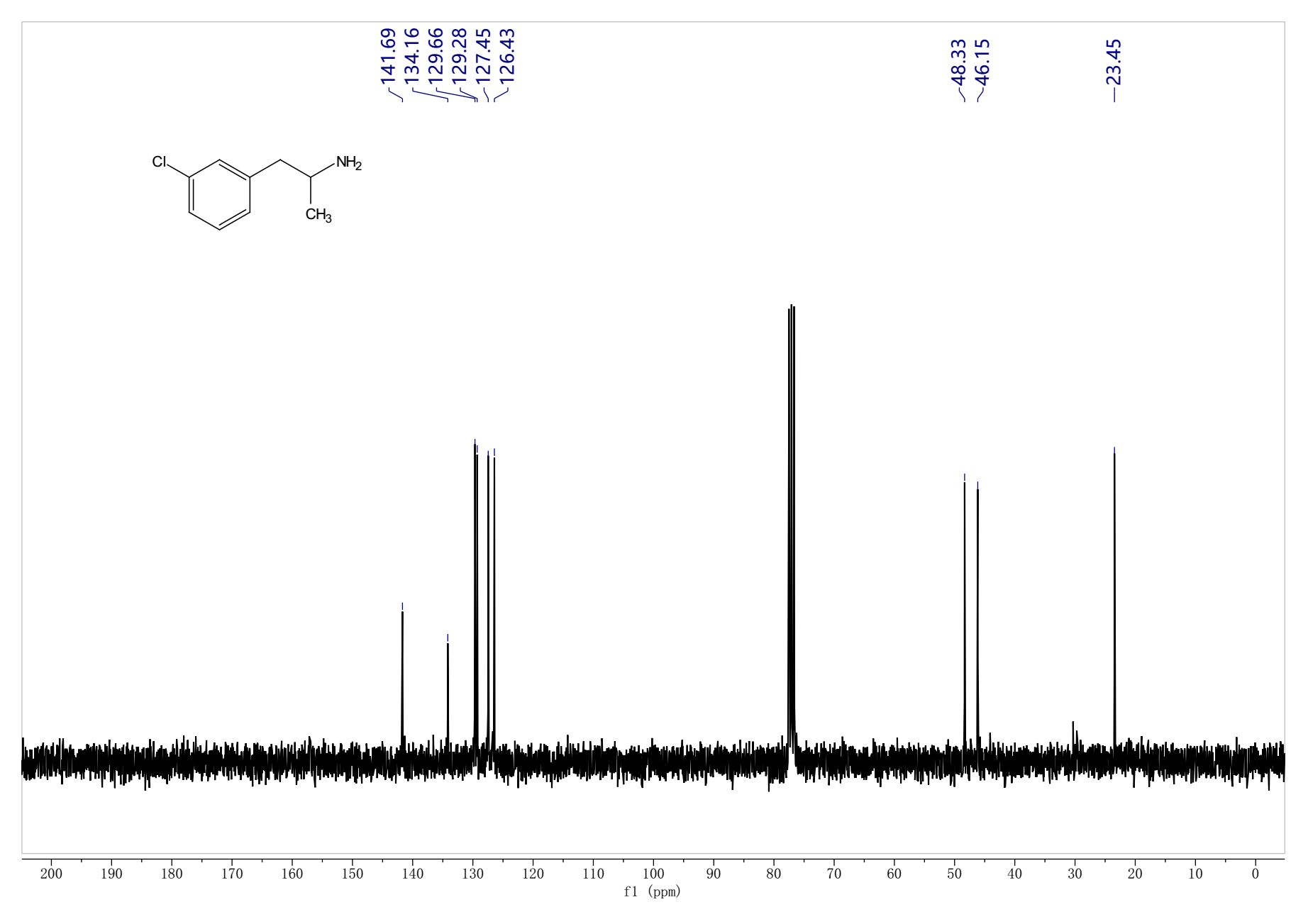


**Figure S51.** ^13^C NMR spectrum of **3-CA**


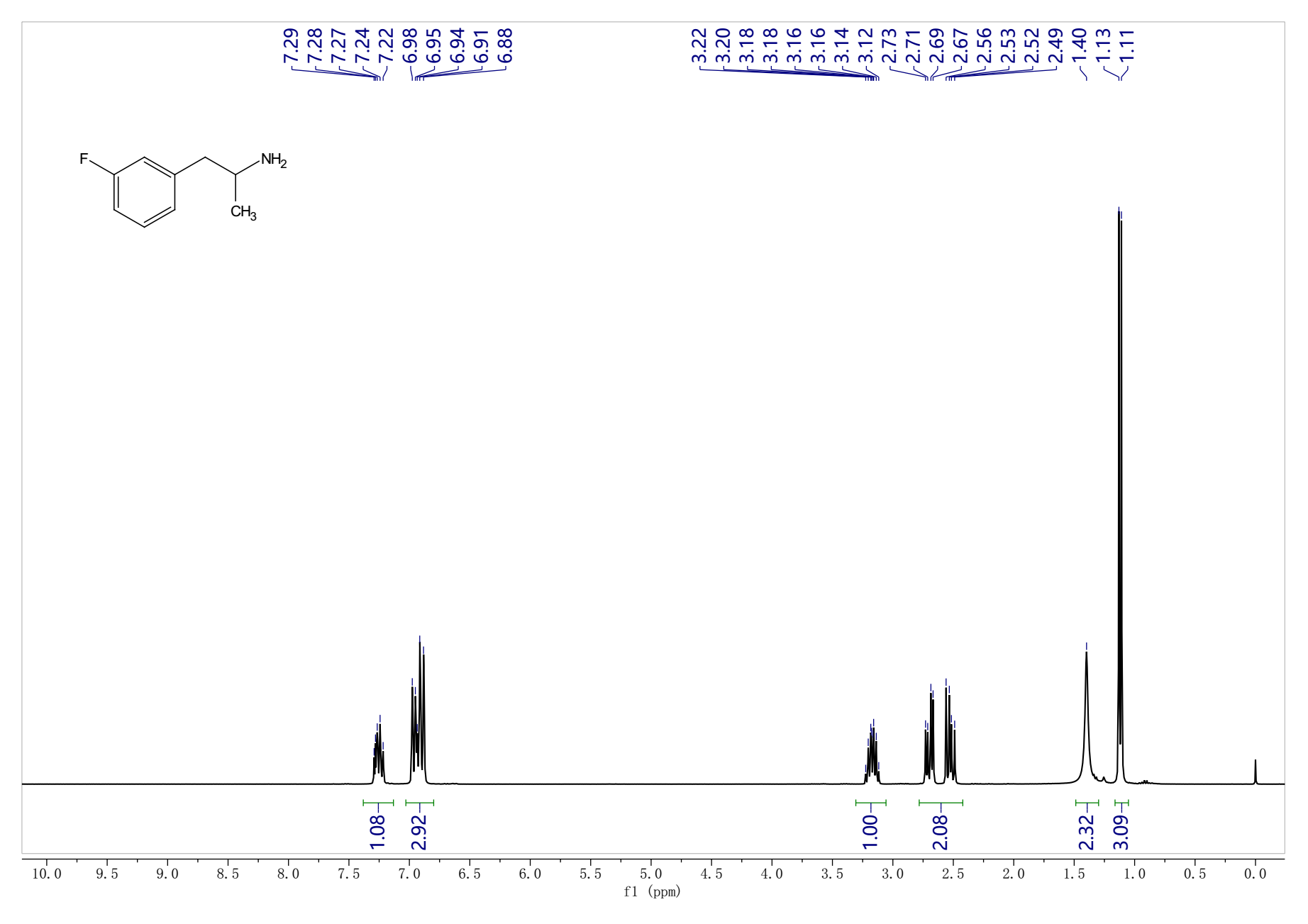


**Figure S52.** ^1^H NMR spectrum of **3-FA**


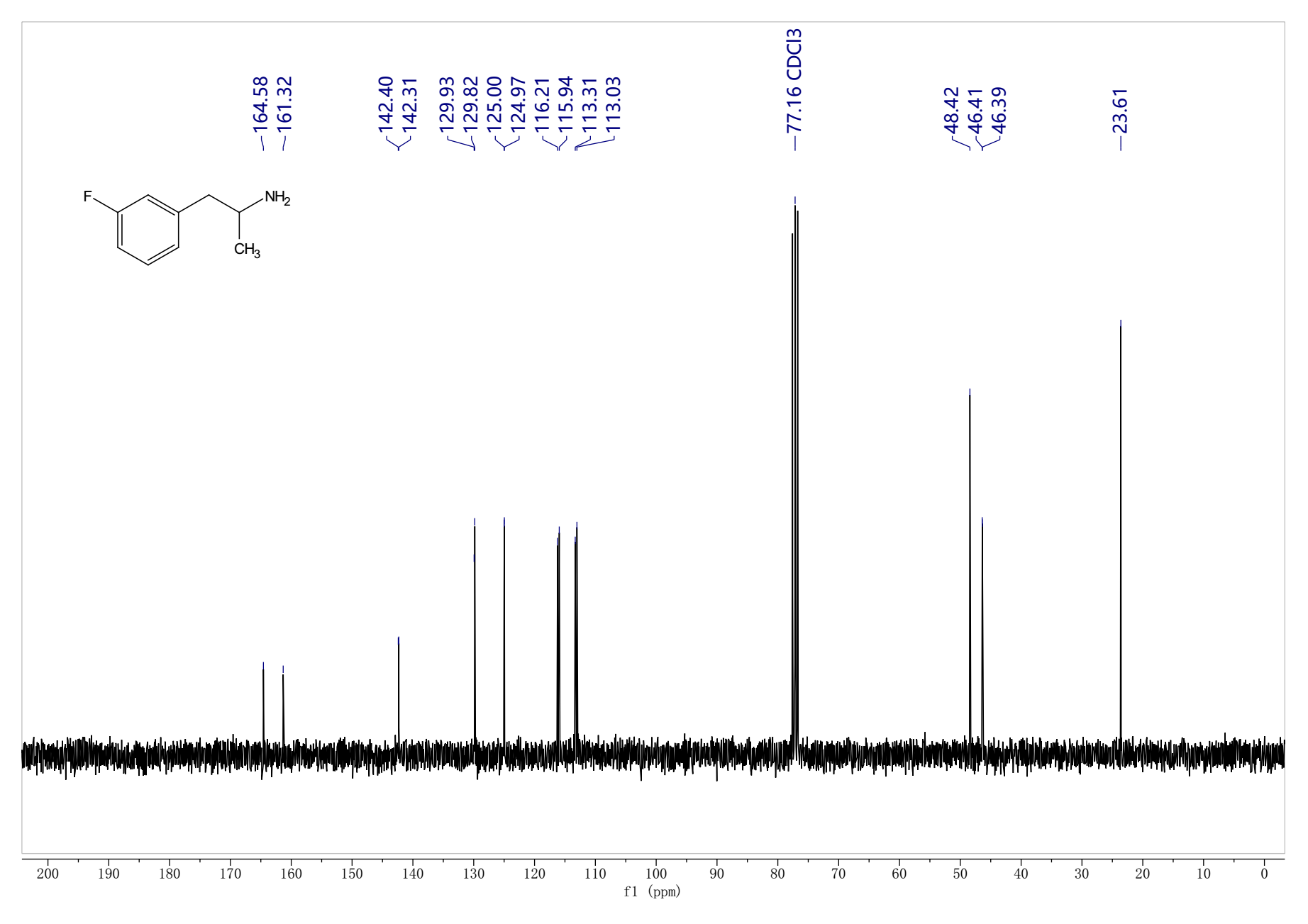


**Figure S53.** ^13^C NMR spectrum of **3-FA**


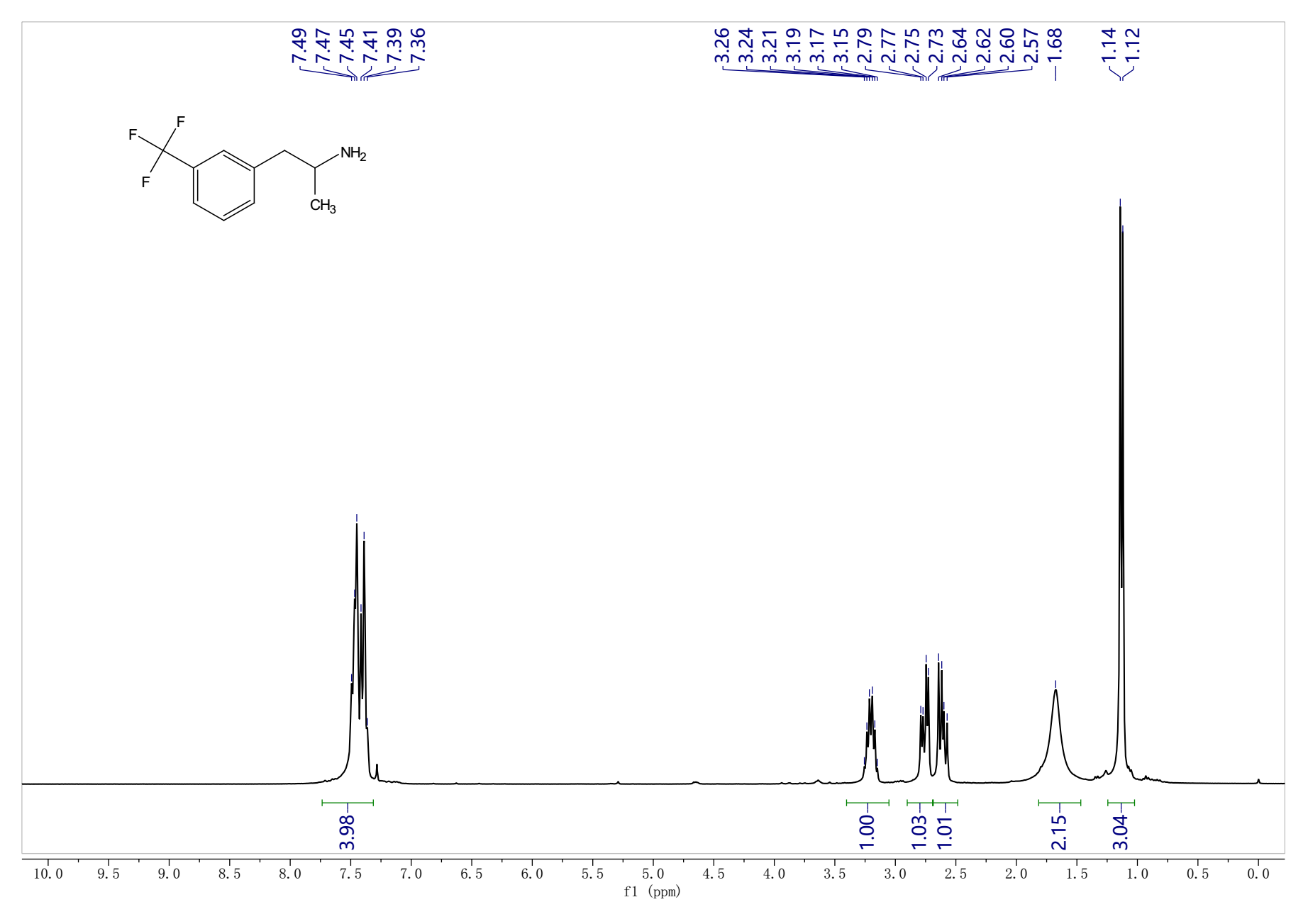


**Figure S54.** ^1^H NMR spectrum of **3-TFA**


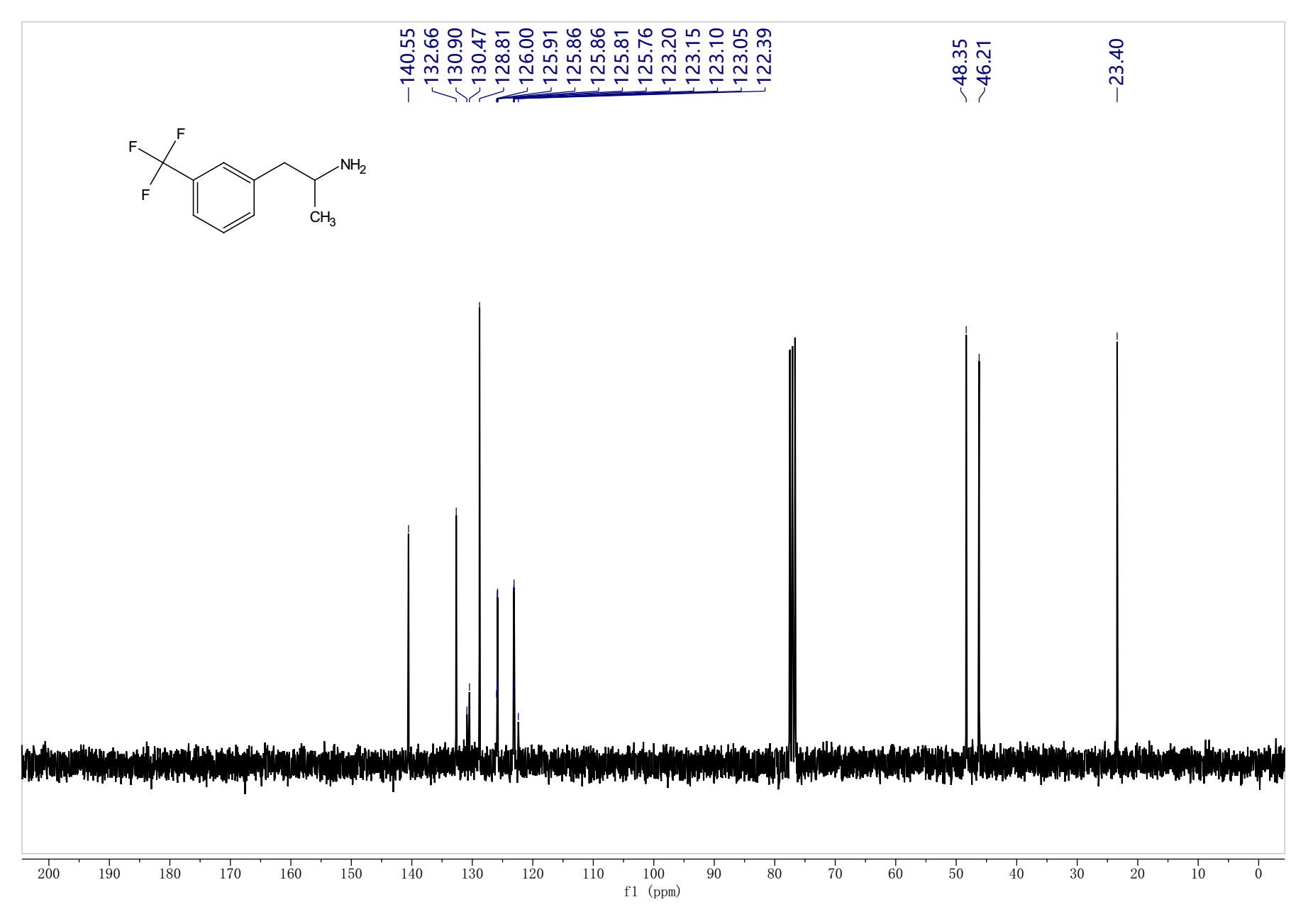


**Figure S55.** ^13^C NMR spectrum of **3-TFA**


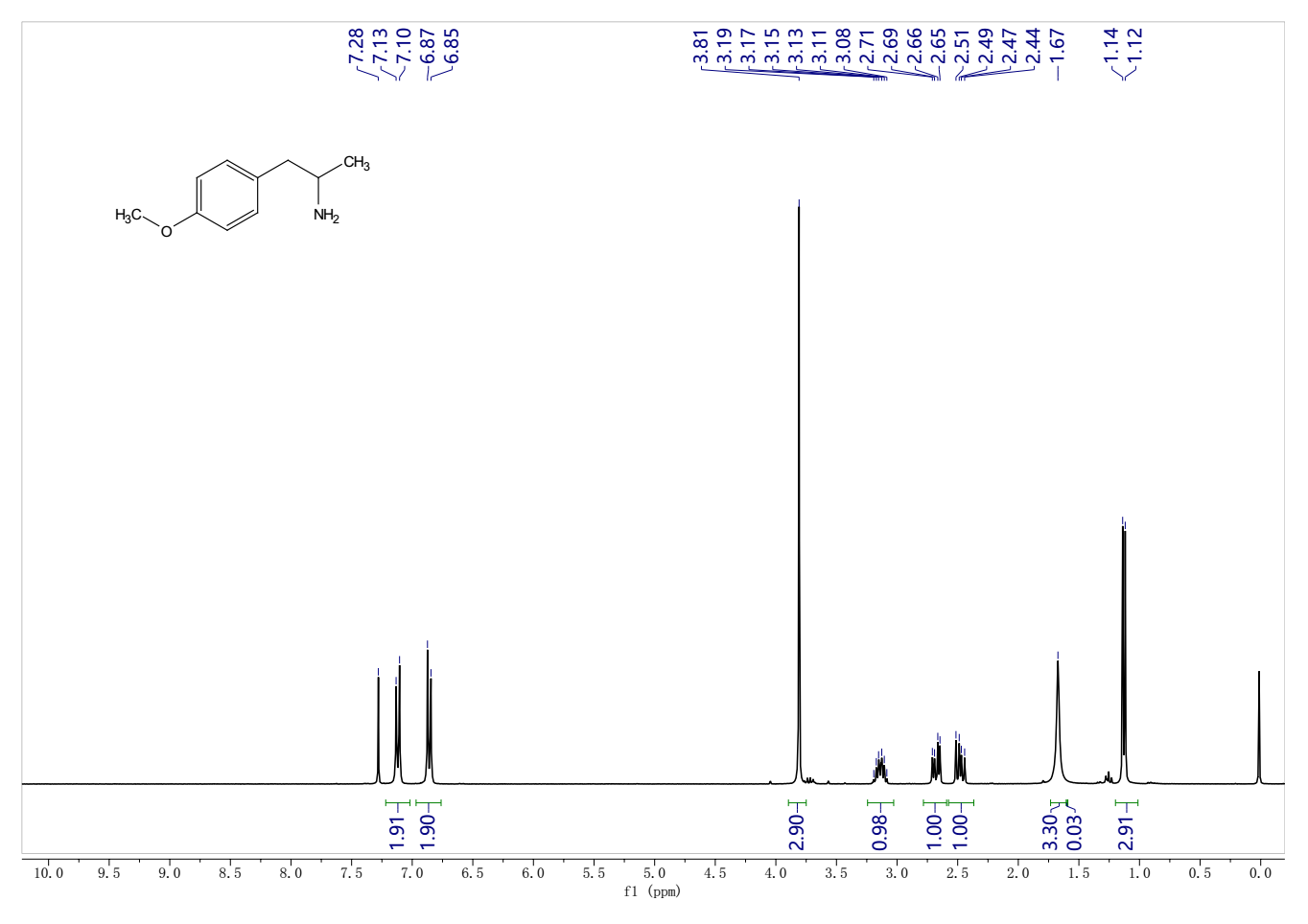


**Figure S56.** ^1^H NMR spectrum of **4-OMA**


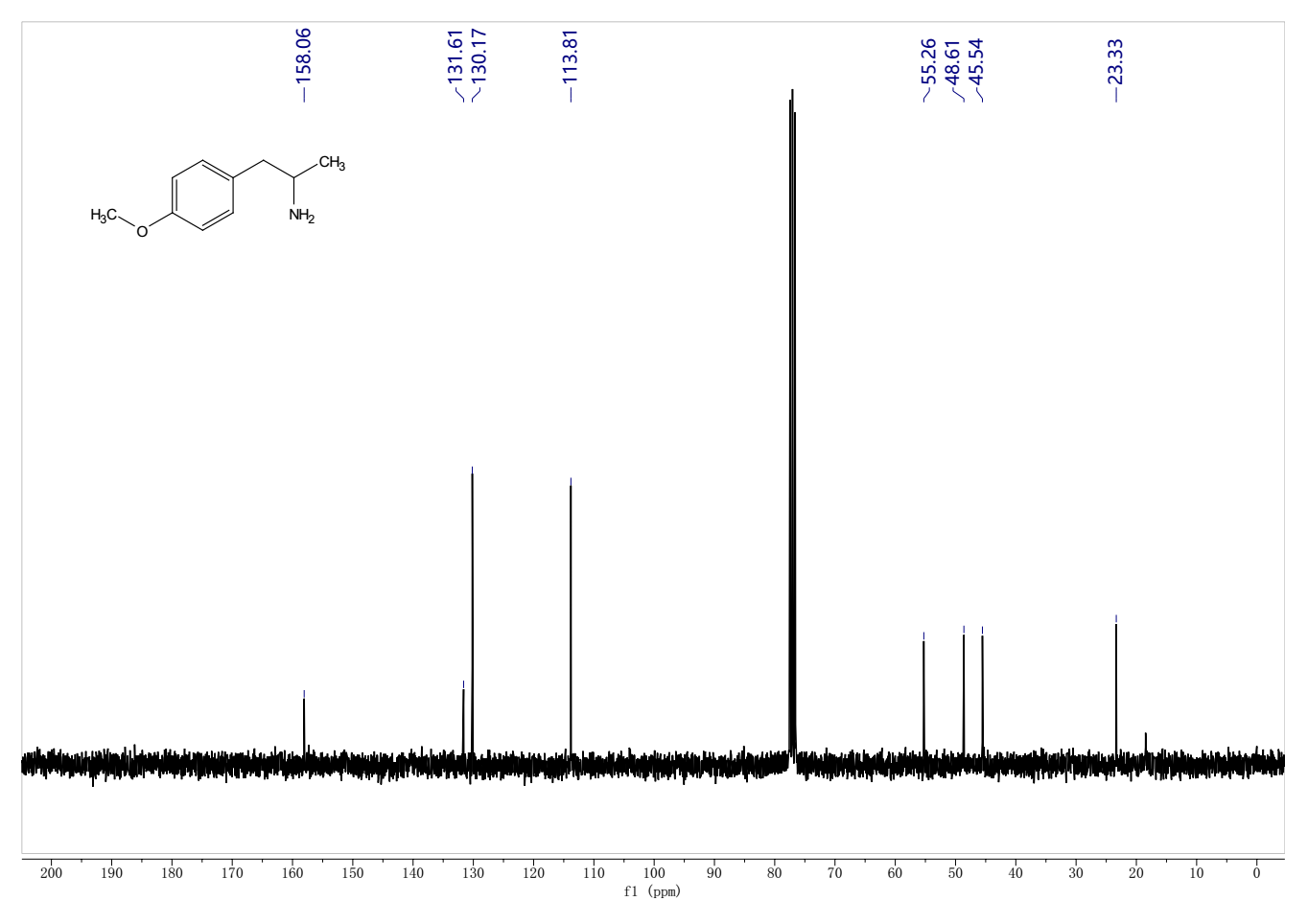


**Figure S57.** ^13^C NMR spectrum of **4-OMA**


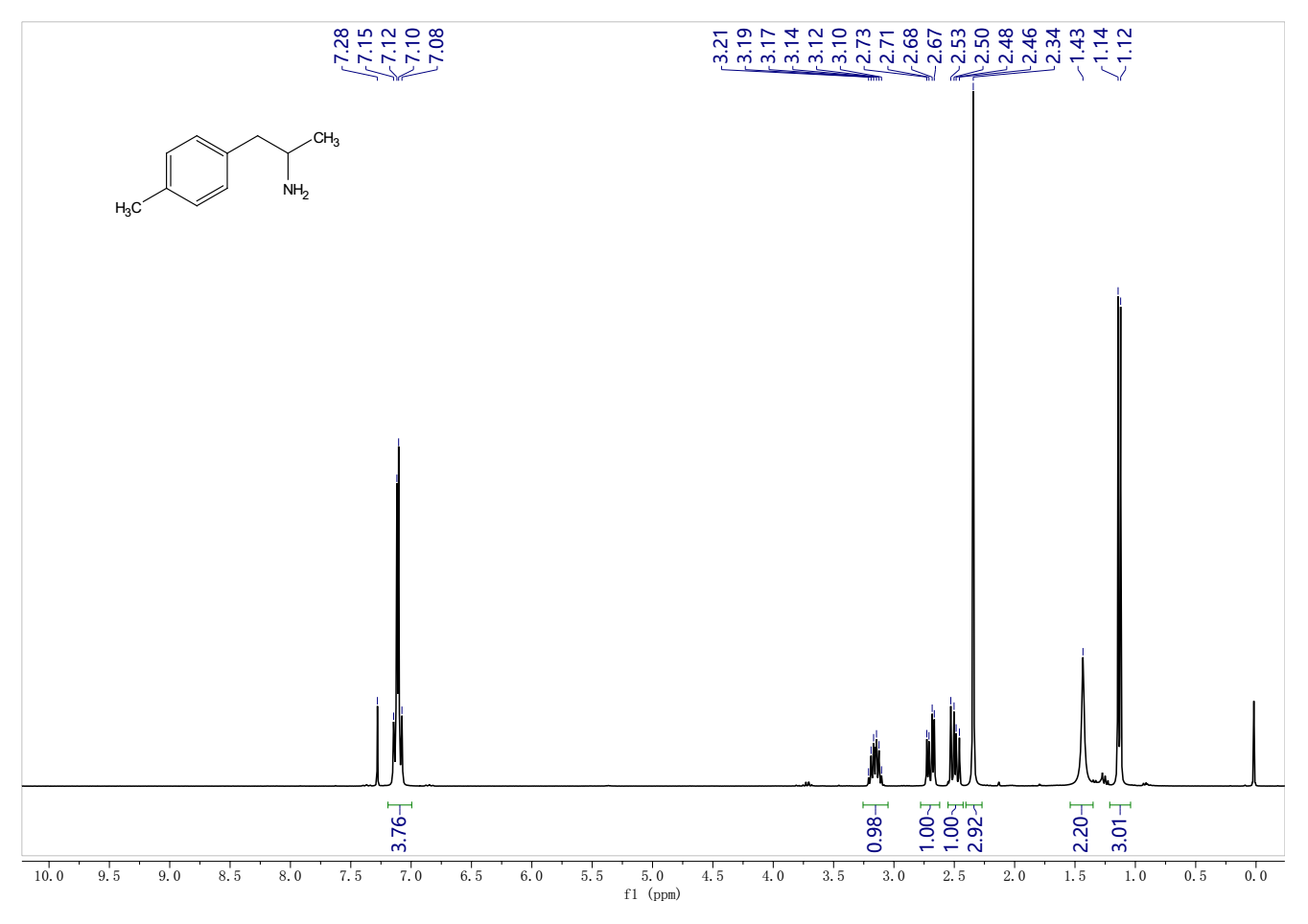


**Figure S58.** ^1^H NMR spectrum of **4-MA**


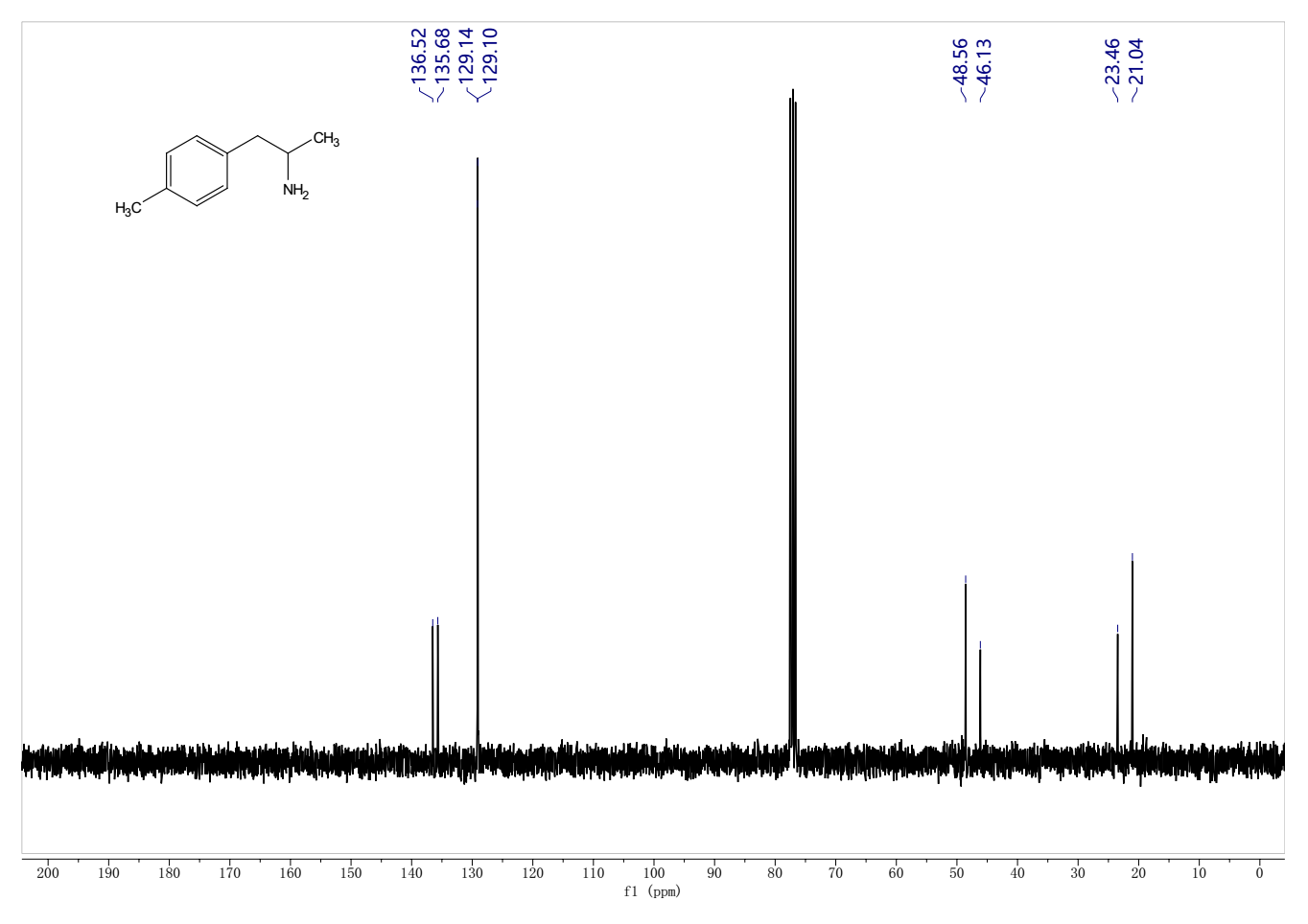


**Figure S59.** ^13^C NMR spectrum of **4-MA**


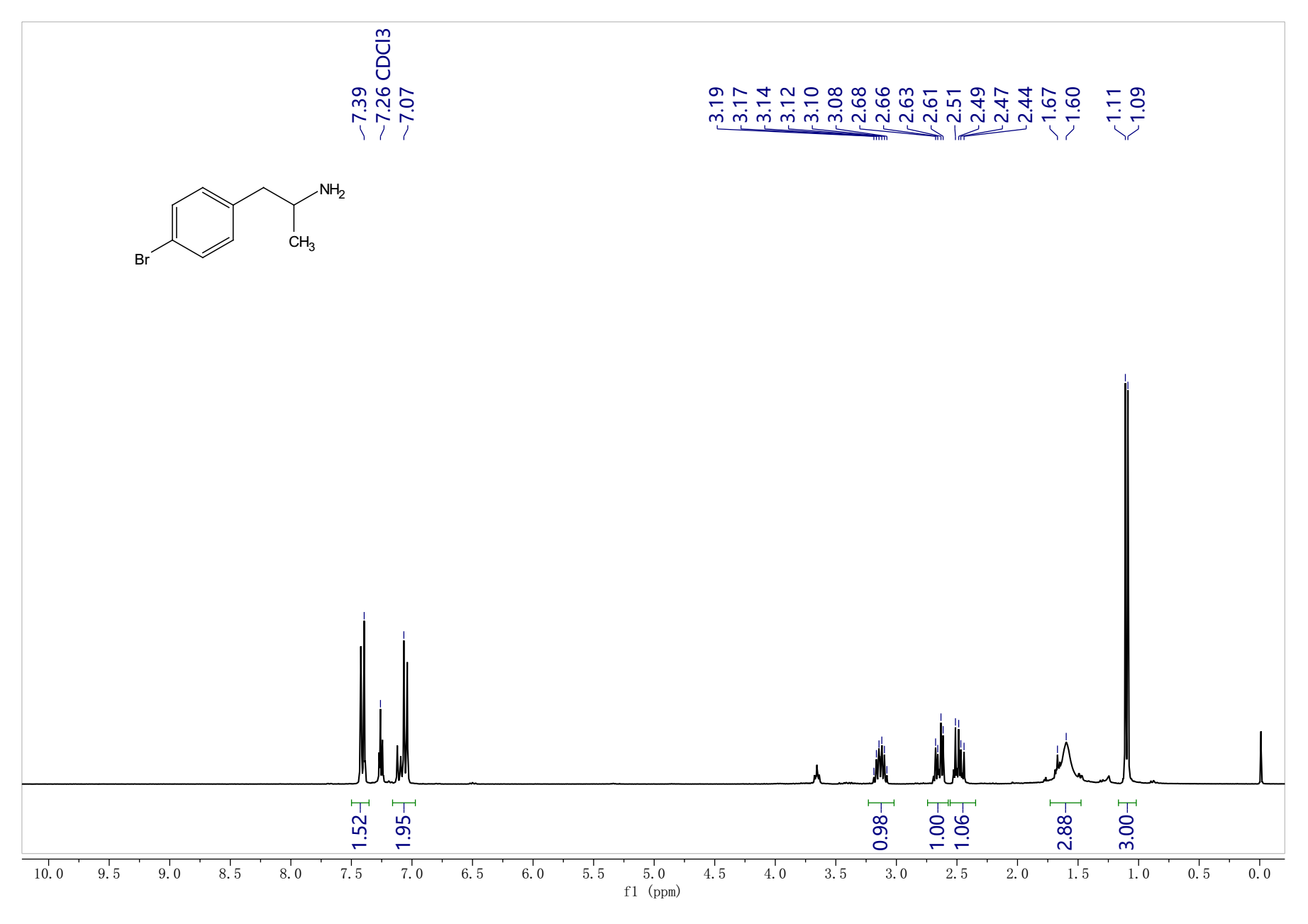


**Figure S60.** ^1^H NMR spectrum of **4-BA**


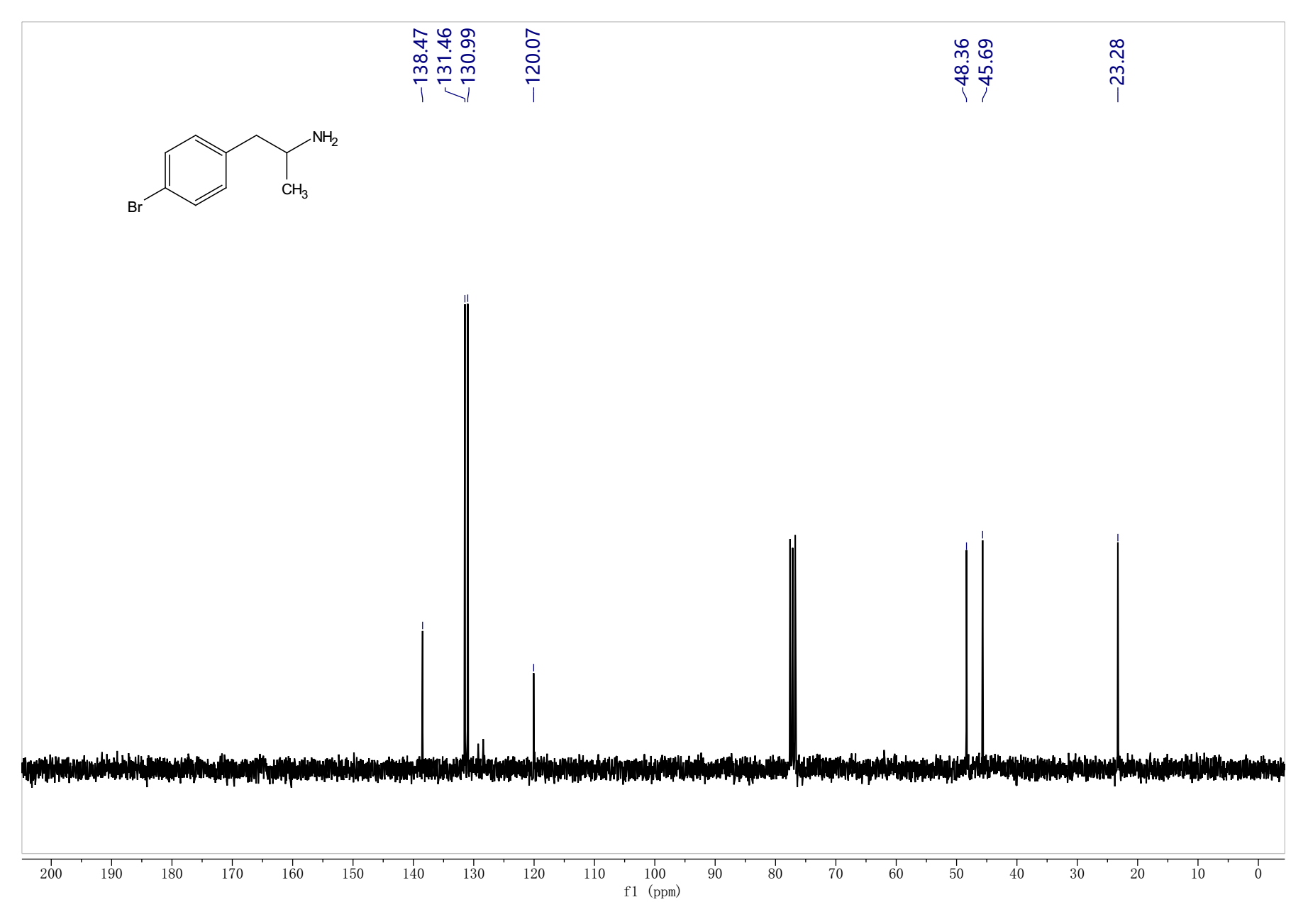


**Figure S61.** ^13^C NMR spectrum of **4-BA**


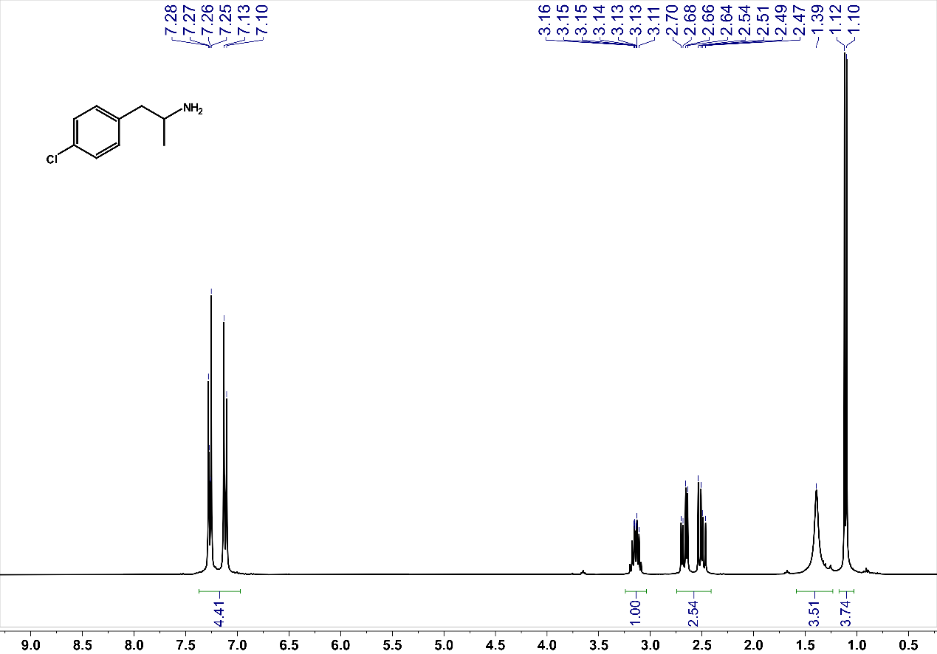


**Figure S62.** ^1^H NMR spectrum of **4-CA**


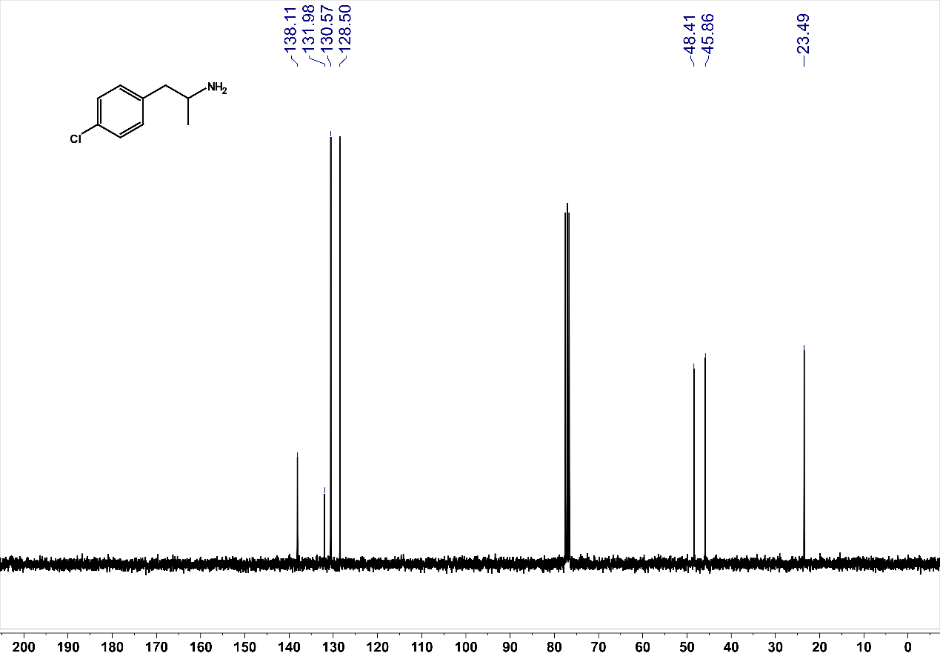


**Figure S63.** ^13^C NMR spectrum of **4-CA**


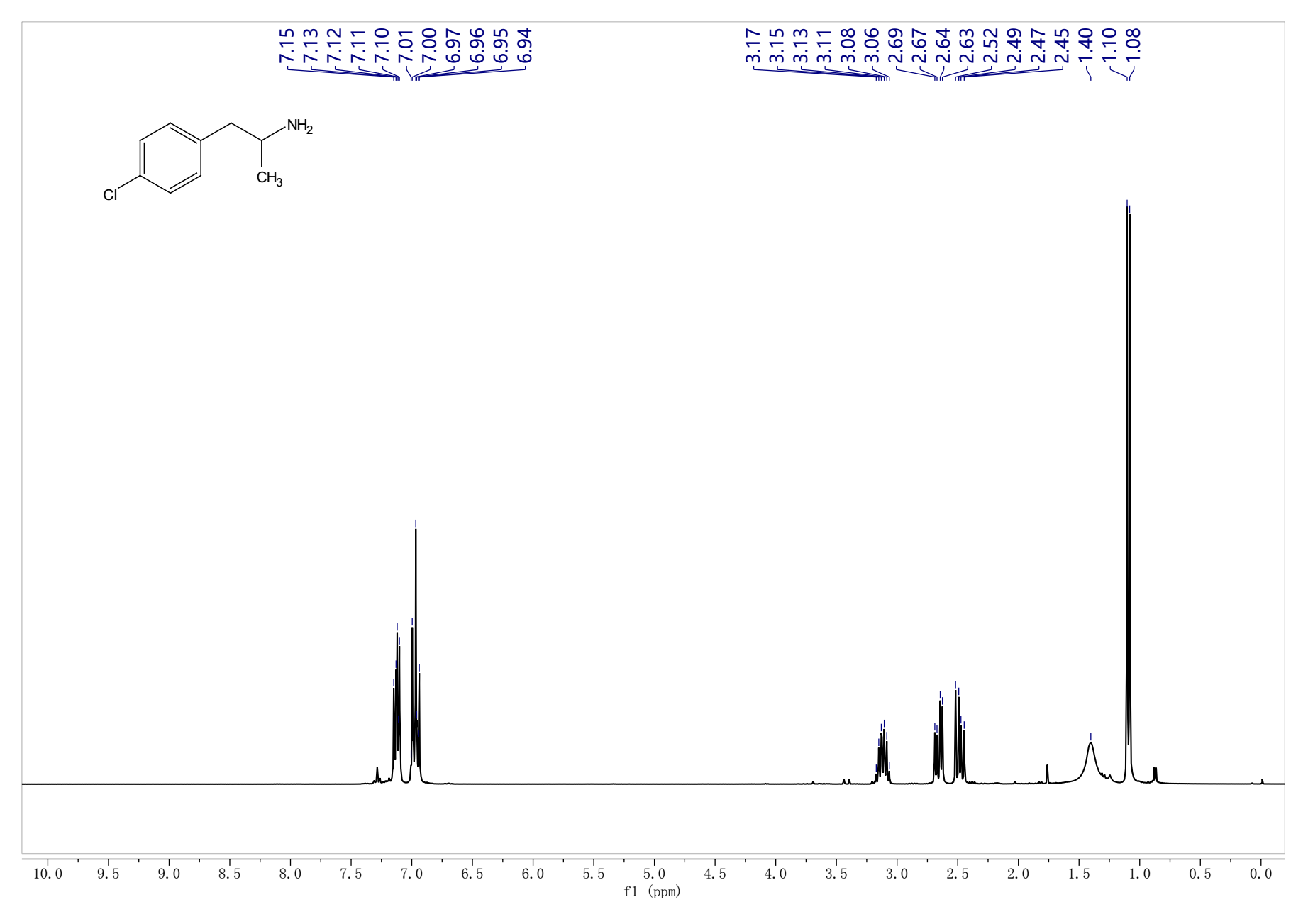


**Figure S64.** ^1^H NMR spectrum of **4-FA**


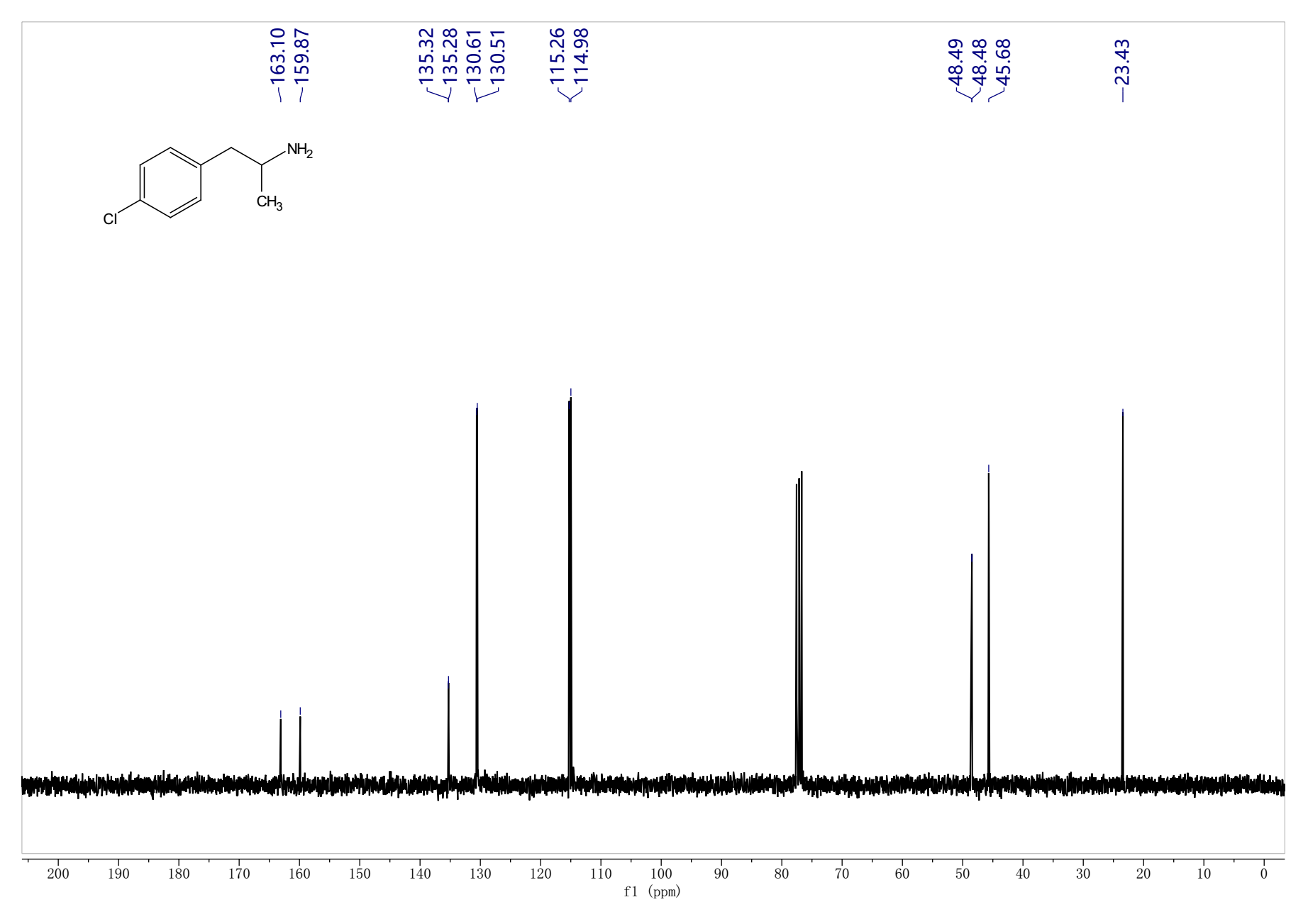


**Figure S65.** ^13^C NMR spectrum of **4-FA**


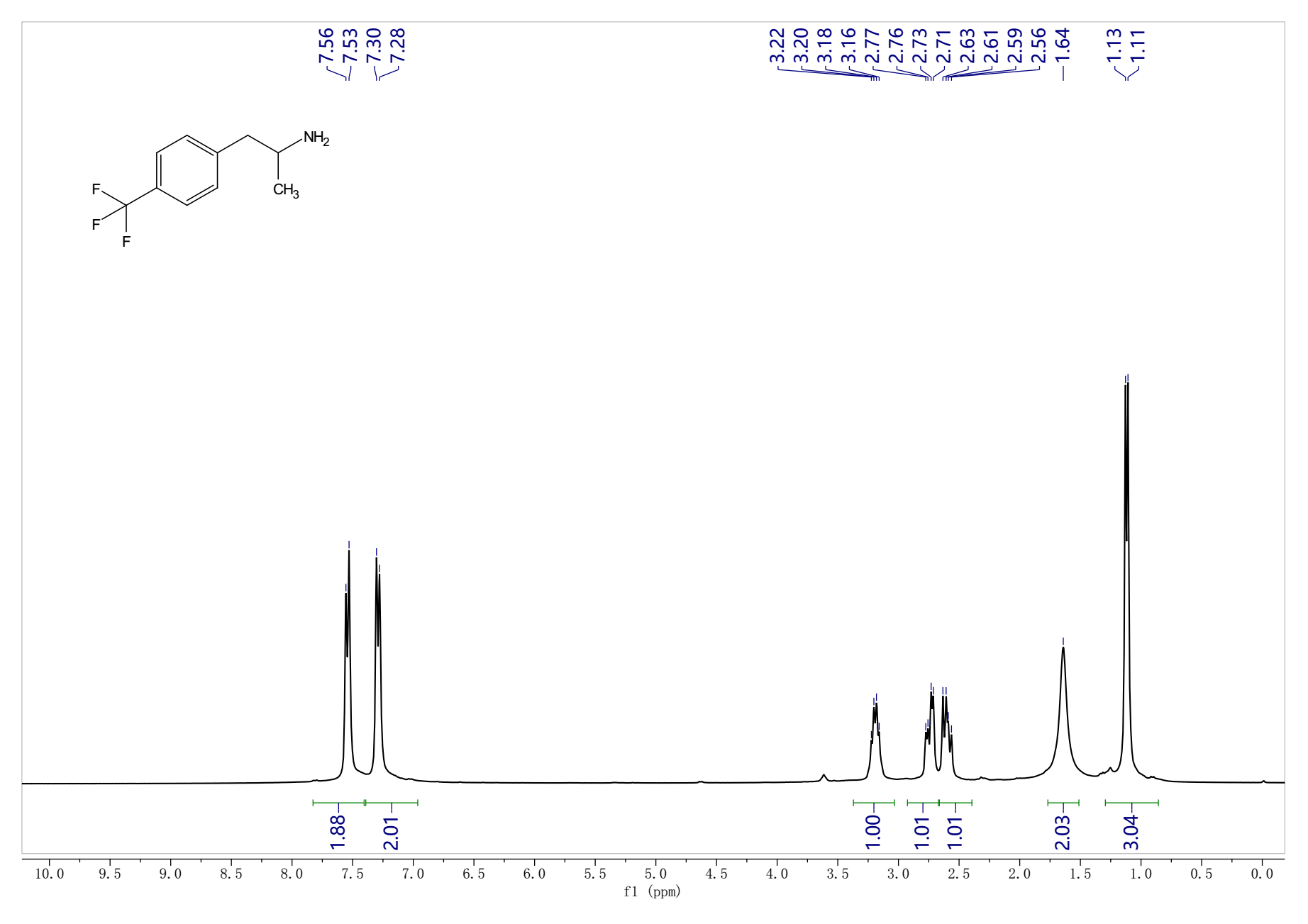


**Figure S66.** ^1^H NMR spectrum of **4-TFA**


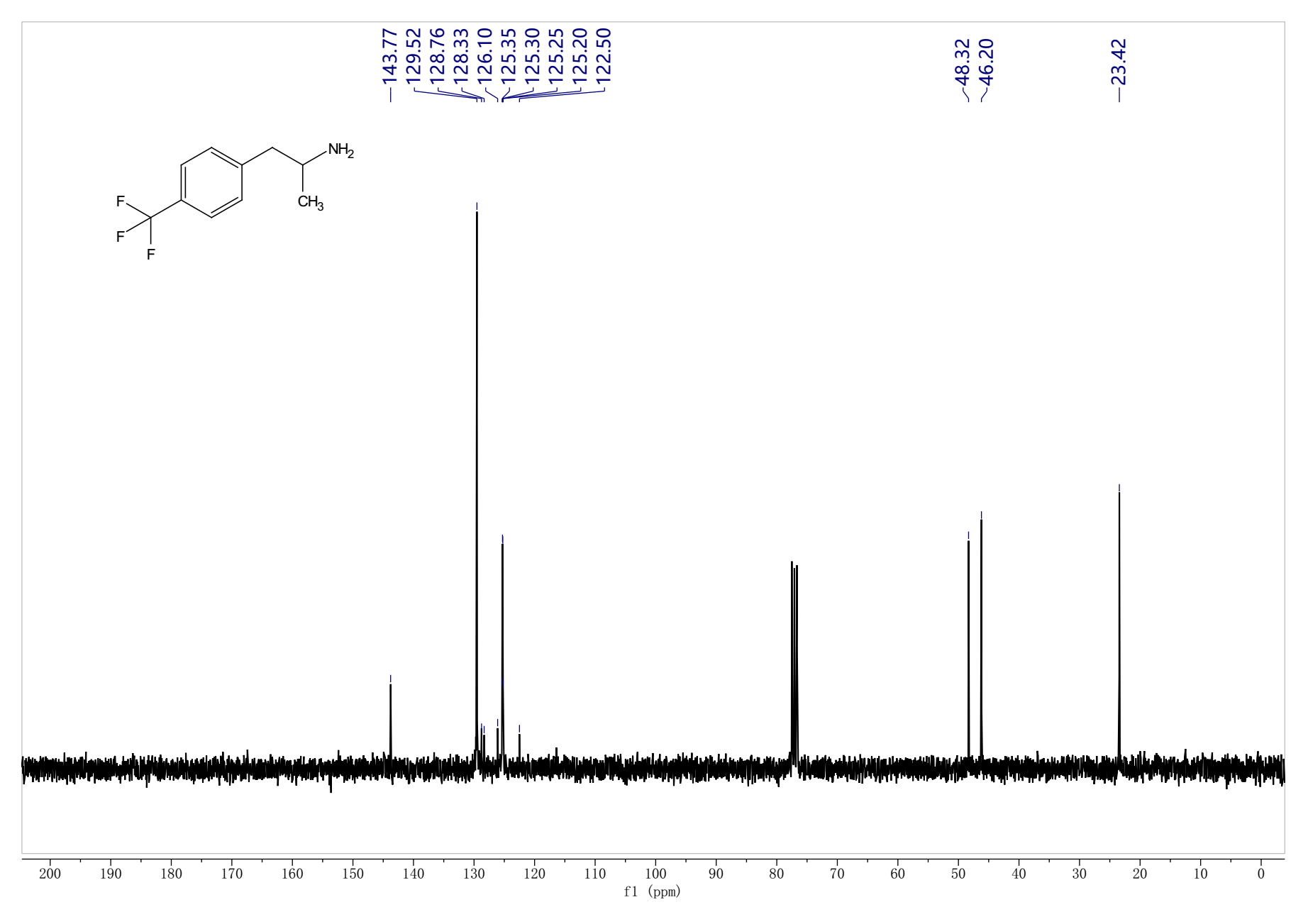


**Figure S67.** ^13^C NMR spectrum of **4-TFA**


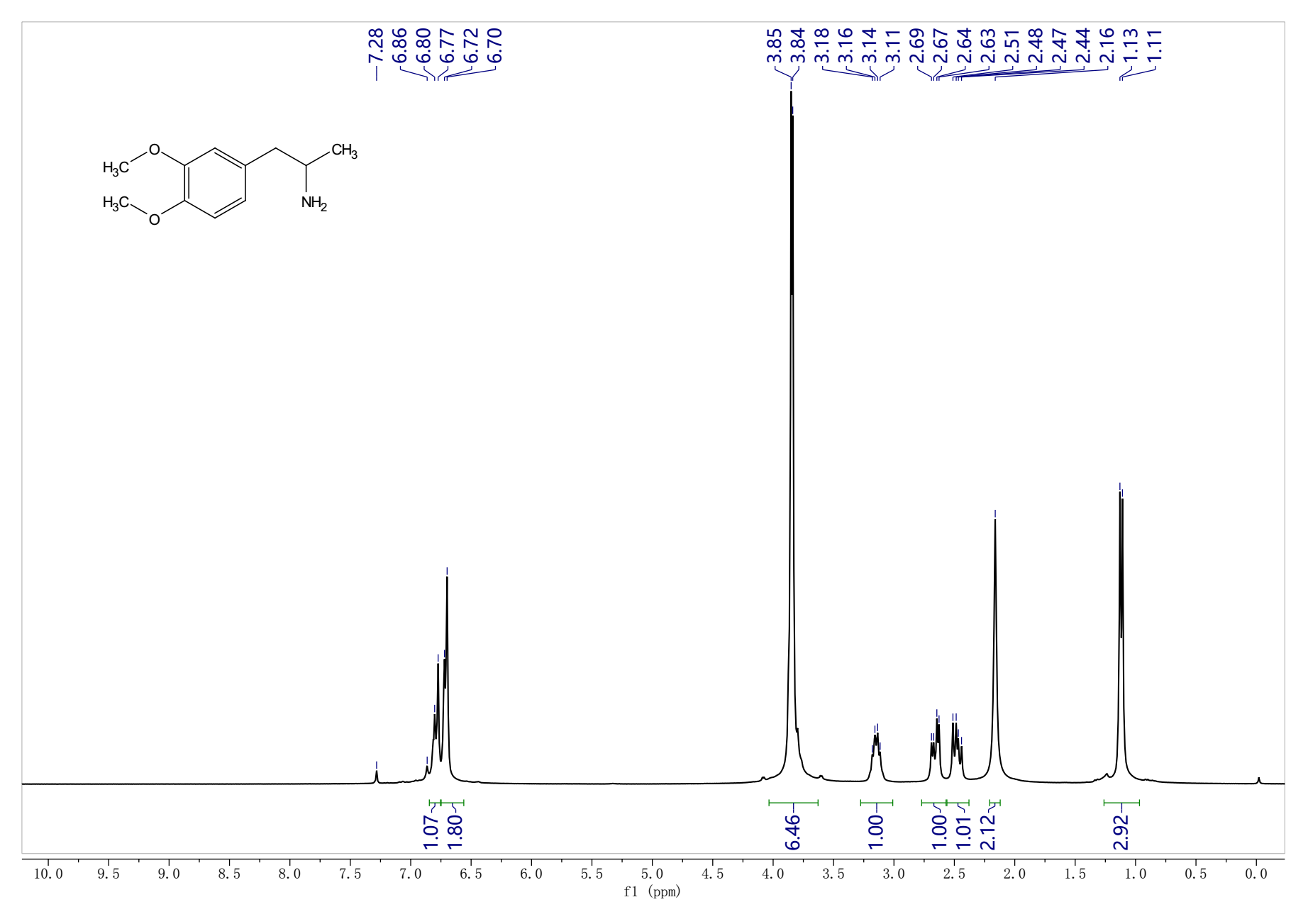


**Figure S68.** ^1^H NMR spectrum of **3,4-di-OMA**


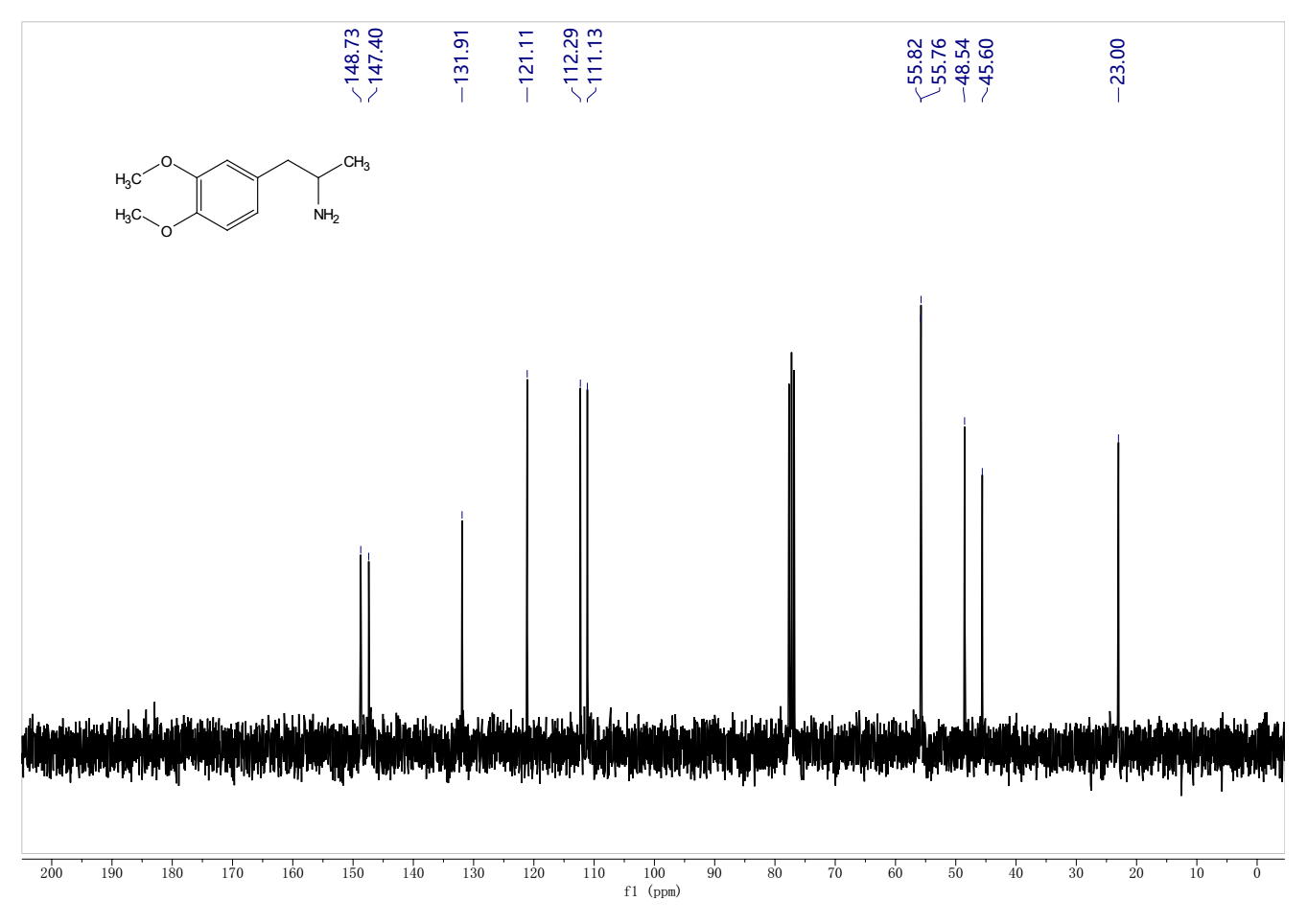


**Figure S69.** ^13^C NMR spectrum of **3,4-di-OMA**


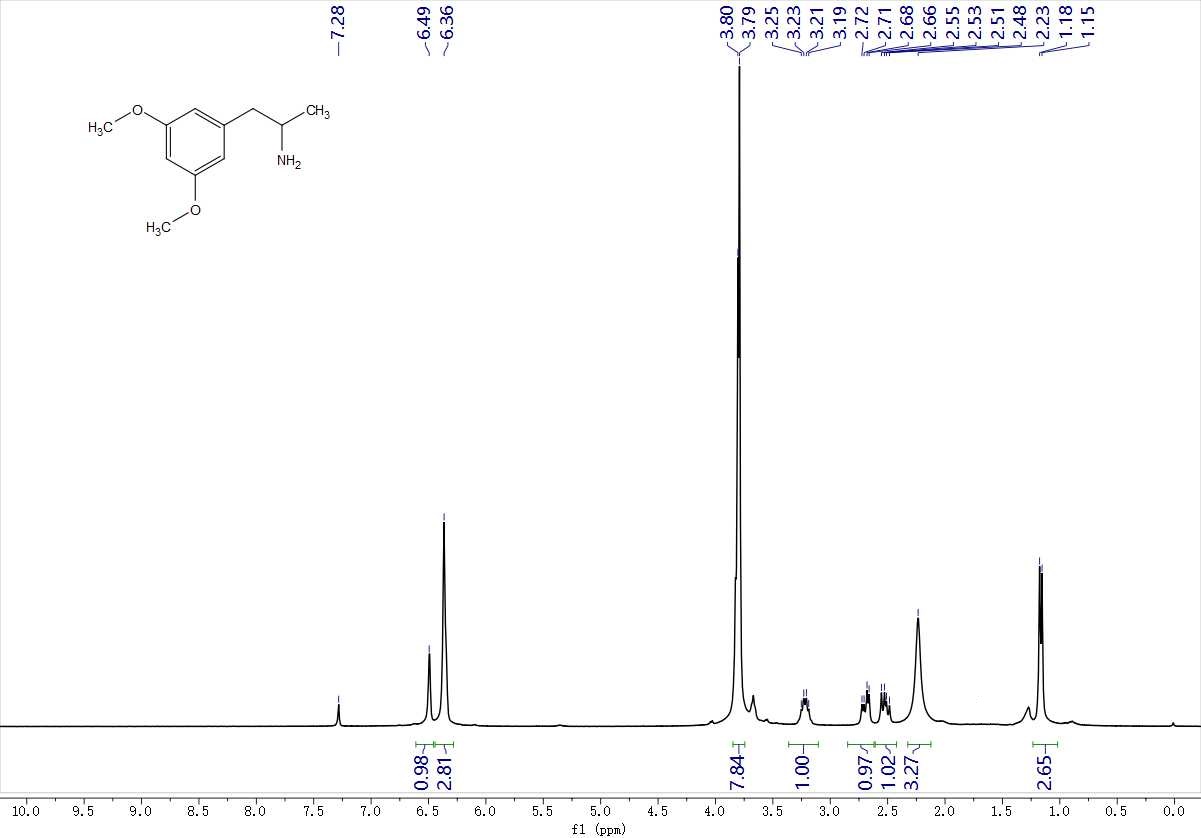


**Figure S70.** ^1^H NMR spectrum of **3,5-di-OMA**


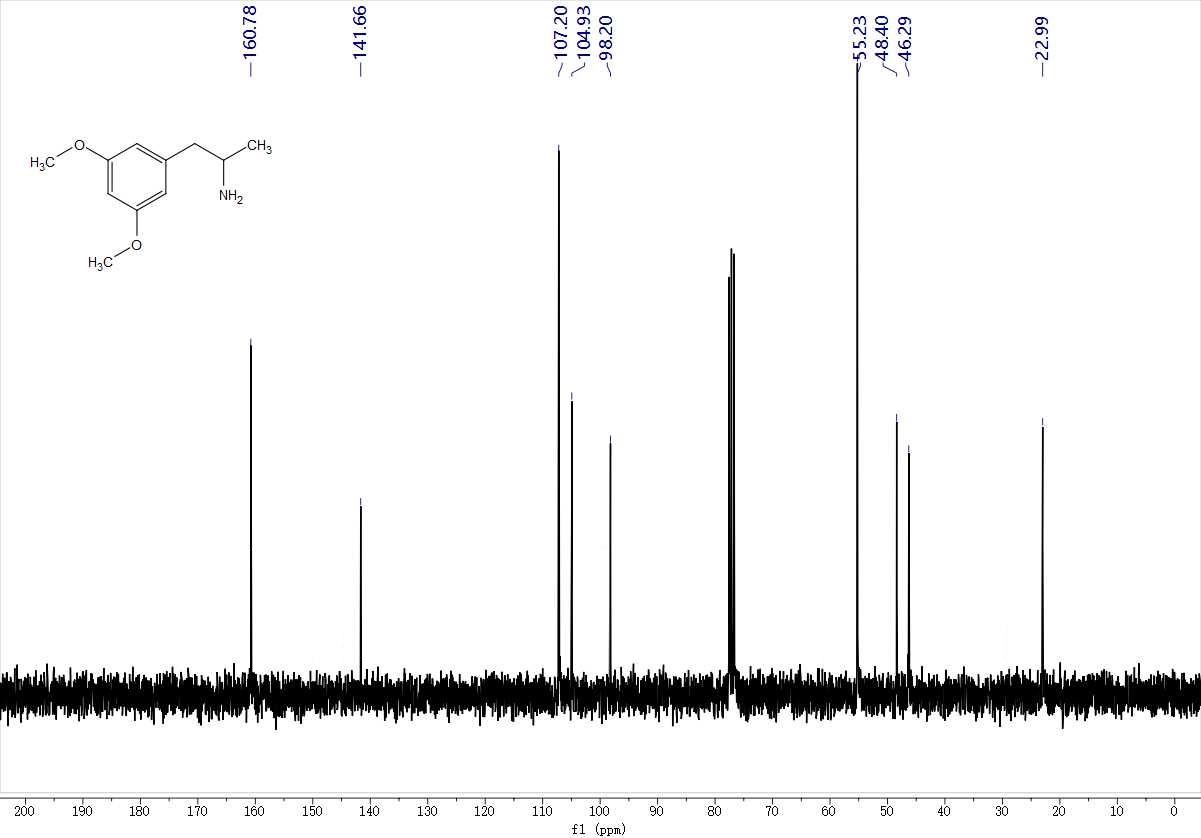


**Figure S71.** ^13^C NMR spectrum of **3,5-di-OMA**


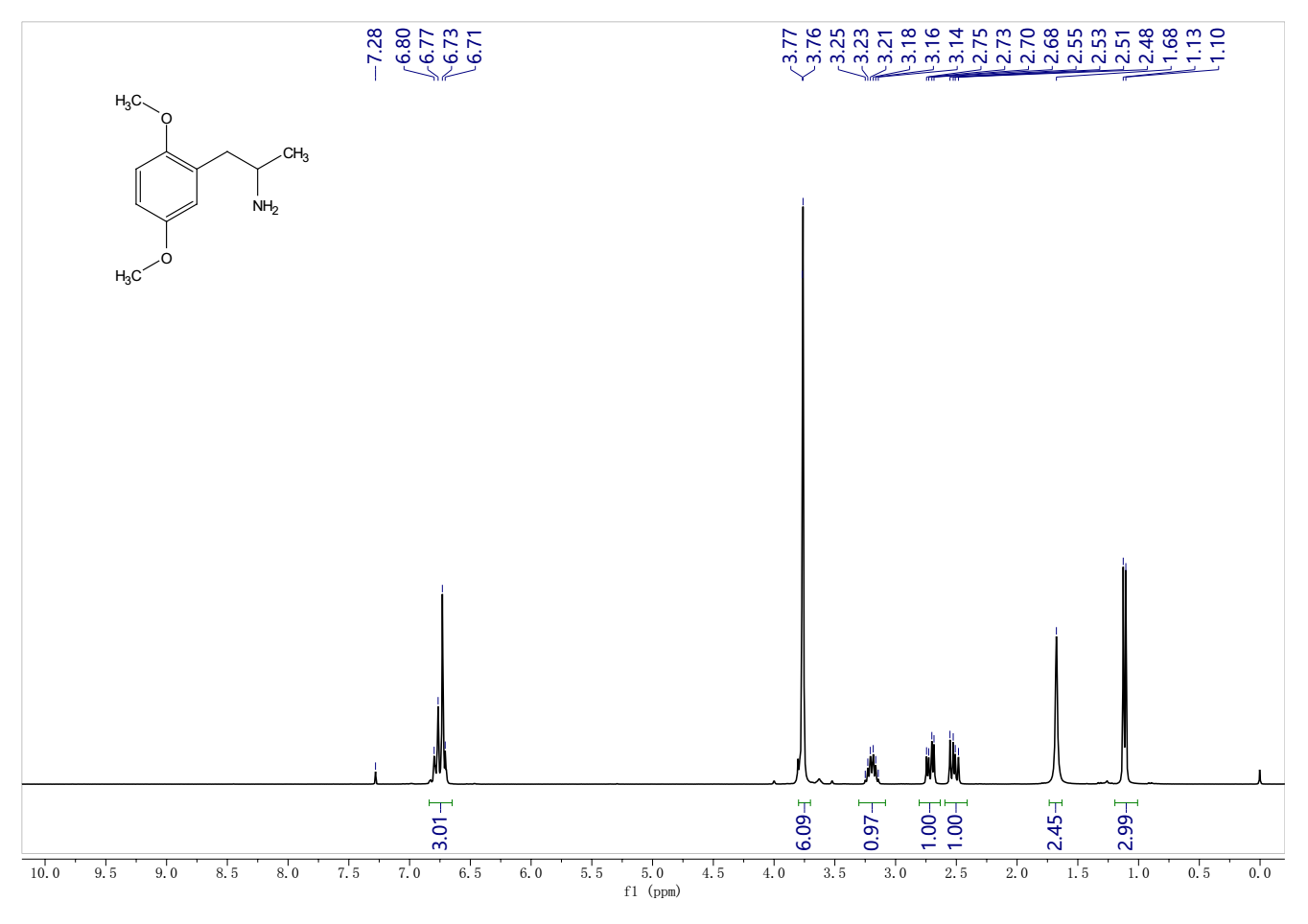


**Figure S72.** ^1^H NMR spectrum of **2,5-di-OMA**


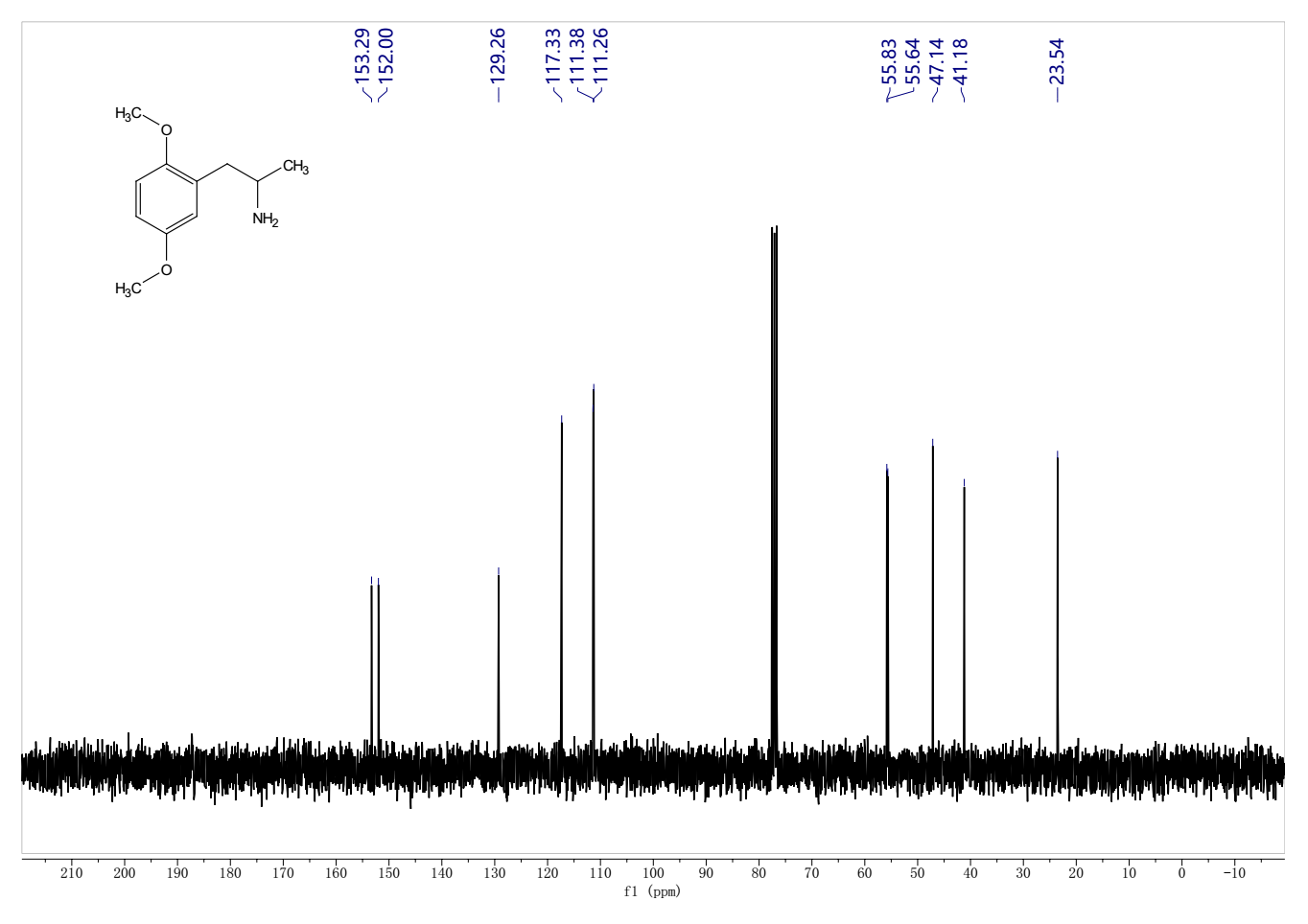


**Figure S73.** ^13^C NMR spectrum of **2,5-di-OMA**

**Behavioral Video**

**Video S1.** AMPH-induced SWIP in C. *elegans*. Day-1 adult *C. elegans* were transferred to 10 mM AMPH solution, and their SWIP behavior was recorded continuously for 10 minutes. The video is displayed at 30x speed.

**Video S2.** MDA-induced SWIP C. *elegans*. Day-1 adult *C. elegans* were transferred to 10 mM MDA solution, and their SWIP behavior was recorded continuously for 10 minutes. The video is displayed at 30x speed.

**Video S3.** METH-induced SWIP in C. *elegans*. Day-1 adult *C. elegans* were transferred to 10 mM METH solution, and their SWIP behavior was recorded continuously for 10 minutes. The video is displayed at 30x speed.

**Video S4.** MDMA-induced SWIP C. *elegans*. Day-1 adult *C. elegans* were transferred to 10 mM MDMA solution, and their SWIP behavior was recorded continuously for 10 minutes. The video is displayed at 30x speed.

**Video S5.** AMPH-induced CIP C. *elegans*. Day-1 adult *C. elegans* were transferred to an NGM plate containing 10 mM AMPH. CIP behavior was recorded continuously for 10 minutes, and the video is presented at 30x speed.

**Video S6.** MDA-induced CIP C. *elegans*. Day-1 adult *C. elegans* were transferred to an NGM plate containing 10 mM MDA. CIP behavior was recorded continuously for 10 minutes, and the video is presented at 30x speed.

**Video S7.** METH-induced CIP C. *elegans*. Day-1 adult *C. elegans* were transferred to an NGM plate containing 10 mM METH. CIP behavior was recorded continuously for 10 minutes, and the video is presented at 30x speed.

**Video S8.** MDMA-induced CIP C. *elegans*. Day-1 adult *C. elegans* were transferred to an NGM plate containing 10 mM MDMA. CIP behavior was recorded continuously for 10 minutes, and the video is presented at 30x speed.
